# Targeting of the nuclear RNA exosome to chromatin by HP1 affects the transcriptional programs of liver cells

**DOI:** 10.1038/s41467-026-72504-7

**Published:** 2026-04-29

**Authors:** Hiba Souaifan, Mickael Costallat, Laura Sitkiewicz, Kylian Godest, Florence Cammas, Carl Mann, Christian Muchardt, Christophe Rachez

**Affiliations:** 1 Sorbonne Université, CNRS, Laboratory of Computational, Quantitative and Synthetic Biology, CQSB, F-75005 Paris, France; 2 Sorbonne Université, CNRS, Inserm, Institut de Biologie Paris-Seine, IBPS, F-75005 Paris, France; 3 Ecole doctorale Complexité du Vivant, Sorbonne Université, Paris, France; 4 Institute of Human Genetics, CNRS UMR9002 University of Montpellier, 34396 Montpellier, France; 5 Institut de Biologie Intégrative de la Cellule (I2BC), CEA, CNRS, Université Paris-Saclay, 91190 Gif-sur-Yvette, France

**Keywords:** Chromatin, RNA metabolism, Cancer, Cancer microenvironment

## Abstract

Heterochromatin protein 1 (HP1), a hallmark of pericentromeric heterochromatin, is a chromatin-bound regulator of co-transcriptional processes including alternative splicing, but its role in RNA degradation remains unexplored. Here, we uncover a direct interaction between HP1 and nuclear RNA exosome complexes, major RNA decay machineries. In mouse embryonic liver cells, inactivation of all three HP1 isoforms leads to accumulation of retrotransposon-derived RNAs and stabilization of enhancer RNAs. These changes coincide with increased activity at a subset of liver enhancers particularly sensitive to reduced exosome activity, many of which regulate genes encoding extracellular matrix components such as collagen genes. Stratifying hepatocellular carcinoma samples by HP1 expression further reveal that tumors with low HP1 are marked by reduced RNA degradation, and increased expression of a similar subset of genes encoding extracellular matrix components and possibly contributing to tumor stiffness. These results suggest that HP1’s impact on RNA turnover contributes to its function in cancer biology.

## Abstract (web summary)

HP1 regulates chromatin and RNA processing, but its role in RNA decay was unclear. Here, the authors show that HP1 interacts with the nuclear RNA exosome to promote RNA degradation, linking reduced HP1 to enhancer RNA accumulation and extracellular matrix gene activation in liver cancer.

## Introduction

Chromatin structure is a major regulator of transcription. This regulation is mediated by molecular machineries that establish transcriptionally active or repressed chromatin states, known as euchromatin and heterochromatin, respectively. In active regions, chromatin influences gene expression programs that govern processes ranging from development to responses to external stimuli. In contrast, constitutive heterochromatin is typically found at pericentromeric regions and transposable elements, and is enriched with repressive epigenetic marks— notably di- and tri-methylation of histone H3 on lysine 9 (H3K9me2/3). These marks serve as binding sites for HP1 proteins, which are essential for the establishment, maintenance, and propagation of heterochromatin^1^.

The three mammalian HP1 isoforms, HP1α, HP1β, and HP1γ, share a conserved structure consisting of two globular domains separated by an intrinsically disordered hinge region. The N-terminal chromodomain specifically recognizes and binds H3K9me2/3-marked nucleosomes, anchoring HP1 to heterochromatic regions^2–4^. The C-terminal chromoshadow domain mediates dimerization and interactions with a broad range of PxVxL motif–containing proteins involved in transcriptional regulation, DNA replication, and repair^5,6^. The unstructured hinge region has an affinity for nucleic acids^7^. In addition to their well-established role in heterochromatin, HP1 proteins also localize to actively transcribed genes in both *Drosophila* and mammals, with isoform-specific patterns^8–10^. In euchromatin, HP1 proteins have been implicated in several co-transcriptional processes, including the regulation of alternative splicing^11–15^, recruitment of RNAi machinery to chromatin^12^, and control of transcriptional elongation^16^ and termination^17^.

Multiple lines of evidence indicate that the RNA-binding activity of HP1 proteins underlies their functions in both euchromatin and heterochromatin. In both compartments, HP1 proteins engage with repeated RNA sequences. This was first demonstrated by the requirement of RNA binding for HP1 targeting to heterochromatin^18,19^. Later, it was further shown that HP1α specifically associates with pericentromeric major satellite repeat RNA (SatRNA)^20^. More recently, we showed that HP1γ contributes to splicing precision by recognizing hexameric motifs enriched in intronic SINE elements, supporting intron–exon discrimination and contributing to accurate splice site definition^21,22^. In *Schizosaccharomyces pombe*, recognition of repeated sequences by the HP1 homolog Swi6 has been further linked to the degradation of satellite SatRNAs, contributing to transcriptional repression beyond chromatin condensation. Two distinct mechanisms may mediate this repression. First, Swi6 is required for RNAi-dependent silencing by directing SatRNA to the RITS complex, enabling siRNA production via double-stranded RNA formation^23^. Second, silencing at centromeric repeats and the silent mating-type locus—both heterochromatic regions—also involves polyadenylation-dependent RNA degradation via the RNA exosome cofactor Cid14^24^. Swi6 then engages in a dynamic exchange between free, chromatin-bound, and RNA-bound states, facilitating the delivery of repeat-derived RNAs to degradation machineries^25^. Notably, the Rixosome, identified as a Swi6-associated complex involved in XRN2-dependent RNA degradation, also contributes to RNA decay within heterochromatin^26^. However, HP1 proteins are not significantly enriched in purifications of the human Rixosome, which instead interacts with Polycomb subunits^27^.

The RNA exosome complex is a central machinery for RNA degradation and quality control in eukaryotic cells^28^. Its core structure consists of nine non-catalytic subunits (Exosc1–9) that form a scaffold for associated 3′ → 5′ ribonucleases. In the cytoplasm, the exosome partners with the ribonuclease Dis3l and cytoplasmic cofactors to form the SKI complex, which mediates mRNA turnover and surveillance^29^. In the nucleus, the exosome associates with two ribonucleases: Rrp6/Exosc10, a distributive exonuclease, and Rrp44/Dis3, a processive exo- and endonuclease. The nuclear exosome functions within specialized complexes, NEXT, PAXT, and TRAMP, that provide substrate specificity across a broad spectrum of nuclear RNAs^30^. The TRAMP complex, well characterized in *S. cerevisiae*, contributes to ribosomal RNA processing and snoRNA precursor degradation. In mammals, TRAMP is primarily restricted to the nucleolus^31^. By contrast, the NEXT complex targets unprocessed RNAs with a free 3′ end, such as promoter-upstream transcripts (PROMPTs, uaRNAs), and enhancer RNAs (eRNAs)^32–34^, while the PAXT complex directs degradation of polyadenylated nuclear RNAs^35^. A defining feature of all exosome complexes is the presence of an RNA helicase. In the cytoplasm, this activity is provided by Ski2, while in the nucleus, Mtr4 is essential for substrate recruitment to NEXT, PAXT, and TRAMP (reviewed in ref. ^36^). Recruitment of these nuclear complexes is further mediated by the cap-binding complex (CBC), which recognizes the 5′ methyl-guanosine cap of RNA polymerase II transcripts. CBC, along with its cofactor ARS2, connects to NEXT via the zinc-finger protein ZC3H18, forming the CBC–NEXT axis^37^. ZC3H18 bridges the 5′ cap to the exosome, facilitating the degradation of unstable or aberrant RNAs.

The activity of the RNA exosome has previously been linked to the organization of chromatin structure. At a large scale, chromatin architecture, particularly topologically associating domains (TADs) and chromatin loops, is shaped by architectural proteins such as CTCF and cohesins^38^. Depletion of the nuclear exosome subunit DIS3 impairs non-coding RNA processing, reduces CTCF binding, and disrupts TAD organization at the *Igh* locus^39^. In parallel, transcripts derived from normally silenced retroelements are targeted for degradation by the NEXT complex, which recognizes unstable RNAs arising from heterochromatic regions^40,41^. Mass spectrometry (MS) approaches have further suggested that RNA exosome-associated factors such as ZC3H18 and the RNA helicase MTR4 may associate with HP1^42,43^, while, reciprocally, HP1 MS data have detected ZC3H18 among the interacting partners of all three HP1 isoforms^44^. This association suggests that molecular bridges may exist between chromatin, HP1, and nuclear RNA degradation machineries.

Building on this possible connection, we have here analyzed the relationship between chromatin-associated HP1 proteins and RNA exosome components in murine BMEL liver cell lines depleted of all three HP1 isoforms. Previous studies using these cells showed that HP1 depletion results in the accumulation of transcripts from normally silenced retroelements, including LINEs, SINEs, and LTR-containing endogenous retroviruses (ERVs)^45,46^. In this context, we confirm physical associations between HP1 proteins and several representative components of the nuclear exosome, and show an HP1-dependent enrichment of exosome complexes at chromatin loci. HP1 loss also led to a marked increase in chromatin-associated unstable transcripts, such as PROMPTs and retroelement-derived RNAs, particularly at LTR loci. Notably, some of these transcripts extended beyond the annotated repeat boundaries and were detected in a mature form in the cytoplasm. In parallel, enhancers located near these retroelements became activated in the absence of HP1, leading to upregulation of adjacent genes and widespread alterations in transcriptional programs.

## Results

### HP1 proteins interact with components of the nuclear RNA exosome complexes

**Fig. 1:**
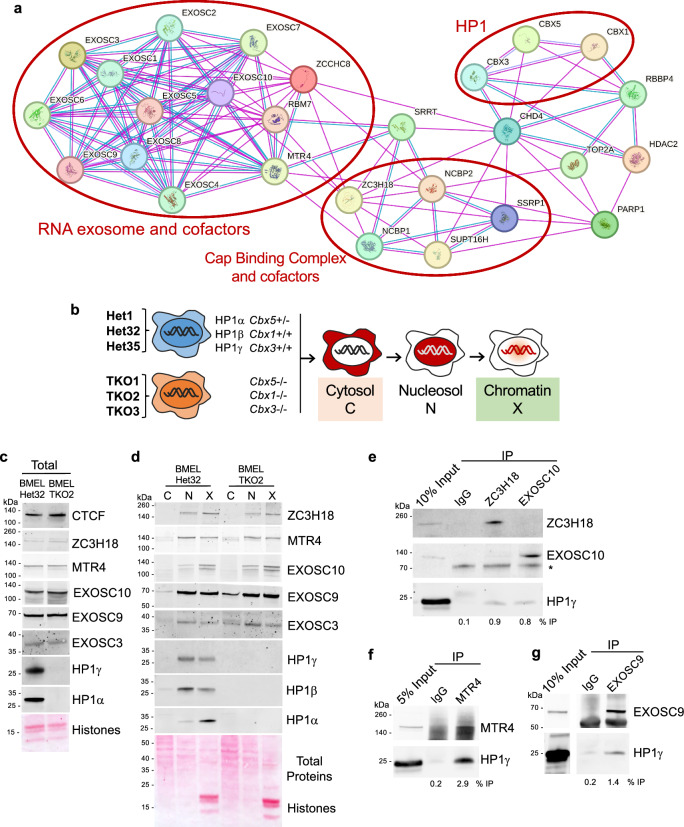
Protein-protein association between HP1 and the RNA exosome. **a** STRING analysis of known physical associations of proteins among factors co-precipitating with ZC3H18 proteins, identified by interaction profiling of ZC3H18^43^. Pink and blue network edges correspond to protein associations based on experiments or curated databases, respectively. **b** Genotype and subcellular fractionation scheme of the six BMEL clones expressing (Het) or not (Triple Knock-out, TKO) HP1 proteins used in this study. **c** Protein levels in total extracts, and (**d**) subcellular distribution in the three fractions, cytosolic (C), nucleosoluble (N), and chromatin (X), depicted in Fig. 1b of indicated exosome subunits and cofactors in Het and TKO cells by Western blot analysis and Ponceau staining of total proteins. **e**–**g** Co-immunoprecipitation of HP1γ by ZC3H18, EXOSC10, MTR4, or EXOSC9 in BMEL nuclear extracts. % IP, quantitation of HP1γ IP efficiency versus 100% input. Both input and IP panels in Fig. 1g are split lanes from the same gel and same membrane. Blots are representative of *n* = 3 (**c**–**e**, **g**) or 4 (**f**) independent experiments. Source data are provided as a Source Data file.

Analysis of ZC3H18 immunoprecipitation–mass spectrometry data^43^ using the STRING database (Search Tool for the Retrieval of Interacting Genes/Proteins) documented that the protein-protein interactions linking the RNA exosome and the Cap binding complex with the three HP1 proteins and other epigenetic regulators, were supported by multiple independent sources (Fig. 1a, each “string” represents a documented protein-protein interaction). This prompted us to further investigate the links between HP1 proteins and the nuclear RNA exosome complexes.

To this end, we compared mouse embryonic liver (BMEL) cells lacking all three HP1 isoforms (triple knockout, TKO) to HP1-expressing controls (Het, heterozygous for HP1α and wild type for HP1β and HP1γ). Six independent clones were analyzed: TKO1, TKO2, TKO3, and Het1, Het32, Het35 (Fig. 1b and Supplementary Fig. 1a). Western blot analysis of total cell extracts showed comparable protein levels of the core exosome subunits EXOSC3, EXOSC9, and EXOSC10, as well as of MTR4, a nuclear RNA helicase shared by all nuclear RNA exosome complexes, and ZC3H18, a zinc-finger adaptor that recruits the NEXT complex, in Het and TKO clones. These data indicate that HP1 depletion does not alter the overall abundance of these exosome components (representative clones shown in Fig. 1c). Upon fractionation of the Het clones into cytosolic (C), nucleosolic (N), and chromatin (X) extracts, the five proteins were predominantly detected in the nucleosolic and chromatin fractions, which were likewise enriched in HP1α, HP1β and HP1γ (Fig. 1d). Fractionation quality was validated by Western blotting for marker proteins and by analyzing the distribution of representative cytoplasmic and nuclear transcripts (Supplementary Fig. 1a–c). In the TKO clones, EXOSC9 and EXOSC3 appeared enriched in the cytosolic fraction compared with Het cells (Fig. 1d, columns C). Quantification of EXOSC9 distribution across cytosolic, nucleosolic, and chromatin fractions in all Het and TKO clones demonstrated a statistically significant increase in the cytosolic and soluble nuclear compartments in TKO cells, however with no significant difference detected in the chromatin fraction (Supplementary Fig. 1f, g). EXOSC3 redistribution was not quantified. Together, these data suggest a modest but reproducible increase in EXOSC9 levels in the soluble compartments upon HP1 depletion.

Finally, co-immunoprecipitation from nuclear extracts containing both soluble and chromatin-associated material confirmed an association of HP1 proteins with the nuclear RNA exosome complexes and their associated cofactors, as detected using EXOSC9-, EXOSC10-, MTR4-, and ZC3H18-antibodies, consistent with the previously reported mass spectrometry data (Fig. 1e–g and Supplementary Fig. 1d, e). These interactions were rigorously validated by demonstrating the absence of antibody cross-reactivity in the immunoprecipitation assays (Supplementary Fig. 2), and by performing siRNA-directed knockdown experiments showing that the extent of HP1 co-immunoprecipitation was correlated with the abundance of each of the four proteins used for immunoprecipitation (Supplementary Figs. 3, 4). These interactions were maintained following RNase treatment (Supplementary Fig. 1h–i), indicating that they were not mediated by RNA. Together, these results further documented interactions between the HP1 proteins and components of the RNA exosome machinery, and suggested that these interactions are required for full nuclear retention of certain exosome subunits.

### Several unstable RNA species are found stabilized in the absence of HP1 proteins, in both chromatin and cytosolic fractions

We next used RNA-seq to examine RNA profiles in chromatin and cytosolic fractions of the BMEL Het and TKO clones (Fig. 1b). In this setup, the chromatin fraction was expected to be enriched in nascent and incompletely processed RNAs, whereas the cytosolic fraction was expected to be depleted of unstable nuclear transcripts and enriched in mature mRNAs (Supplementary Fig. 1c). In parallel, RNA-seq was also performed on total RNA from each clone (Supplementary Figs. 1, 5a).

The RNA species we examined included various RNAs encoded by LTRs, LINEs, SINEs, and MMSAT repeats. For these, we focused on extragenic repeat elements, excluding those located within gene bodies, such as intronic repeats, which may be transcribed incidentally as part of coding gene expression and processed in that context. We also examined levels of upstream antisense uaRNAs at promoters (also known as PROMPTs), and of eRNAs at active distal enhancers (enhD), all verified to be less stable than a reference *Rplp0* mRNA (Supplementary Fig. 5b). Genomic elements were considered as expressed when yielding at least two RNA-seq reads in at least one experimental condition.

**Fig. 2:**
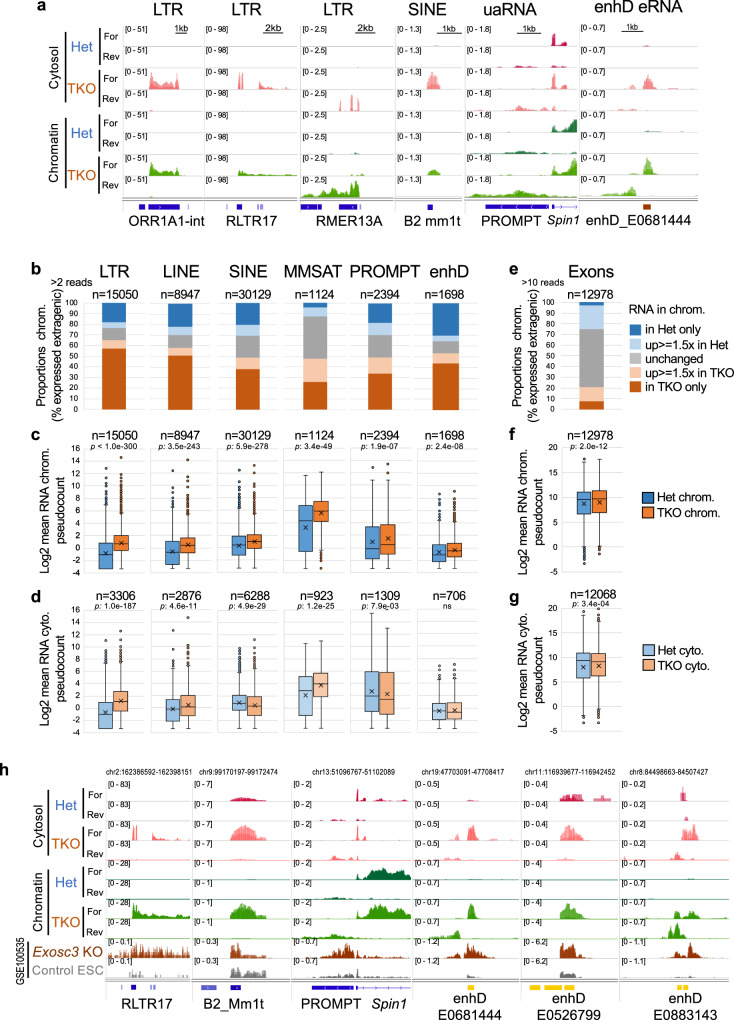
Chromatin-enriched RNA is increased in TKO cells and stabilized in the cytoplasm. **a** Genome views of RNA density profiles (overlaid triplicates) of forward (For) and reverse (Rev) read stands in chromatin (green) and cytosolic (red) fractions, at indicated genomic loci showing TKO-specific or TKO-increased RNA. **b**–**d** RNA levels by counting uniquely mapped reads on repeated elements (LTR, LINE, SINE, MMSAT4), Promoter upstream antisense RNA (PROMPT), distal enhancers (enhD) and exons of ensembl genes. **b** Cumulative histograms of proportions of the indicated genomic regions expressing RNA (more than 2 reads) by comparison of the three Het clones with the three TKO clones. Five categories discriminate expression on chromatin in TKO only (brown), increased in TKO (salmon), in Het only (dark blue), increased in Het (light blue), unchanged (grey). **c**, **d** Boxplots of mean RNA read counts in chromatin (C) or in the cytosol (D) on genomic regions as in B. in Het (blue) or TKO (orange). *p* values are unpaired two-tailed Student’s t-test (ns, not significant). **e**–**g** Histogram and boxplots as in panels **b**–**d** for proportions and counts on expressed exons with more than 10 reads. *p* values are unpaired two-tailed Student’s t-test. **h** Transcriptome profiles in Het or TKO chromatin (green) or cytoplasm (red) compared with transcriptomes of mESC invalidated for the expression of the EXOSC3 core exosome subunit (*Exosc3* KO, brown profiles) and control mESC (grey profiles) (GSE100535 dataset^48^). All boxplots in Fig. 2 show median (central line), IQR (25th and 75th percentiles, box limits); whiskers extend to 1.5 x IQR.; points are values of outliers. Source data are provided as a Source Data file.

Using this criterion, levels of unstable transcripts increased in TKO cells compared to HP1-expressing (Het) cells. In particular, we noted that for more than 50% of all expressed LTR elements, expression was detected exclusively in TKO cells (see examples in Fig. 2a, and quantification in Fig. 2b). Several elements were also expressed exclusively in Het cells, reflecting the multiplicity of HP1 functions not solely linked to RNA stability^47^. Global analysis of RNA abundance further showed that mean RNA levels from PROMPTs, distal enhancers (enhD), and repeat elements (LTRs, LINEs, SINEs) were increased in the chromatin fraction of TKO cells relative to Het (Fig. 2c). In contrast, chromatin-associated pre-mRNAs from Ensembl-annotated genes, measured by exonic reads, were less affected by HP1 depletion. Globally, the Ensembl gene transcript category showed more moderate changes in both RNA levels and the number of loci with TKO- or Het-specific expression compared to unstable RNA categories (Fig. 2e–g). These findings suggest that HP1 loss predominantly affects the expression and/or stability of unstable RNAs, rather than processed mRNAs. Supporting this, several unstable RNAs that accumulated in the chromatin fraction—particularly those derived from LTRs, LINEs, and MMSAT repeats—were also found at higher levels in the cytoplasm of TKO cells (Fig. 2d). In addition, many LTR-initiated transcripts extended for several kilobases and, interestingly, some appeared to be spliced in the cytoplasm (e.g., RLTR17, RMER13A; Fig. 2a), suggesting that in the absence of HP1, these RNAs evade nuclear decay and undergo canonical maturation.

Notably, several of the LTR-derived transcripts most readily detected in TKO cells (Fig. 2h, green and red profiles) also accumulated upon depletion of EXOSC3, a core subunit of the nuclear RNA exosome, in publicly available RNA-seq data from mouse embryonic stem cells (ref. ^48^; Fig. 2h, brown profiles). Although derived from a different cell type, this overlap supports the idea that the accumulation of a number of unstable transcripts observed in TKO cells reflects impaired exosome-mediated RNA degradation. Additionally, several unstable transcripts that accumulated in TKO cells were similarly increased in Het cells following siRNA-mediated depletion of RNA exosome components, whereas stable mRNAs such as *Rplp0* and *Actb* mRNAs remained unaffected (Supplementary Fig. 6). Together, these results suggest that HP1 loss stabilizes normally unstable RNAs, possibly due to defective degradation at the chromatin level.

### An HP1-dependent association of nuclear RNA exosome and cofactors to chromatin loci

**Fig. 3:**
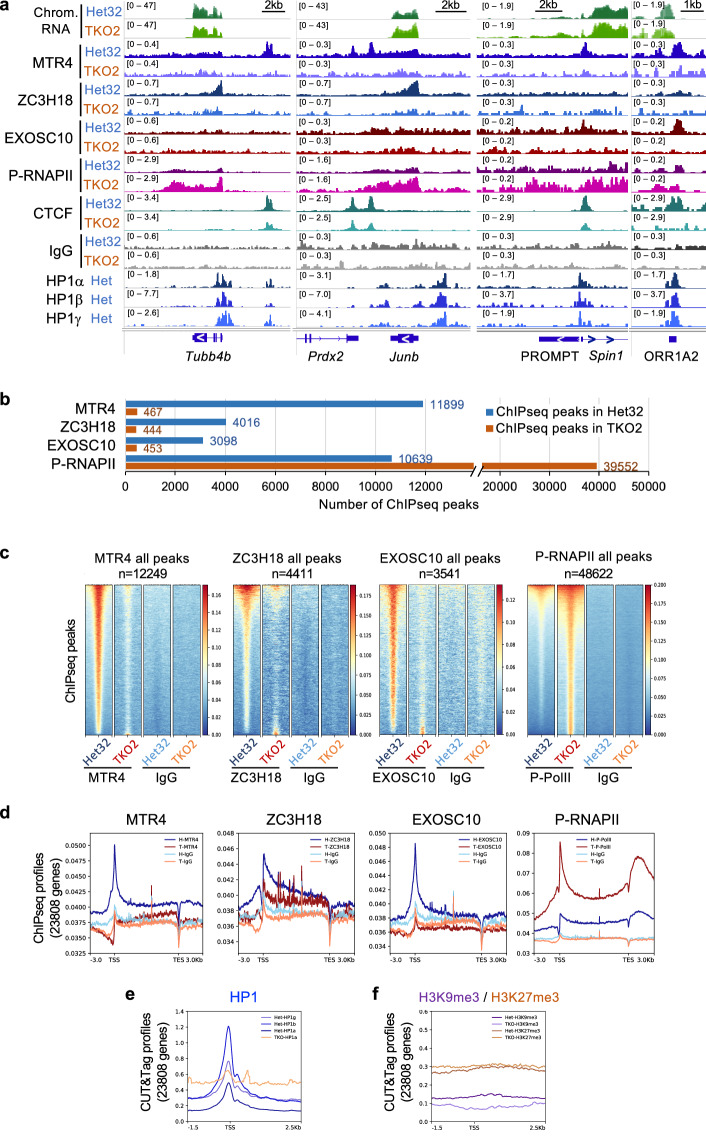
ChIP-seq analysis reveals an HP1-dependent targeting of Nuclear RNA exosome and cofactors to chromatin loci. **a** Genome views of chromatin RNA density profiles (top), ChIP-seq profiles of indicated proteins, and HP1 CUT&Tag density profiles (bottom) on the indicated loci. **b** Number of ChIP-seq peaks versus IgG defined by MACS2 analysis for the indicated proteins in Het32 (blue) and TKO2 (brown). **c** Heatmaps of genome-wide ChIP-seq densities centered on ChIP-seq peaks in both Het32 and TKO2 combined in a single list (all peaks). **d** Metagene analysis of ChIP-seq profiles of the indicated proteins on 23808 gene bodies defined as TSS to TES intervals, in Het32 (dark blue), in TKO2 (dark red), compared to IgG profiles in Het32 and TKO2 (salmon and light blue profiles, respectively). **e** CUT&Tag profiles at gene TSS for the three HP1 proteins (E) in Het (blue profiles), and HP1α in TKO (salmon profile). **f** CUT&Tag profiles at TSS for H3K9me3 (purple) and H3K27me3 (brown) histone marks in Het (dark colors) and in TKO (light colors).

Having observed that HP1 inactivation leads to the accumulation of unstable RNAs on chromatin, we next sought to examine more closely how RNA exosome and cofactors associate with nuclear structures. We therefore performed chromatin immunoprecipitation followed by high-throughput sequencing (ChIP-seq) with anti-EXOSC10, -MTR4, and -ZC3H18 antibodies using the Het32 and the TKO2 clones. To also gain insight into the overall impact of HP1 inactivation on chromatin structure, we completed this series of experiments with anti-CTCF, and anti-phospho RNA polymerase II (P-RNAPII, as a mix of both phospho-Ser2 and -Ser5 forms) ChIP-seq, and HP1α, HP1β, HP1γ, H3K9me3, and H3K27me3 CUT&Tag experiments (Fig. 3a).

Examination of the ChIP-seq profiles with a genome browser revealed that the nuclear exosome core subunits EXOSC10 and MTR4, and the cofactor ZC3H18 were actively recruited to chromatin at specific genomic loci. This recruitment was most clearly detected in regions also recruiting P-RNAPII, while MTR4 additionally displayed an interesting overlap with CTCF. Importantly, focal enrichment of EXOSC10, MTR4, and ZC3H18 at specific chromatin loci was markedly reduced in TKO2 cells, as evidenced by loss of ChIP-seq peaks. In contrast, levels of phosphorylated RNA polymerase II (P-RNAPII) appeared increased in TKO2 cells (Fig. 3a). Quantification of MTR4, ZC3H18, and EXOSC10 ChIP-seq peaks detected with the MACS2 package confirmed a dramatic decrease in the number and/or intensity of these peaks upon inactivation of the HP1 genes, while the total number of P-RNAPII peaks was strongly increased (Fig. 3b, c).

Metagene analysis on more than twenty thousand genes confirmed a preferential enrichment of EXOSC10, MTR4, ZC3H18 on the body of genes, with a peak at the transcription start site (TSS - Fig. 3d). Thus, the distribution of these factors coincided with that of the P-RNAPII, although missing accumulation at sites of transcription termination (TES). This analysis further showed that HP1 inactivation was associated with a global decrease in EXOSC10, MTR4, and ZC3H18 occupancy across gene bodies, whereas RNAPII occupancy was increased over the same regions (Fig. 3d, compare brown and dark blue profiles).

Importantly, increased RNAPII signal at TSSs in TKO cells did not correlate with increased transcriptional output. Metagene analysis stratifying genes according to expression changes between Het and TKO showed that, whereas RNAPII signal across gene bodies was preferentially elevated at upregulated genes, comparable RNAPII accumulation at TSSs was observed at both up- and downregulated genes (Supplementary Fig. 7e, f, compare brown profiles). Thus, RNAPII signal at TSSs of TKOs may reflect increased promoter-proximal stalling rather than increased transcriptional initiation.

CUT&Tag analysis of the recruitment of each HP1 isoform in Het cells showed their preferential enrichment at gene transcriptional start sites (TSS, blue profiles Fig. 3e), compared to the background HP1α CUT&Tag signal in TKO cells (orange profile Fig. 3e). This distribution of HP1 proteins at actively transcribed genes is consistent with earlier reports^49^. It is also compatible with a co-recruitment of HP1 and RNA exosome components at promoter regions. Of note, although HP1 enrichment at TSS was not associated with H3K9me3 or H3K27me3 marks, HP1 colocalized with H3K9me3 at other regions, such as simple repeats (Fig. 3e, f, and Supplementary Fig. 7a, b). At these sites, HP1 inactivation did not lead to a loss of H3K9me3 but rather to a redistribution of the mark across repeats. These observations suggest that the targeting of the RNA exosome and its nuclear cofactors to TSS by HP1 and its association with H3K9me3 at other loci rely on two independent mechanisms.

#### HP1 concentrates MTR4 and CTCF at gene promoters

**Fig. 4:**
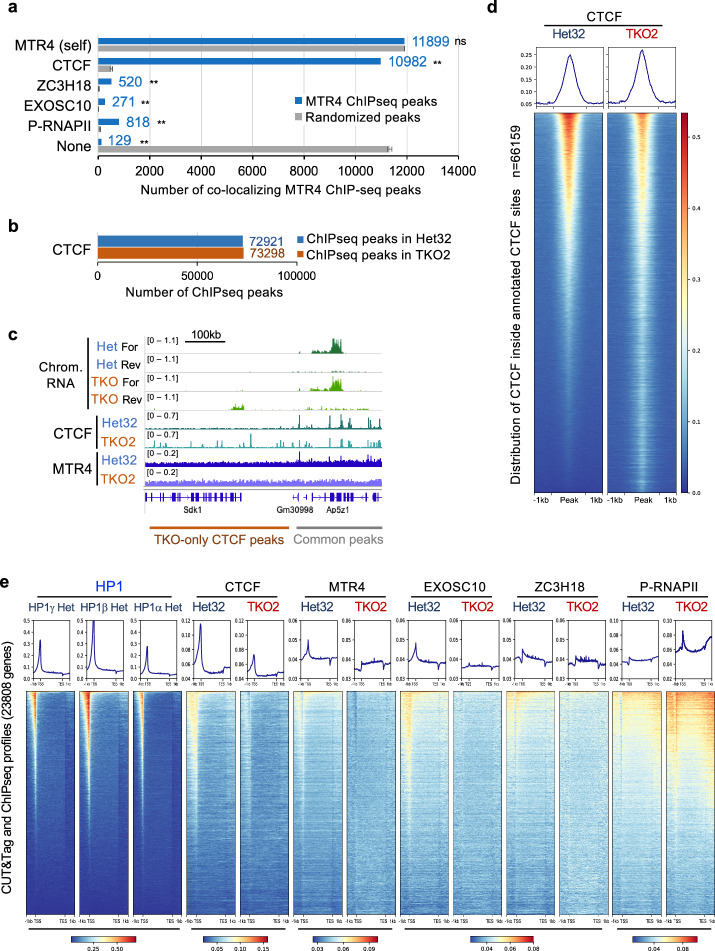
HP1-dependent CTCF and MTR4 at promoters. **a** Number of MTR4 ChIP-seq peaks in Het32 (blue bars) as in Fig. 3b, colocalizing or not (None) with the other indicated ChIP-seq peaks from Figs. 3b, 4b, compared to the mean number of colocalizations +/- SD of the same list of MTR4 ChIP-seq peaks randomized (200 times shuffle) on their genomic locations (gray bars). Statistical significance of colocalizing peaks was assessed using a rank-based permutation test (*n* = 200 randomizations), and empirical *p* values were computed from the null distribution. **, *p* = 0.005; ns, *p* > 0.05. **b** Number of CTCF ChIP-seq peaks versus IgG defined by MACS2 analysis in Het32 (blue) and TKO2 (brown). **c** Genome view of chromatin-enriched RNA (as in Fig. 2a), CTCF and MTR4 densities at a locus showing two regions bearing TKO-only CTCF peaks (on the left), or peaks common to both cell types (on the right). **d** Heatmaps of genome-wide CTCF ChIP-seq densities in Het32 and TKO2 centered on all CTCF ChIP-seq peaks. **e** Heatmaps of ChIP-seq densities of the indicated proteins on 23808 gene bodies defined as TSS to TES intervals, in Het32 (dark blue), in TKO2 (dark red). Source data are provided as a Source Data file.

We next examined in greater detail the colocalization of MTR4 with CTCF binding sites. Quantitative analysis showed that ~90% of MTR4 peaks overlapped CTCF peaks, representing ~15% of all CTCF sites (Fig. 4a). Notably, MTR4 was the only exosome-associated factor analyzed that displayed this preferential overlap. Comparison of ChIP-seq profiles between Het32 and TKO2 cells revealed that the overall number of CTCF peaks remained largely unchanged upon HP1 depletion (Fig. 4b). However, peak distribution was altered, with reduced signal at a subset of loci and the appearance of new CTCF peaks elsewhere (Fig. 4c, d). Genome browser inspection indicated that many MTR4-CTCF overlapping sites were located at promoters. Stratification of promoters based on HP1 occupancy revealed that, in Het32 cells, CTCF and MTR4 were co-enriched at HP1-bound promoters. Upon HP1 depletion, both MTR4 and CTCF enrichment decreased at these sites, while P-RNAPII signal increased (Fig. 4e). Together, these data indicate that HP1 contributes to the enrichment of MTR4 at a subset of promoters marked by CTCF, rather than globally altering CTCF binding.

### A gain in chromatin accessibility at enhancers affects collagen gene expression

**Fig. 5:**
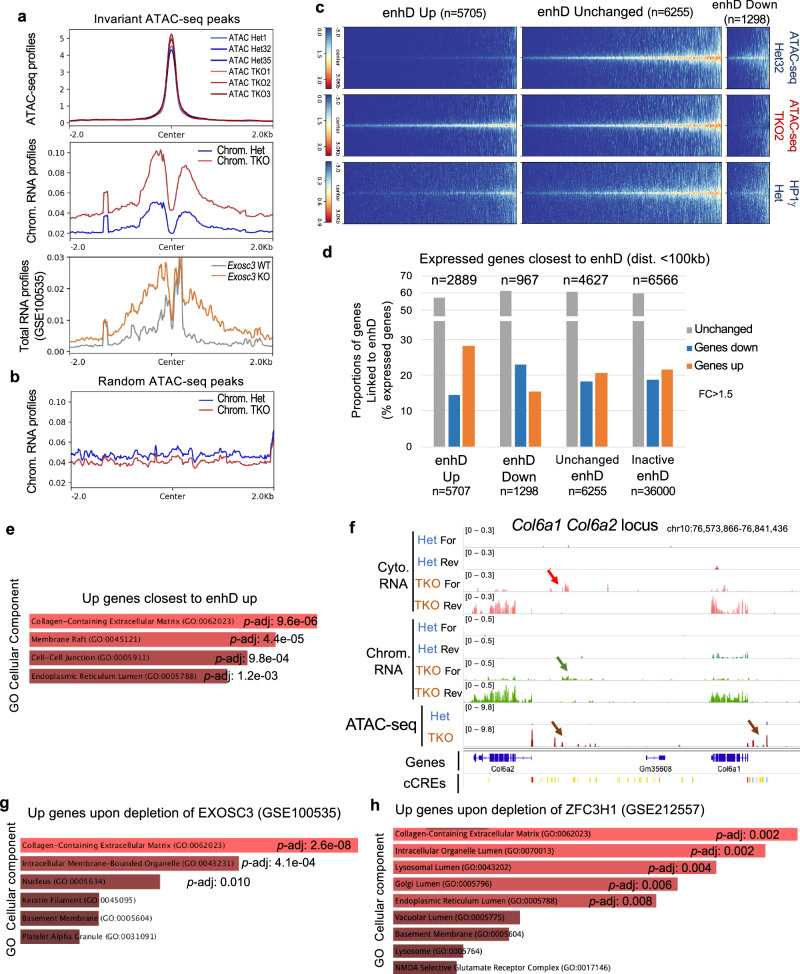
Changes in chromatin accessibility at enhancers affect collagen gene expression. **a**, **b** Profiles centered on extragenic ATAC-seq peaks displaying detectable RNA densities that were invariant between Het and TKO (a, top panel). These peaks show higher levels of chromatin-enriched RNA in TKO compared to Het cells (**a**, middle panel) or compared to the list of ATAC peaks with randomized locations (**b**). RNA is also stabilized in *Exosc3* KO compared to WT control ESCs (**a**, bottom panel, GSE100535 dataset^48^). **c** Heatmaps of ATAC-seq densities in Het and TKO cells, and of HP1γ CUT&Tag density in Het cells centered at cCRE enhD enhancer regulatory elements. EnhD were categorized based on their chromatin accessibility by ATAC-seq between Het and TKO. Categories are as follows: Down, with decreased accessibility in TKO cells; Unchanged, active in both conditions; Up, with increased accessibility in TKO cells; inactive (not shown), with no ATAC-seq density, respectively. **d** Proportions of expressed genes in the closest proximity (within 100 kb) to enhD in the categories depicted in 5b. Bars represent genes that were up- (orange) or down-regulated (blue) by more than 1.5-fold in TKO versus Het, or unchanged (grey). The total number of genes associated with each enhD category is indicated above the histogram. **e** GO term analysis by enrichR associated with upregulated genes closest to upregulated enhD. **f** Genome view, as in Fig. 2a, of stranded chromatin-enriched (green) and cytosolic (red) RNA densities on the *Col6a1 Col6a2* locus, together with ATAC-seq densities in Het and TKO, showing TKO-specific ATAC-seq density (brown arrows) colocalizing with TKO-specific RNA (green and red arrows) and a group of enhD elements depicted in the cCRE lane. Yellow bars, enhD/enhP; red bars, Prom; blue bar, CTCF, as annotated in Supplementary Fig. 8a. **g**, **h** GO terms associated with upregulated genes upon depletion of the indicated RNA exosome components, analyzed with enrichR. *p*-adj, significance of the pathway analyses (Fisher’s exact test, adjusted for multiple hypothesis by the Benjamini-Hochberg procedure). Only *p*-adj <0.05 are indicated. Source data are provided as a Source Data file.

To further investigate the consequences of HP1 loss on regulatory elements in BMEL cells, we performed ATAC-seq in Het and TKO clones. Under the reasonable assumption that stable ATAC-seq signal reflects preserved accessibility of regulatory elements, we first identified a large set of ATAC-seq peaks that remained unchanged upon HP1 inactivation. Among these sites, we focused on intergenic regions to increase the likelihood of detecting RNAs initiated within the ATAC-seq peaks (Fig. 5a, top panel). Notably, these same sites displayed increased accumulation of unstable RNAs upon depletion of EXOSC3 in the publicly available RNA-seq data from mouse embryonic stem cells (ref. ^48^; bottom panel Fig. 5a, brown profiles), indicating that they are normally subject to nuclear RNA exosome–mediated surveillance. In the Het cells, the RNA profiles were compatible with divergent transcription, displaying the characteristic pattern of enhancer-derived eRNA (middle panels Fig. 5a and see ref. ^50^). In TKO cells, this profile was maintained but showed increased intensity despite unchanged ATAC-seq signal (top and middle panels Fig. 5a, b). In addition, many of the loci were enriched for MTR4 and EXOSC10 (Supplementary Fig. 8a). These observations further support the existence of an RNA turnover component contributing to the accumulation of regulatory transcripts upon HP1 depletion.

We next used ATAC-seq to focus on regulatory elements annotated in the ENCODE catalog of candidate cis-regulatory elements (cCREs), and more specifically on distal enhancers (enhD), which frequently overlapped HP1 CUT&Tag peaks (Supplementary Fig. 8b). Based on chromatin accessibility profiles, distal enhancers (enhD) were categorized as active in both conditions, with decreased activity in TKO cells, with increased activity in TKO cells, or inactive (Unchanged, Down, Up, Inactive, respectively. Figure 5b). Heatmap analyses revealed that, similar to observations at promoters, a substantial fraction of active enhancers was associated with HP1 in Het cells (Fig. 5b). Furthermore, Up-regulated enhD showed a clear accumulation of RNA correlated with their increased accessibility by ATAC-seq (Supplementary Fig. 8c), suggesting that enhD elements are subject to nuclear RNA exosome–mediated surveillance.

Importantly, increased ATAC-seq signal at enhancers following HP1 inactivation correlated with the upregulation of nearby genes located within 100 kb (enhD Up, orange bar, Fig. 5c). Gene ontology analysis of these upregulated genes revealed a strong enrichment for terms associated with the collagen-containing extracellular matrix (Fig. 5d and list of affected collagen genes in Supplementary Fig. 9b).

Inspection of the RNA-seq data in a genome browser showed that this activation correlated with an increased accumulation of transcripts over the enhD elements in the chromatin fraction, and at some loci, also in the cytosolic fraction, suggesting increased stability of enhancer RNAs (eRNAs)—potentially a consequence of the reduced recruitment of RNA exosome and its cofactors to chromatin (see Fig. 5e for the *Col6a* locus, and Supplementary Fig. 9a for additional loci). Accumulation of regulatory eRNAs at the Col1a1 locus was validated by RT-qPCR (Supplementary Fig. 9c). Finally, we noted that several collagen gene loci harbored clear MTR4 peaks in their vicinity (Supplementary Fig. 9a).

These findings suggested that collagen genes may be particularly sensitive to decreased turnover of regulatory RNAs. To further support this hypothesis, we analyzed the publicly available RNA-seq data from mouse embryonic stem cells depleted of EXOSC3 (ref. ^48^, Fig. 5f), and another data set from HeLa cells depleted of ZFC3H1 (ref. ^51^, Fig. 5g). Remarkably, in both models, genes associated with the collagen-containing extracellular matrix were among the most significantly affected pathways following disruption of the RNA exosome pathway. Consistent with our TKO RNA-seq data, accumulation of regulatory RNAs at collagen loci was also detectable in the *Exosc3* knockdown dataset at multiple collagen genes (Supplementary Fig. 9a). siRNA-mediated depletion followed by RT–qPCR further confirmed significantly increased regulatory RNA levels at the *Col1a1* locus upon depletion of EXOSC9, EXOSC10, MTR4, or ZC3H18 (Supplementary Fig. 9d).

### HP1 deficiency and reduced activity of RNA exosome and cofactors converge to deregulate collagen genes in hepatocellular carcinoma

To investigate the relevance of HP1–RNA exosome cooperation in hepatocellular carcinoma (HCC), we reanalyzed RNA-sequencing data from a cohort of 76 Mongolian HCC patients^52^. The molecular subtypes identified in this cohort largely mirrored those previously described in patients from other geographic regions, including Asia, Europe, and North America. Our analysis revealed that HP1 gene expression, including *CBX3*, *CBX1*, and *CBX5*, was upregulated in the majority of tumors, with 71% of cases showing at least a twofold increase relative to adjacent healthy tissues (Supplementary Fig. 10a). This observation is consistent with previous reports linking elevated HP1 levels to poor prognosis in HCC (Supplementary Fig. 10b).

**Fig. 6:**
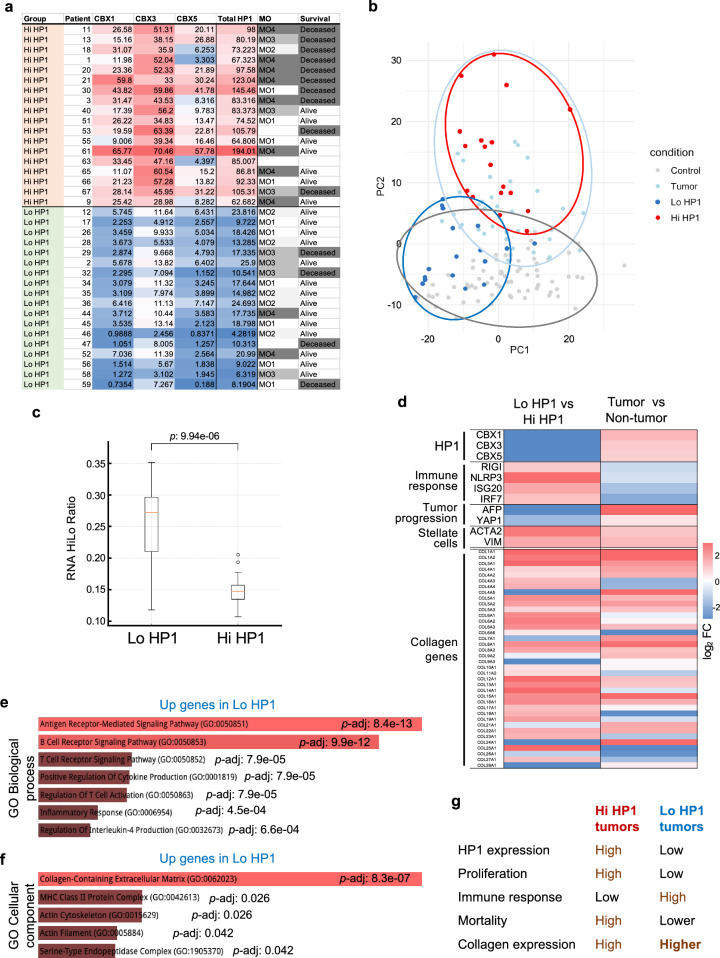
HP1 expression affects collagen genes in hepatocellular carcinoma (HCC) patient samples. **a** Table of patient ranking in high HP1 (Hi HP1) and Low HP1 (Lo HP1) expressing samples based on their cumulative total HP1 expression levels, and compared with gene expression of individual HP1 isoforms. Molecular subclasses (MO) sorted by increasing severity of the disease from MO1 to MO4, and patient survival criteria have been analyzed in ref. ^52^. **b** Unsupervised PCA analysis of patient samples. **c** Quantification of the accumulation of unstable mRNAs in Lo HP1 and Hi HP1 groups using the RNA HiLo ratio analysis as in ref. ^53^. Boxplots show median (central line), IQR (25th and 75th percentiles, box limits); whiskers extend to 1.5 x IQR.; points are values of outliers. *p* value is by unpaired two-tailed Student’s t-test. **d** Heatmaps of log2 fold changes (FC) in expression of genes representative of tumor properties indicated as categories on the left and described in the main text, in Lo HP1 versus Hi HP1 tumor samples (left) and in tumor versus matching non-tumor samples (right). Log2 FC were obtained by DESeq2 analysis. **e**, **f** Histograms of the most significant Gene Ontology (GO) terms for upregulated genes in Lo HP1 patient samples, obtained with enrichR. *p*-adj, significance of the pathway analyses by Fisher’s exact test, adjusted for multiple hypothesis by the Benjamini-Hochberg procedure. **g** Summary of tumor characteristics in the Hi and Lo HP1 groups. Source data are provided as a Source Data file.

To examine the consequences of differential HP1 expression, we ranked patients based on cumulative HP1 levels (sum of *CBX3*, *CBX1*, and *CBX5* expression) and stratified them into Hi HP1 and Lo HP1 groups, corresponding to the upper and lower quartiles (Fig. 6a; Supplementary Fig. 10c). This stratification effectively separated the cohort into two subgroups in unsupervised PCA (Fig. 6b). The Hi HP1 group was enriched for patients previously associated with poor prognosis and included a higher proportion of deceased individuals (Fig. 6a; and Supplementary Fig. 10d, e; ref. ^52^). Genes upregulated in Hi HP1 tumors were significantly enriched for cell cycle–related pathways (adjusted *p* value = 4.8e-14; Supplementary Fig. 10f). These tumors also showed increased expression of the alpha-fetoprotein gene *AFP* and *YAP1*, markers of poorly differentiated and highly proliferative hepatocytes (Fig. 6d, Lo HP1 vs Hi HP1). Together, these data suggest that Hi HP1 tumors are more aggressive, with enhanced proliferation and loss of hepatocytic identity.

To estimate RNA turnover in the Lo HP1 group, we quantified the accumulation of unstable mRNAs using the RNA HiLo ratio, a validated proxy for RNA degradation activity^53^. This approach was used because the available transcriptomes lacked sufficient depth to detect rare transcripts from enhancers or promoters directly. The analysis revealed that labile mRNA species accumulated significantly more in Lo HP1 tumors compared to Hi HP1 tumors, consistent with reduced RNA degradation (Fig. 6c). This accumulation correlated with increased expression of antiviral response genes, including *RIG-I*, *NLRP3*, *ISG20*, and *IRF7* (Fig. 6d, Lo HP1 vs Hi HP1), suggesting activation of an RNA-triggered innate immune response. Supporting this, we observed elevated expression of genes predominantly expressed in immune cells rather than hepatocytes, indicating that the innate immune activation is accompanied by inflammation in the tumor microenvironment.

In parallel, GO cellular component analysis identified “collagen-containing extracellular matrix” as the top enriched category (Fig. 6f). This is consistent with our findings in mouse liver cells with reduced RNA exosome and cofactors activity (Fig. 5d). In Lo HP1 tumors, we also observed increased expression of α-SMA/*ACTA2* and vimentin, markers of activated hepatic stellate cells (HSCs) (Fig. 6d). These changes suggest an increased fibrogenic state, in which HSCs secrete extracellular matrix proteins and drive fibrotic remodeling of the tumor microenvironment.

An increase in extracellular matrix (ECM) stiffness is a characteristic of tumoral progression of HCC, where increased ECM prevents immune infiltration in the tumor, allowing HCC to reach more severe stages^54^. Indeed, tumor samples compared to their matching non-tumor controls revealed typical high levels of collagen gene expression and low immune response (Fig. 6d, tumor vs. non-tumor). Moreover, our findings reveal that Lo HP1 tumors promote both a fibrotic evolution and an anti-viral innate immune response affecting the tumor microenvironment (Summarized in Fig. 6g). Although these data do not establish a direct causal link between HP1 expression and HCC progression, the observed immune response may possibly be triggered by the accumulation of undegraded RNA, that thereby participate in the better survival of Lo HP1 HCC patients. In parallel, the increased expression of collagen-associated genes may be a direct consequence of reduced HP1/RNA exosome and cofactors activity on chromatin, suggesting that these loci in human as in the mouse are particularly sensitive to enhancer regulation coupled to RNA degradation mechanisms.

## Discussion

We identified an unrecognized role for chromatin-associated HP1 proteins in guiding the nuclear RNA exosome to specific genomic loci. HP1 depletion in BMEL cells led to the accumulation of normally unstable nuclear RNAs, including transcripts from repetitive elements, promoters, and enhancers. The increased accumulation of eRNAs was also accompanied by the activation of a subset of enhancers and the subsequent upregulation of nearby genes, many of which were involved in the extracellular matrix, including several collagen genes. This had important functional consequences in the context of hepatocellular carcinoma.

The RNA profile of HP1-depleted cells recapitulated the effects observed upon depletion of nuclear RNA exosome components. PROMPTs and eRNAs were stabilized both in our HP1-deficient cells and in cells lacking core exosome (EXOSC3), NEXT (ZCCHC8), or PAXT (ZFC3H1) subunits, as reported in previous studies^35,55^. In addition, ChIP-seq analysis revealed a dramatic reduction in the number of exosome-binding peaks across the genome in TKO cells, indicating a failure in local recruitment. This suggested that, in addition to the well-characterized role of HP1 proteins in transcriptional repression, these proteins cooperated with NEXT and/or PAXT to promote exosome-mediated RNA decay by directing exosome components to specific chromatin loci. In this context, we noted that HP1 loss did not displace the RNA exosome from chromatin globally, nor did it affect RNA exosome subunit expression, supporting a direct role in targeting rather than chromatin association or complex stability.

The LTR-derived transcripts specifically detected in HP1 triple knockout (TKO) cells point to a dysregulation of RNA exosome activity. LTRs, derived from transposable elements (TEs), are typically embedded in heterochromatin and remain transcriptionally silent in most mammalian cells. This silencing is maintained through several mechanisms: DNA methylation predominates in somatic tissues and late embryonic stages, while in embryonic stem cells, H3K9me3-dependent pathways play a major role, particularly in repressing younger, more transcriptionally active retroelements^56^. Parallels can be drawn from other systems. In *S. pombe*, heterochromatic silencing involves two HP1 homologs: Chp2 represses transcription via the SHREC histone deacetylase complex, while Swi6 binds centromeric non-coding RNAs and promotes their degradation via the RNAi pathway or the RNA exosome^23,25^. In mESC, the human silencing hub (HUSH) complex contributes to TE silencing by being recruited to H3K9me3-marked chromatin through its MPP8 subunit. HUSH also cooperates with RNA degradation pathways, including the NEXT complex, to repress TEs^40,57^. Although we cannot fully exclude a loss of transcriptional repression in HP1-depleted BMEL cells, our data suggest that impaired RNA degradation is the dominant factor. Specifically, the LTR loci showing increased transcript accumulation in TKO cells did not overlap with HUSH-regulated loci identified by MPP8 ChIP-seq in mESCs (Supplementary Fig. 7c, d), making HUSH involvement in this context unlikely. However, other silencing factors, such as TNRC18—an H3K9me3 reader implicated in silencing of young ERV loci^58^—could still contribute. Hence, despite the broad distribution of HP1 proteins throughout chromatin, their context-dependent, discrete association with distinct complexes may provide selectivity in their regulatory activities.

Overall, the increased LTR-derived RNA levels observed in TKO cells likely reflect a combined effect: the loss of HP1-mediated transcriptional repression and defective RNA degradation. This dual mechanism would resemble what has been described in yeast (SHREC and RNAi/exosome) and mESCs (HUSH and NEXT). This hypothesis awaits further investigation.

Although endogenous retroviruses have lost their ability to transpose in mammalian genomes, they still retain many transcriptional regulatory elements due to their viral origin. These elements can serve as platforms for transcription factor binding and initiation, thereby influencing various gene regulatory mechanisms^59^. Over time, many of their LTRs have been co-opted as alternative promoters, enhancers, or even chromatin insulators, such as at CTCF binding sites. In our previous work, we identified several examples of LTR exaptation as enhancers in BMEL cells^45^, and we have also shown that increased transcription of ERVs in multiple sclerosis may reflect reactivation of embryonic enhancers of retroviral origin^60^. In the current study, we found that a relatively small subset of LTR elements gave rise to stabilized transcripts in HP1-deficient (TKO) cells. This suggests that HP1 proteins do not broadly repress LTRs, but instead act on transcriptionally active LTRs, particularly those with enhancer potential, much like the RNAi machinery, which targets loci based on RNA production. In our data, RNAs accumulating from enhancers and transcriptionally active LTRs were associated with increased transcription of nearby genes. While it remains debated whether eRNAs act through their transcription or as functional RNA molecules, they have been proposed to promote chromatin accessibility and modulate the recruitment of transcription factors and elongation regulators^61^. Their accumulation in HP1-depleted cells may thus contribute to the observed increase in RNAPII recruitment/phosphorylation, possibly by sequestering the negative elongation factor NELF and promoting the activation of P-TEFb^62^.

Interestingly, genes involved in collagen and extracellular matrix formation were particularly sensitive to reduced RNA exosome activity. Both HP1 depletion and impaired exosome function were associated with their upregulation, and these genes also showed increased activity in HCC patients with low HP1 expression. The basis for this specificity remains unclear, but certain eRNAs and long non-coding RNAs (lncRNAs) are especially prone to exosome-mediated degradation and accumulate when the exosome is inactivated^63^. It is possible that enhancers regulating extracellular matrix genes belong to this susceptible class. From a disease perspective, overexpression of both HP1 and EXOSC components has been associated with poor prognosis in cancers such as HCC (Supplementary Fig. 6b and ref. ^64^). Paradoxically, complete HP1 loss promotes liver tumorigenesis in aged mice^46^. Because our analysis captures long-term consequences of HP1 depletion rather than the immediate effects of acute loss, we hypothesize that HP1 depletion leads to RNA accumulation, triggering antiviral immune responses. While this response may offer short-term protection and reduce initial tumor severity, its chronic activation could promote inflammation and tumor progression over time, particularly in the aging liver.

## Methods

### Cell lines

Het and TKO cells (Het1, Het32, Het35, TKO1, TKO2, TKO3), as described in Fig. 1a, were independent clones of BMEL cells (males) derived from mouse embryonic liver^46^. The cells were cultured in RPMI 1640 medium (Invitrogen) supplemented with 10% fetal bovine serum (FBS), 1% penicillin-streptomycin, 10 µg/mL insulin, 30 ng/mL IGF-II, and 50 ng/mL EGF, at 37 °C under 5% CO_2_.

### Cell fractionation and total cell extracts

Cells (~10⁶ from a confluent 100 mm plate) were washed twice with ice-cold PBS directly on the plate then incubated on ice for 8 min in 10 mL of swelling buffer (SW): 10 mM Tris-HCl pH 7.5, 2 mM MgCl_2_, 3 mM CaCl_2_, supplemented before use with 1x antiprotease (Roche), 0.5 mM Na3VO4, 20 mM beta-glycerophosphate, 0.1 mM DTT, 10U/ml RNasin (Promega). Cells were removed from the plate by scraping, and pelleted by centrifugation at 400 × *g* for 5 min at 4 °C. Cell pellets were resuspended in 400 µL of lysis buffer (SW buffer containing 10% glycerol and 0.1% NP-40) and gently pipetted up and down 15 times using a P1000 tip and centrifuged at 1200 × *g* for 5 min at 4 °C. The supernatant was considered as the cytosolic fraction. Nuclear pellets were resuspended in 400 µL of RB buffer containing 9 mM EDTA, 0.2 mM EGTA, 0.1% Triton X-100, supplemented before use with 1x antiprotease (Roche), 0.5 mM Na_3_VO_4_, 20 mM beta-glycerophosphate, 1 mM DTT, 10U/ml RNasin (Promega) incubated for 15 min while rotating at 4 °C, and centrifuged at 3500 × *g* for 5 min to pellet chromatin. The supernatant was considered as the nucleosolic fraction. Chromatin pellets were resuspended in 400 µL of RB buffer supplemented with 1% SDS and solubilized by sonication using a water bath sonicator (Diagenode) at high amplitude (6 cycles of 20 s). In parallel, total cell extracts were prepared in RIPA buffer: 150 mM NaCl, 50 mM Tris pH 8, 0.25% sodium deoxycholate, 0.1% SDS, 1% NP-40, freshly supplemented with 0.5 mM DTT and 1× protease inhibitor, RNasin then incubated for 10 min on ice.

### Co-immunoprecipitation assays

Approximately 10⁶ cells from a confluent 100 mm plate were used to prepare nuclei according to the cell fractionation protocol described above. Nuclear pellets were resuspended in 0.5 ml of RIP buffer: 25 mM TRIS pH 7.4, 150 mM KCl, 2.5% glycerol, 0.3% NP-40 freshly supplemented with 1x antiprotease, 0.5 mM Na_3_VO_4_, 20 mM beta-glycerophosphate, 0.1 mM DTT, 10U/ml RNasin, and treated with 7 U/mL TURBO DNase. The suspension was incubated at 37 °C for 5 min. Samples were sheared by three cycles of sonication (10 s ON, 10 s OFF) using a Bioruptor (Diagenode), followed by a second incubation at 37 °C for 5 min. The reaction was stopped by addition of 5 mM EDTA. Samples were then centrifuged at 18,000 × *g* for 10 min at 4 °C. The resulting supernatant, referred to as “RIP extract”, was used for subsequent immunoprecipitation (IP) experiments, and a portion of the RIP extract was reserved as input samples. For coIP performed in the presence of RNase, RNasin was omitted in the RIP buffer and RIP extracts were supplemented with 2ug/mL RNase A ( + RNase) or with RNasin (-RNase).

For each IP, 150 µL of RIP extract was combined with 3 µg of antibodies and 350 µL of RIP buffer. The mixture was incubated for 1h30 at 4 °C on a rotating wheel. Saturated magnetic beads (Dynabeads M-280 sheep anti-rabbit IgG or Dynabeads Protein G, Invitrogen) were prepared by blocking 80 µL of beads per IP in PBS supplemented with 0.1% bovine serum albumin (BSA), 0.5% nonidet-P40 (NP40), and 0.1% polyvinylpyrrolidone (PVP) for 30 min at 4 °C, followed by two washes with Wash V buffer: 0.1% NP-40, 150 mM NaCl, 20 mM TRIS.Cl pH8, 1 mM EDTA. The saturated beads were resuspended in 300 µL of Wash V buffer per IP, and added to the IP mixture. After 45 min of incubation, beads were then washed twice with RIP buffer, twice with Wash V buffer, and once with TE/NP40 buffer: 10 mM TRIS pH 7.4, 1 mM EDTA, 0.01 % NP-40. Beads were eluted in Laemmli sample buffer. Both eluates and input samples were further processed for Western blot analysis.

### Cell fractionation for RNA preparation and sequencing (RNA-seq)

For total RNA extraction, cells were trypsinized, and the resulting pellet was washed with PBS, and pelleted by centrifugation at 1800 rpm for 4 min at 4 °C. The cell pellet was resuspended in 1600 µL of Buffer D: 4 M guanidinium thiocyanate, 25 mM sodium citrate, pH 7.0, 0.5% (wt/vol) N-laurosylsarcosine and 0.1 M 2-mercaptoethanol^65^, homogenized thoroughly for total cell extracts, from which the total RNA was extracted.

For chromatin-associated and cytosolic RNA extraction, cells were resuspended, as described above for cell fractionation, in 125 µL of ice-cold SW buffer and incubated on ice for 8 min. After swelling, 125 µL of lysis buffer was added, and the suspension was gently pipetted 15 times before centrifugation at 1200 × *g* for 4 min at 4 °C. The nuclei pellet was resuspended in 250 µL of Buffer RB and incubated on ice for 15 min, followed by centrifugation at 3500 × *g* for 4 min at 4 °C, separating the chromatin and nucleosol. Both the nucleosol and chromatin fractions were homogenized in Buffer D for RNA extraction.

RNA was extracted by addition to all fractions of 250 mM sodium acetate, 1 volume of acidic phenol, and 0.2 volumes of chloroform. The samples were incubated on ice for 10 min, followed by centrifugation at 18,000 × *g* for 20 min at 4 °C. The supernatant was collected and mixed with an equal volume of chloroform, then centrifuged at 18,000 × *g* for 5 min at 4 °C. The resulting supernatant was combined with an equal volume of isopropanol, vortexed thoroughly, and incubated overnight at −20 °C for RNA precipitation. The samples were centrifuged at 18,000 × *g* for 20 min at 4 °C, and the resulting pellet was washed with 75% ethanol, and resuspended in 100 µL of Buffer D (diluted 1:10), supplemented with 0.5% Triton X-100, 0.4 M NaCl, and 1 µL of proteinase K. The mixture was incubated at 50 °C with occasional shaking for 3 h. Subsequently, 250 mM sodium acetate, 300 µL of Buffer D, and 400 µL of acidic phenol/chloroform were added to each fraction. The samples were centrifuged at 18,000 × *g* for 15 min at 4 °C, and the supernatant was collected. An equal volume of chloroform was added to the supernatant, followed by centrifugation at 18,000 × *g* for 20 min at 4 °C. The final supernatant was supplemented with glycogen (2 µg/mL) as carrier and 1 volume of isopropanol, and the samples were precipitated overnight at −20 °C. The pellet was then washed with 75% ethanol and resuspended in 50 µL of RNase-free water. The samples were incubated at 45 °C with occasional shaking for 10 min. 40 µL was subjected to DNase treatment for 50 min at 37 °C. RNA integrity was verified by agarose gel electrophoresis. On all RNA fractions, total RNA library preparation and sequencing were performed by Novogene (UK) Co., Ltd as part of their lncRNA-seq service, which included directional library preparation with rRNA removal and sequencing on the Illumina NovaSeq platform using paired-end 150 bp (PE150) reads.

### Chromatin immunoprecipitation and sequencing (ChIP-seq)

BMEL cells were crosslinked following a modification of the protocol described in ref. ^66^. Briefly cells were incubated in freshly prepared 2 mM di-succinimidyl-glutarate (DSG, Merck) in PBS for 50 min at room temperature with occasional shaking. After three washes in PBS, cells were incubated for 10 min in 1% formaldehyde (Merck) in PBS. The crosslinking reaction was quenched with 125 mM glycine for 5 min at room temperature. Cells were washed with PBS, harvested, and pelleted by centrifugation at 1000 × *g* for 3 min. Cells were subsequently resuspended in 1.4 mL of freshly prepared swelling buffer (25 mM Hepes pH 7.95, 10 mM KCl, 10 mM EDTA) supplemented with protease inhibitors (Roche) and 0.5% NP-40. The suspension was incubated on ice for 30 min, then pipetted up and down 50 times using a P1000 pipette tip. The cells were centrifuged at 3500 × *g* for 2 min and resuspended in 300 µL of TSE150 buffer with 0.3 % SDS (0.3% SDS, 1% Triton, 2 mM EDTA, 20 mM Tris-HCl pH8, 150 mM NaCl) freshly supplemented with protease inhibitors. Chromatin was sonicated in 1.5 mL tubes for a total of 15 min using cycles of 30 s ON and 30 s OFF, with the samples maintained in ice-cold water. Following sonication, the samples were centrifuged at 18,000 × *g* for 30 min at 18 °C. An aliquot of supernatant was used to quantify chromatin and check DNA fragment size (typically around 300 bp).

Chromatin immunoprecipitation (ChIP) was performed using 70 µg of chromatin and 4 µg of the following antibodies: anti-MTR4/SKIV2L2 (Bethyl A300-615A), anti-ZC3H18 (Atlas HPA 040847), anti-EXOSC10 (Bethyl A303-987A), anti-CTCF (Diagenode A2354-0023P), anti-P-RNAPII (a mix of antibodies against phospho-Ser5 and phospho-Ser2, Abcam Ab5095 and Ab5408, respectively), and anti-murine IgG (Merck). The EXOSC9 and EXOSC3 subunits were not included since they lacked available ChIP antibodies. Immunoprecipitation was carried out overnight on a rotating wheel at 4 °C. Subsequently, 90 µL of Protein G beads (Dynabeads, Invitrogen), blocked overnight at 4 °C in blocking buffer containing 5% Buffer C (0.5% SDS, 10 mM Tris-Cl pH 8, 1 mM EDTA, 0.5 mM EGTA), 20% Buffer D 1.2× (1.2% Triton X-100, 0.12% NaDoc, 180 mM NaCl, 12 mM Tris-Cl pH 8, 1.2 mM EDTA, 0.6 mM EGTA), 72% Wash Buffer V, 1.5% BSA (Merck), and 1.5% Polyvinylpyrrolidone (Merck), were added to the IP samples and incubated for 1 h on a rotating wheel at 4 °C. Ten percent of the chromatin was set aside as input. Following immunoprecipitation, the beads were pelleted and subjected to sequential washes, each performed for 3 min on a rotating wheel at room temperature in 1 mL of the following buffers: Three washes in TSE150 buffer with 0.1 % SDS (0.1% SDS, 1% Triton, 2 mM EDTA, 20 mM Tris-HCl pH8, 150 mM NaCl), then one wash in TSE500 buffer (TSE150 buffer with 0.1% SDS and 500 mM NaCl), then one wash in buffer (10 mM Tris-HCl pH 8, 0.25 M LiCl, 0.5% NP-40, 0.5% sodium deoxycholate, 1 mM EDTA), then two washes in TE buffer (10 mM Tris-HCl pH 8, 1 mM EDTA). Two successive elutions were performed in 300 µL and then 100 µL of elution buffer (1% SDS, 10 mM EDTA, 50 mM Tris-HCl pH 8) by vortexing vigorously, followed by incubation at 65 °C for 15 min. Beads were washed in 100 µL of elution buffer added to the pooled eluates. Samples were incubated overnight at 65 °C to reverse crosslink, followed by proteinase K and RNase A treatments. DNA was isolated using a NucleoSpin PCR purification kit (Macherey Nagel), and concentrations were measured using a Qubit fluorometer (Invitrogen). Purified ChIP samples were processed for Illumina paired-end (PE40) sequencing performed at the Genom’IC core facility (Paris, France).

### Western Blotting analysis

Protein samples were denatured by the addition of Laemmli sample buffer (Bio-Rad) with reducing agent at 100 °C for 10 min and separated by SDS-PAGE (Bio-Rad, Criterion XT), then transferred to a nitrocellulose membrane. Transferred proteins were visualized by Ponceau S staining, and the membranes were incubated overnight at 4 °C in PBS-T (PBS, 0.1% Tween 20), containing 5% (w/v) non-fat dry milk. The membranes were then incubated for 1h30 at room temperature with primary antibodies at the appropriate dilution in PBS-T (as described in Supplementary Table 1). After three 5-min washes in PBS-T, the membranes were incubated for 1h30 with secondary antibodies (Starbright Blue 700 anti-rabbit/mouse IgG (Biorad), or rabbit true blot (Rockland), all diluted 1/4000), followed by three additional 5-min washes. Protein detection was performed either by fluorescence at 700 nm or by chemiluminescence using the Pierce™ ECL Plus Western Blotting Substrate (Thermo Fisher Scientific) and visualized with a ChemiDoc MP imaging system (Bio-Rad).

### ATAC-seq

Chromatin accessibility assays were performed on each of the six Het and TKO BMEL clones according to the protocol described in ref. ^67^, with an ATAC-seq kit (ActiveMotif, 53150). Paired-end (PE40) sequencing of the dual-index ATAC-seq libraries was performed by Novogene (UK) Co., Ltd.

### CUT&Tag

CUT&Tag assays were performed on two independent biological replicates of Het and TKO BMEL cells according to the protocol by ref. ^68^, but with the NE1 buffer (https://www.protocols.io/view/bench-top-cut-amp-tag-kqdg34qdpl25/v3) to permeabilize cells, and with the same anti-HP1α, -HP1β, -HP1γ, -H3K9me3 as used in Western blot analyses, and with anti-H3K27me3 (Cell Signaling Technol. 9733). Libraries were obtained with 15-16 PCR amplification cycles such that at least a faint ladder of nucleosome-sized bands were visible using an Agilent Tape Station High-Sensitivity D1000 Screen Tape representing DNA concentrations of at least 200 pg/µL for anti-HP1 CUT&Tag reactions with Het cells, and at least 10-fold lower concentrations for the same reactions using TKO cells. 40 nt paired-end sequencing was performed at the I2BC sequencing platform. Reads were aligned on the mm10 reference genome with bowtie2 (parameters: --end-to-end --very-sensitive --no-mixed --no-discordant -k 1 -X 1000 --phred33 -I 25 -p 24 -x mm10). Bigwig files were generated from two merged HP1 CUT&Tag replicates, and they were normalized using CPM.

### Bioinformatics analyses

RNA-seq data analysis: Mapping was carried out with STAR (v2.6.0b) (parameters: –outFilterMismatchNmax 1 – outSAMmultNmax 1 –outMultimapperOrder Random –outFilterMultimapNmax 30). The reference genome was GRCm38 Mus musculus primary assembly from Ensembl. The SAM files were converted to BAM files and sorted by coordinate using samtools (v1.8). Gene expression analysis was performed with the DESeq2 (v1.18.1) package. *P* values from the differential gene expression test were adjusted for multiple testing according to the Benjamini and Hochberg procedure.

ChIP-seq data analysis: Raw ChIP-seq data in Fastq format were subjected to quality control using FastQC (v0.11.9). ChIP-seq reads were mapped to mm10 using bowtie2 (v2.3.4) (parameters: -N 0 -k 1 –very-sensitive-local). We then selected reads with a MAPQ equal or higher than 30 corresponding to uniquely mapped reads for further analysis. Peak calling was performed using MACS2 (v.2.1.1) (parameters: -p 0.05).

ATAC-seq data analysis: For chromatin accessibility profiling, Bowtie2 was used with the parameters --very-sensitive -X 2000 to accommodate the broad range of ATAC-seq fragment sizes, and --maxins 500 --no-discordant --no-mixed -k 1 to eliminate multi-mapping reads. Aligned BAM files were sorted and indexed using SAMtools (v1.8). ATAC-seq peaks were called using MACS2 (v2.2.9.1) with the parameters --nomodel, --shift −100, and --extsize 200, which are recommended for ATAC-seq to account for the Tn5 transposase cutting pattern. Peaks were identified using a q-value threshold of 0.01 (-q 0.01), and signal tracks were generated in bedGraph format with -B --SPMR. The genome size was set to mouse (-g mm), and input files were sorted BAM files in BAM format (-f BAM).

For all high throughput experiments, Bigwig files were generated from BAM files with bamCoverage (parameter: –normalizeUsing CPM) from Deeptools (v3.1.3)

Pathway analyses on Gene expression datasets with Entrez gene names were performed with Enrichr (https://maayanlab.cloud/Enrichr/). Analyses of the gene set from GSE100535 (Fig. 5g) with enrichR was performed against a background list of expressed genes (>100 base mean counts). Bar graphs illustrate ranking based on *p* values. The adjusted *p* values (*p*-adj) of each path entry served as the significance threshold.

Genes adjacent to dysregulated enhD were defined with GREAT (v. 4.0.4) with association rules: “Two nearest genes (100.0 kb max extension).” (http://great.stanford.edu/public/html/). Genes with more than 10 reads were then sorted into up-, down-regulated or unchanged based on their fold change in expression by DESeq2 analysis.

### Definition of genomic regions and categories

LTR, SINE, LINE, and MMSAT regions were defined by the RepClass field in the Repeat masker database in the UCSC table browser (https://genome.ucsc.edu/). Distal enhancers were defined as the dELS/enhD category in the Encyclopedia of DNA Elements (ENCODE) candidate cis-regulatory element (cCRE) database in the UCSC table browser. Extragenic repeats and enhD elements were defined relative to genes in the Ensembl GRCm38 database (https://www.ensembl.org/). Promoter associated transcripts (PROMPT) regions were defined as 2 kb upstream of transcription start sites (TSS) of genes. Exons were defined according to the Ensembl GRCm38 database. The list of 23808 Genes was defined according to the Ensembl GRCm38 database, after removing all the entries with names beginning with “Mir”, “Rik”, and “Gm”, for the sake of clarity of the average density profiles.

Categories of distal enhancer elements (enhD) from ENCODE database were created according to their activity as defined by their chromatin accessibility measured by ATAC-seq read counts by featurecounts among all Het and TKO clones. The EnhD (Up, Down, Unchanged, and Closed) categories are based on their mean ATAC-seq read counts in Het or in TKO, and their fold change (FC) in TKO versus Het as follows: enhD Up (mean >10; FC > = 4; *p*-val <0.05), enhD Down (mean >10; FC = < 0.25; *p*-val <0.05), enhD Unchanged (mean >10; 0.9 < FC < 1.1), enhD Closed (mean <2).

### RNA read counts on genomic regions

Quantification of uniquely mapped reads from the transcriptome was carried out with featureCounts (v1.6.1) from the Subread suite^69^, with the following parameters: -p -B -C -s 1 --minOverlap 25. FeatureCounts with these parameters counted only uniquely mapped reads with both paired-ends aligned (-p -B).

Chromatin and cytoplasmic stranded RNA reads were counted on extragenic repeats only in order to evaluate self-expressed repeats and avoid counting reads on gene-embedded repeats which would reflect expression of the surrounding gene and not the repeat itself. Read counts were the results of stranded reads counted in regions subtracted by stranded reads counted in the 200nt region upstream of repeats adjusted for their size.

Transcriptome reads on PROMPT were counted antisense, corresponding to upstream-antisense RNA (uaRNA), relative to the orientation of their adjacent gene subtracted by antisense read counts in its first exon adjusted for their respective size. Transcriptome read counts on enhD were subtracted by read counts in the highest of the two adjacent 200nt region upstream or downstream of each enhD adjusted for their respective size. All genomic regions counted for RNA levels were considered expressed when two or more reads (or ten or more reads on exons) were counted in average of the triplicate samples, in either Het or TKO or both, after subtracting reads from size-normalized adjacent regions (illustrated in Fig. 2b, e). In order to avoid excluding the elements expressed only in Het or in TKO, a pseudo-count of 0.1 was added to the mean RNA read counts before transformation into Log2 (illustrated in Fig. 2c-d, f-g).

### Data visualization

BigWig files of samples or merged replicates were visualized with the Integrative Genomics Viewer (IGV version 2.8.10). Heatmaps and profiles were generated with the computeMatrix, plotProfile and plotHeatmap tools of the Deeptools package (v3.1.3). The STRING analysis of protein associations with ZC3H18 in Fig. 1a has been downloaded from the STRING database, (https://string-db.org/).

### Statistical analyses

The statistical tests performed are described in Fig. legends or in the “Methods” section. Significance level was evaluated by Student’s t-test as indicated in Figure legends. Statistical significance of the number of colocalized peaks in Fig. 4a was assessed using a rank-based permutation test (*n* = 200 randomizations by shuffle of the genomic locations of the MTR4 ChIP-seq peaks), and empirical *p* values were computed from the null distribution with one-sided upper-tailed test for all colocalizations, except one-sided lower-tailed test for the number of colocalizations with “none”. *p* < 0.05 was considered significant. The significance in pathway analyses performed with enrichR was computed using the Fisher exact test and presented as adjusted *p* values (*p*-adj), adjusted for multiple hypothesis testing with the Benjamini-Hochberg procedure. No statistical method was used to predetermine sample size. No data were excluded from the analyses. The Investigators were not blinded to allocation during experiments and outcome assessment.

### Reporting summary

Further information on research design is available in the Nature Portfolio Reporting Summary linked to this article.

## Supplementary information

Supplementary Information

Reporting Summary

Transparent Peer Review file

## Source data

Source Data

## Data Availability

All RNA-seq, ChIP-seq, ATAC-seq, and CUT&Tag datasets generated during this study have been deposited in the EBI BioStudies database under accession codes: E-MTAB-15194, E-MTAB-15196, E-MTAB-15197, and E-MTAB-15189, respectively. The previously published datasets are accessible under accession codes GSE100535 (*Exosc3* CKO transcriptome), GSE178550 (MPP8 ChIP-seq and *Zcchc8* KO and *Zfc3h1* KO transcriptomes), GSE212557 (*ZFC3H1* knockdown transcriptome), and GSE144269 (HCC transcriptomes). Source data are provided with this paper.

## Materials availability

The Het and TKO BMEL cell lines are readily available from the authors upon request.
