# Supplementary material for: Targeting of the nuclear RNA exosome to chromatin by HP1 affects the transcriptional programs of liver cells
Source: Nat Commun. 2026 Apr 29;17:5865. doi: 10.1038/s41467-026-72504-7 (PMC13333803; doi:10.1038/s41467-026-72504-7)
Supplement: Supplementary file 1 — Supplementary Information [file 41467_2026_72504_MOESM1_ESM.pdf]

# Supplementary information

## Targeting of the nuclear RNA exosome to chromatin by HP1 affects the transcriptional programs of liver cells.

Hiba SOUAIFAN 1,2, Mickael COSTALLAT 1, Laura SITKIEWICZ 3, Kylian GODEST 4, Florence CAMMAS 3, Carl MANN 4, Christian MUCHARDT 1*, Christophe RACHEZ 1*

1. Institut de Biologie Paris-Seine (IBPS), Sorbonne Université, CNRS UMR7238, 75252 Paris, France
2. Ecole doctorale Complexité du Vivant, Sorbonne Université, Paris, France
3. Institute of Human Genetics, CNRS UMR9002 University of Montpellier, 34396 Montpellier, France
4. Institut de Biologie Intégrative de la Cellule (I2BC), CEA, CNRS, Université Paris-Saclay, 91190 Gif-sur-Yvette, France

* Co-corresponding authors: christian.muchardt@sorbonne-universite.fr
christophe.rachez@sorbonne-universite.fr

This file includes:

SUPPLEMENTARY FIGURES 1-10
SUPPLEMENTARY TABLES 1-2
SUPPLEMENTARY METHODS
SUPPLEMENTARY REFERENCES

## Supplementary Fig. 1

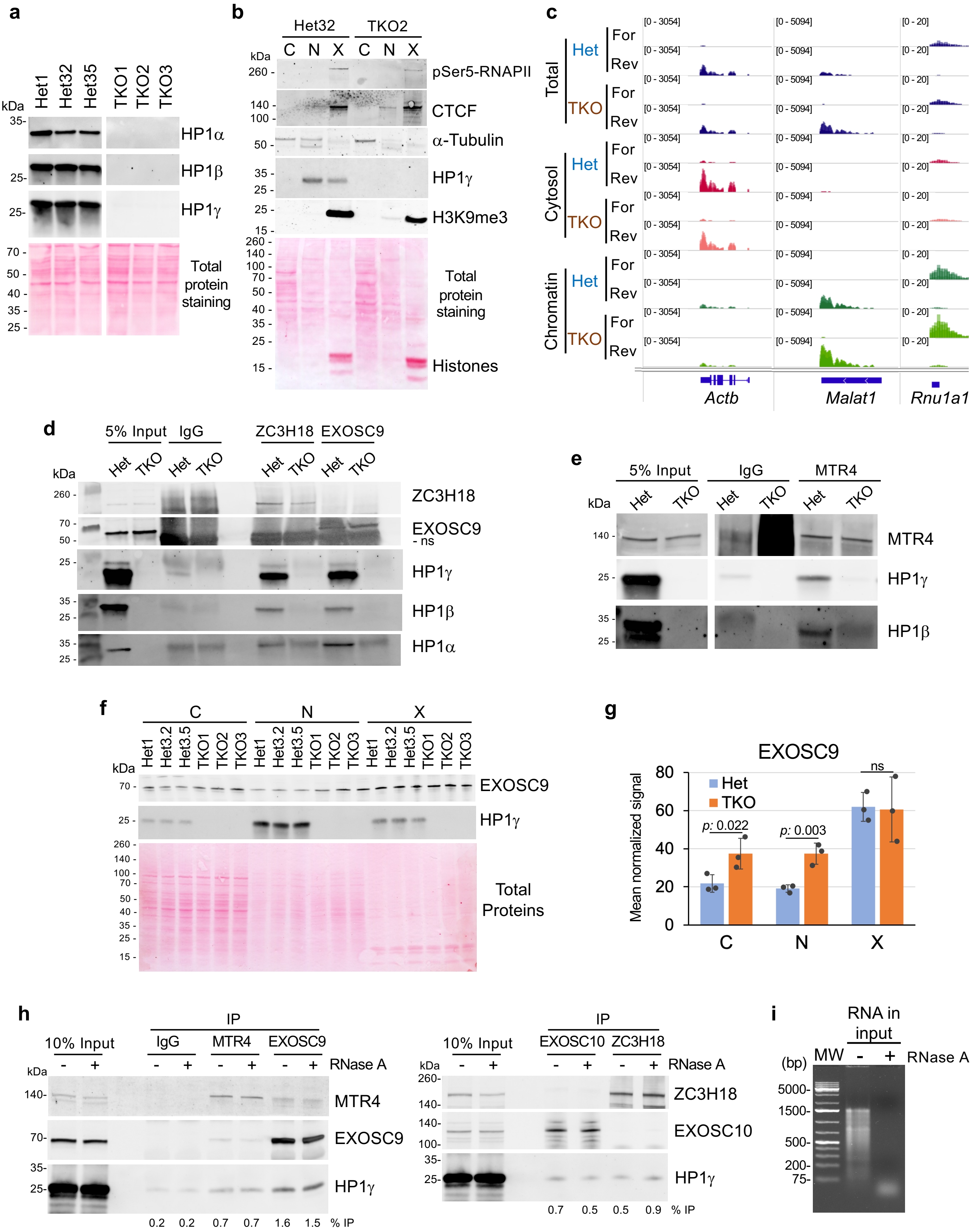

**Supplementary Fig. 1: Cell fractionation and co-immunoprecipitation assays**

**a.** Levels of HP1 proteins in all BMEL clones by Western blot analysis. n=4 independent experiments. **b.** Subcellular Distribution of indicated proteins in the C, N, X fractions depicted in Fig. 1b, in two representative Het and TKO clones, by western blot analysis, and total proteins by Ponceau S staining. n=3 independent experiments. **c.** Genome views as in Figure 2a of the distribution of total, cytosolic, and chromatin-associated RNA densities for *Actb* mRNA and the nuclear *Malat1* RNA or U-snRNA. RNA distribution reflects the expected subcellular localizations of the depicted RNA species. **d, e.** Co-immunoprecipitation assays of HP1α, HP1β, and HP1γ by MTR4, ZC3H18, or EXOSC9, compared to IgG. Blots are representative of 2 independent experiments **f.** Subcellular distribution of EXOSC9 and HP1γ proteins in the C, N, X fractions depicted in Fig. 1b, in each of the six Het and TKO clones, by western blot analysis, and total protein staining by Ponceau S. n=3 independent experiments. **g.** Quantification of EXOSC9 signal in supplementary fig. 1f, graphed as a mean, and SD among the three Het or TKO clones. *p*-values represent significance level by unpaired two-tailed Student's t-test. ns, non-significant. n=3 independent clones. **h.** RNA-independent co-immunoprecipitation of HP1γ by MTR4, EXOSC9, EXOSC10, or ZC3H18. HP1γ signal is not lost upon RNase A treatment. %IP, quantitation of HP1γ IP efficiency versus 10 % input. Blots are representative of 2 independent experiments. **i.** RNA is fully degraded in the RNase A-treated (+) compared to untreated (-) extracts used in the co-IP experiment in panel H, as visualized by agarose gel electrophoresis of RNA purified out of input samples. Source data are provided as a Source Data file.

**Supplementary Fig. 2**

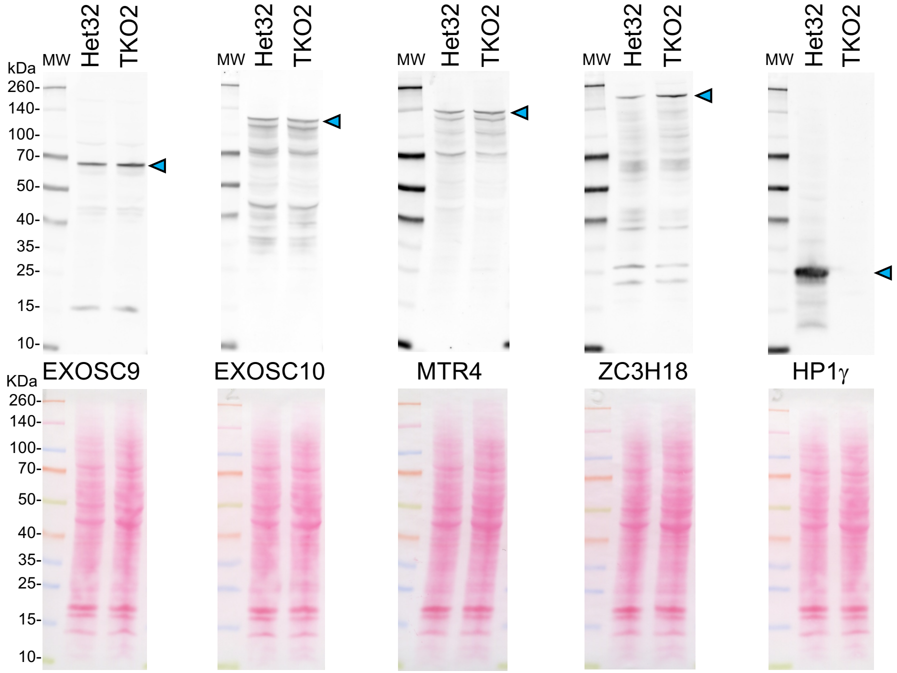


**Supplementary Fig. 2: Specificity of the antibodies used in immunoprecipitation assays.**
The antibodies used in direct IP against exosome-related proteins do not cross-react with HP1 proteins by western blot analysis. Ponceau S-stained membranes are shown below the blots as loading controls. MW, molecular weight protein markers. Blue arrowheads correspond to the predicted specific protein signal. Blots are representative of n=3 independent experiments.

**Supplementary Fig. 3**

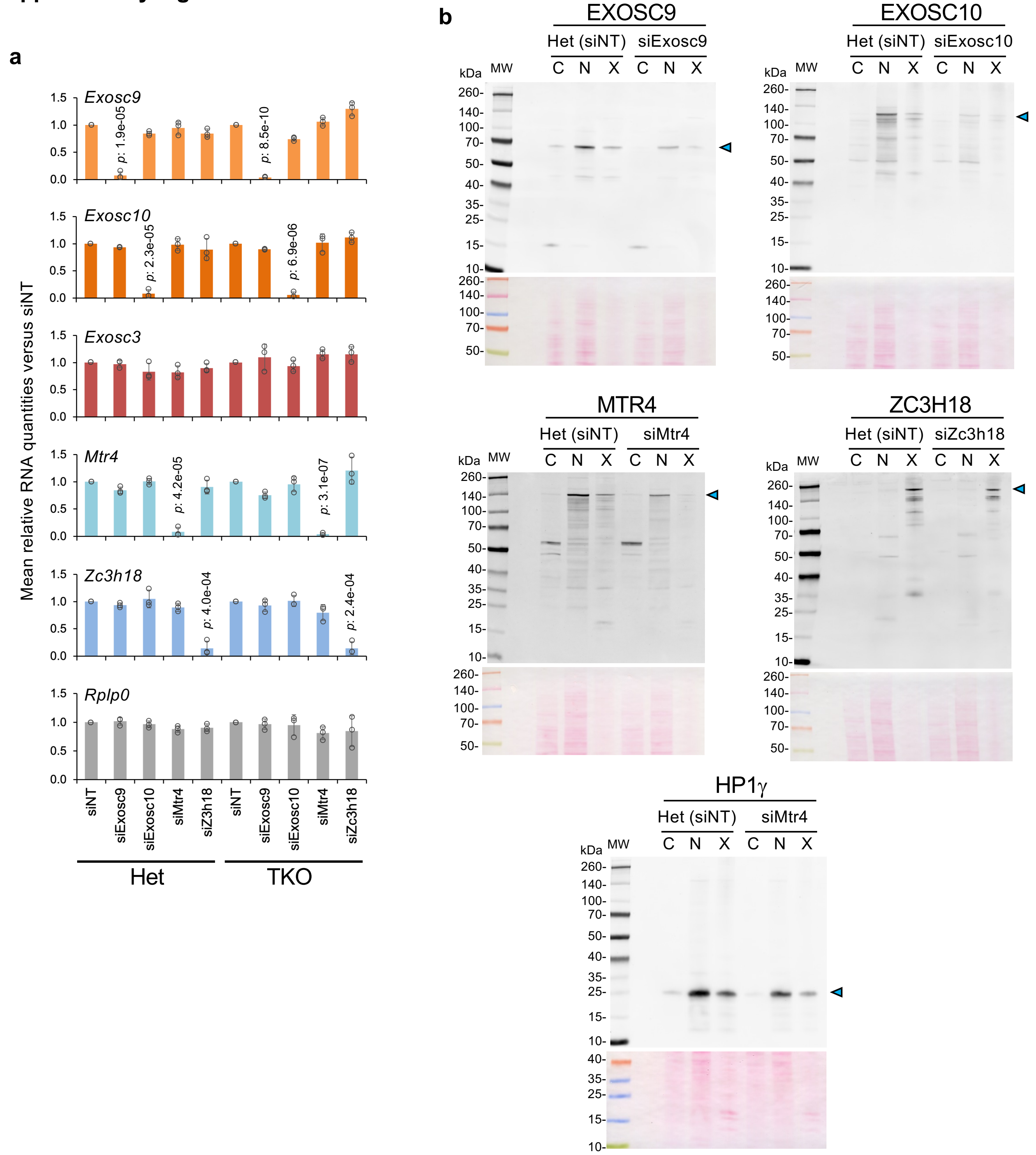


**Supplementary Fig. 3: Characterization of siRNA-mediated knockdown in Het cells.**
**a.** RT-qPCR analysis of RNA levels upon knockdown of the indicated targets by siRNA in Het and in TKO cells, relative to non-targeting siRNA (siNT) knockdown as controls. The non-targeted *Exosc3* and *Rplp0* are included as controls. Data are mean +/-SD. *p*-values represent significance of the differences relative to siNT by two-sided, unpaired Student's t-test. Only *p-values* < 0.0005 are shown. n=3 independent experiments. **b.** Relative targeted protein levels upon knockdown of exosome components by siRNA in Het cells treated with the indicated siRNA or non-targeting (siNT) siRNA. The extend of protein depletion was visualized by western blot in cell fractions as in Fig. 1b. Ponceau S-stained membranes are shown below the blots as loading controls. MW, molecular weight protein markers. Blue arrowheads correspond to the expected specific protein signal. Blots are representative of n=3 independent experiments. Source data are provided as a Source Data file.

## Supplementary Fig. 4

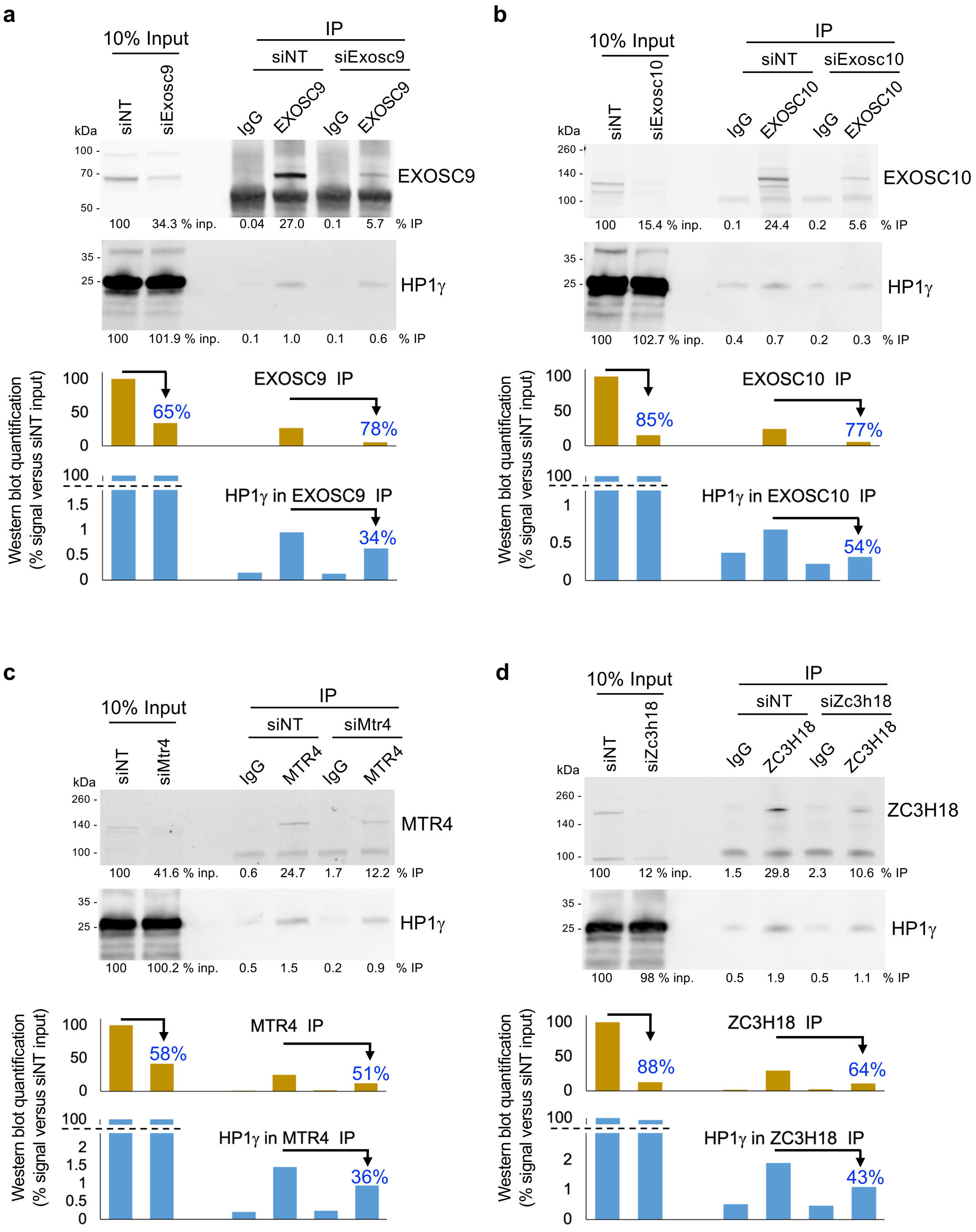

**Supplementary Fig. 4: Specificity of HP1γ co-immunoprecipitation in EXOSC9, EXOSC10, MTR4, and ZC3H18 IP experiments.**

The loss of exosome/cofactor proteins detected in their specific IP experiments following siRNA-mediated knockdown leads to a concomitant loss of HP1γ co-immunoprecipitated by these proteins (blue percentages). Immunoprecipitation of EXOSC9 (**a**), EXOSC10 (**b**), MTR4 (**c**), ZC3H18 (**d**) exosome components/cofactors were performed following individual knockdown by the indicated siRNA, compared to non-targeting siRNA (siNT) knockdown controls. Immunoprecipitations were performed with the depicted antibodies against the respective siRNA-depleted proteins, or with negative control IgG, followed by western blot analysis of either the specific depleted protein or its co-immunoprecipitated interacting HP1γ. The percentages of proteins in immunoprecipitated (% IP) or in input (% inp.) samples were measured versus siNT input by quantification of the specific bands in Western blot normalized to the signal in siNT input lanes corresponding to 100% of input. Quantifications are also illustrated below each blot, as histograms for exosome components/cofactors (brown bars), and for HP1γ (blue bars). The blue numbers indicate the percentage of signal loss in input or IP relative to siNT, in the blots above histograms. Western blots and their quantifications are representative of n=2 (**a** and **d**) or n=3 (**b** and **c**) independent co-immunoprecipitation assays. Source data are provided as a Source Data file.

## Supplementary Fig. 5

**a**

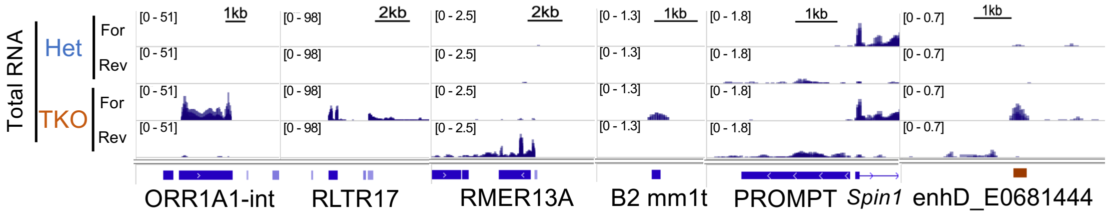


**b**

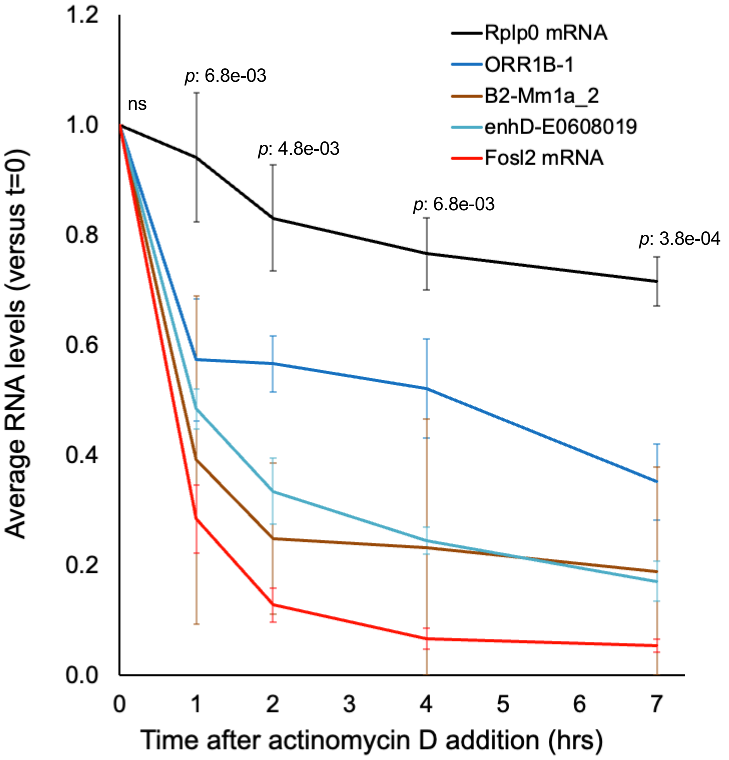


**Supplementary Fig. 5: Transcripts detected at LTR, SINE, and enhancer loci are unstable in BMEL Het cells.**

**a**. Genome views of total RNA density profiles (merged triplicates), related to Fig. 2a. **b.** Time course of RNA stability following Actinomycin D treatment in BMEL Het cells tested for typical examples of transcripts at LTR (ORR1B, Blue), SINE (B2-Mm1a, brown), and enhancer (enhD_E0608019, turquoise) loci, compared to the stable *Rplp0* mRNA (black), and the unstable *Fosl2* mRNA (red). *Rplp0* and *Fosl2* mRNA transcripts are considered stable and unstable, respectively, based on their average half-lives of 15h (*Rplp0*) and 2h (*Fosl2*), calculated previously[1]. Graph represents mean and average deviations. *p*-values represent significance of the differences between *Rplp0* mRNA and ORR1B transcripts by one-sided, unpaired Student's t-test. n=4 biological replicates. Experiment was repeated 3-times with similar results. Source data are provided as a Source Data file.

**Supplementary Fig. 6**

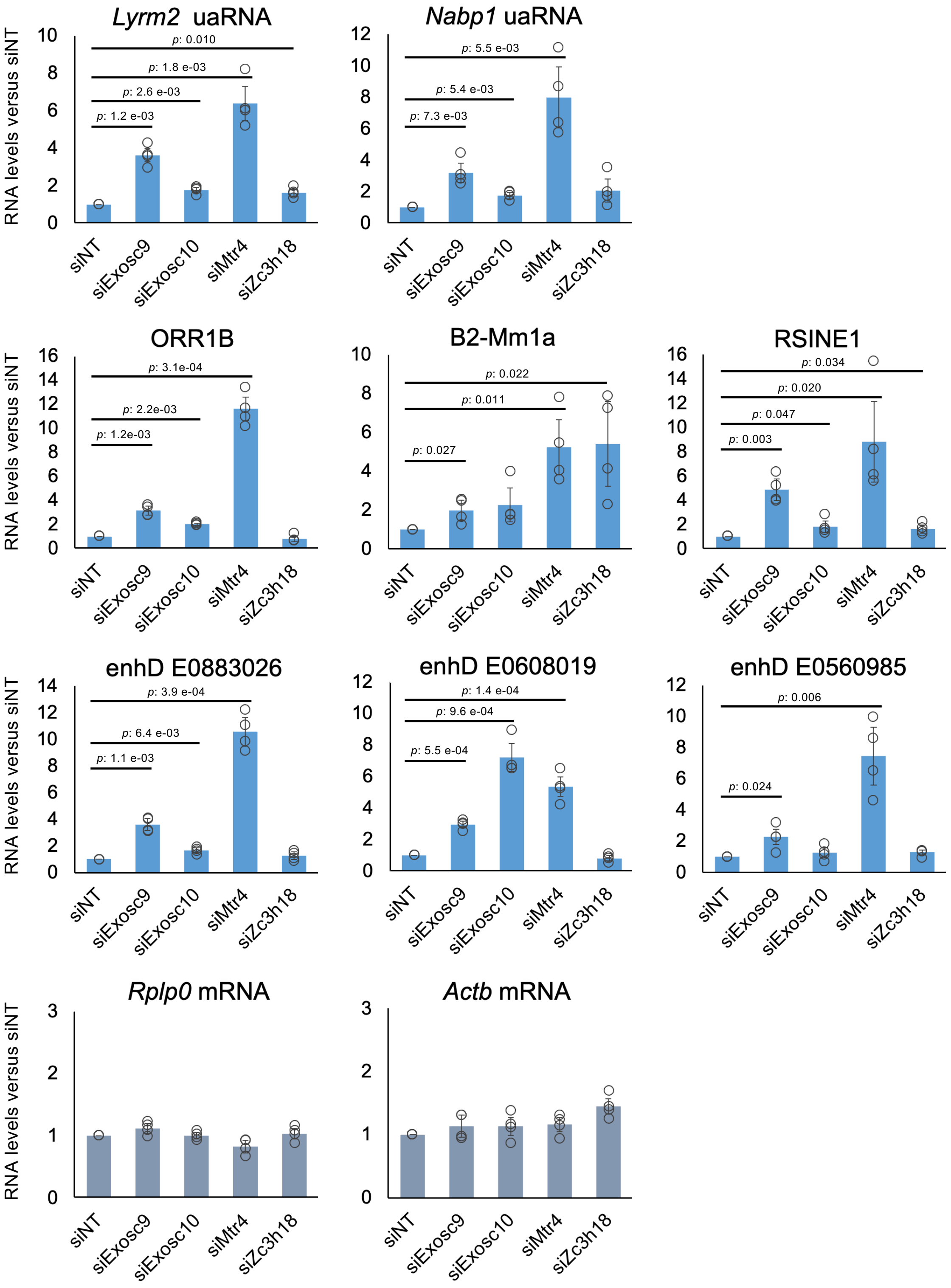


**Supplementary Fig. 6: Knockdown of exosome or its associated nuclear cofactors increases RNA levels at loci that were found upregulated in TKO cells.**

RNA levels at the indicated unstable RNA loci were stabilized by siRNA knockdown of some of the indicated exosome-associated components compared to non-targeting (siNT) controls. Histograms represent mean and average deviation of relative RNA levels quantitated by RT-qPCR versus siNT. *p*-values represent significance of the differences between siRNA knockdown and siNT (one-sided, unpaired Student's t-test). Only the significant differences with $p < 0.05$ are illustrated. n=4 biological replicates. Experiment was repeated with similar results. Source data are provided as a Source Data file.

## Supplementary Fig. 7

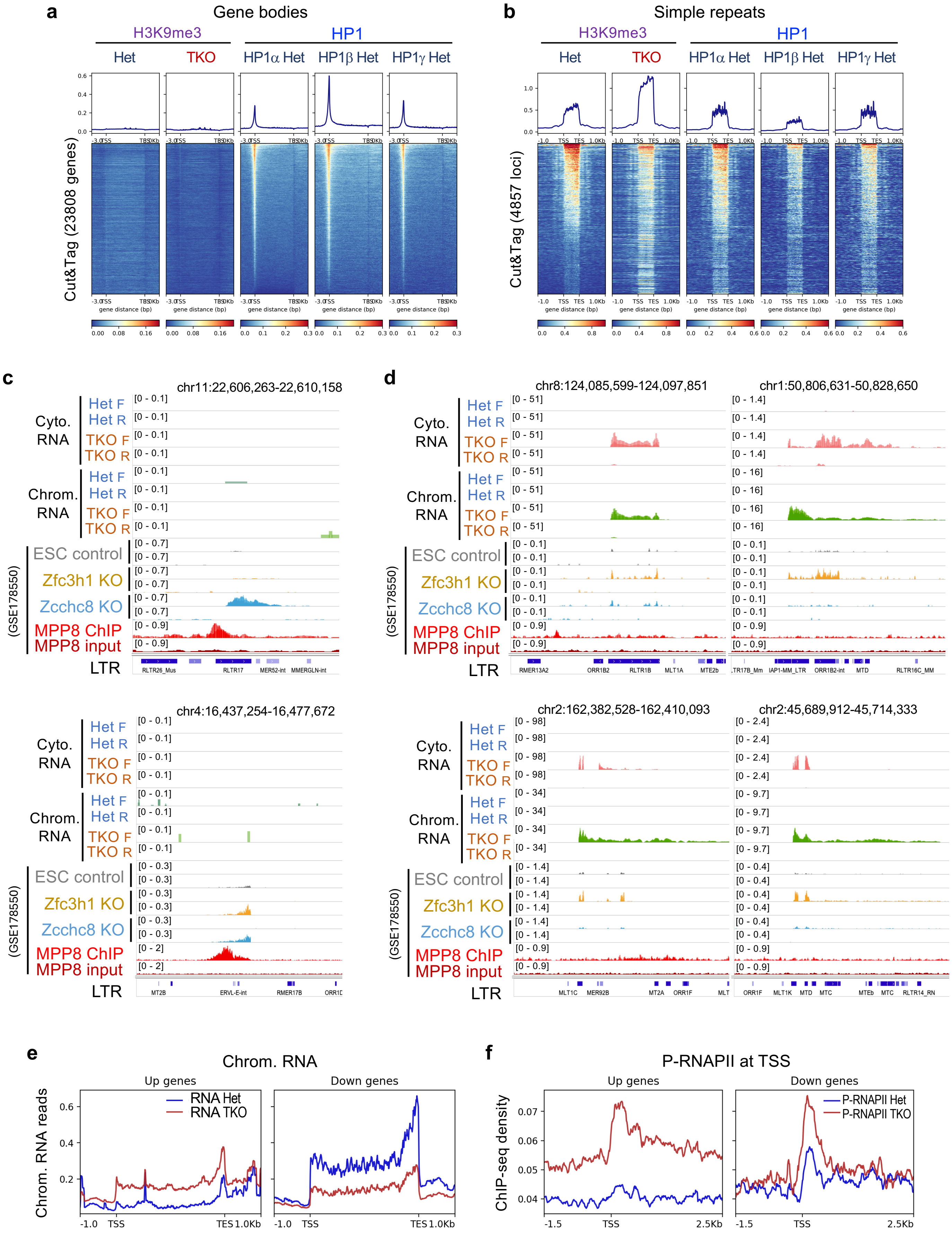

**Supplementary Fig. 7: HP1 binding is not always correlated with H3K9me3; Increased RNA density in TKO is not correlated with MPP8 binding and the HUSH complex; Phospho-RNAPII density is not correlated with chromatin-associated RNA density in TKO versus Het.**

**a, b.** Heatmaps of densities of H3K9me3 or HP1 isoforms by CUT&Tag in Het or TKO as indicated, on gene bodies (**a**) as in figures 3d-f, or on a list of simple repeats (**b**) from Repeat Masker database. **c, d.** Genome views of transcriptomes in Het or TKO (examples depicted in Fig. 2h) compared with transcriptomes of mESC invalidated for the expression of Zcchc8 KO (sky blue profiles) or Zfc3h1 KO (yellow profiles) and control mESC (grey profiles), together with MPP8 ChIP-seq (red) and input (dark red) profiles (GSE178550 datasets[2]). **c.** Genome views of two examples of MPP8 ChIP-seq peaks with a coincidental RNA profile in mESC KO. No upregulated RNA was detectable at these loci in Het or TKO. **d.** Genome views of upregulated RNA (green and red) in TKO at LTR loci, correlated to upregulated RNA in mESC KO (yellow and sky blue). No MPP8 ChIP-seq peak was detectable at these loci. **e, f.** Down-regulated genes in TKO show lower RNA density on chromatin over gene bodies, compared to Up-regulated genes (Fig. e, brown profiles in right panel compared to left panel), despite a higher P-RNAPII ChIP-seq density at TSS in TKO on both Up- and Down-regulated genes (Fig. f, brown profiles in left and right panels).

## Supplementary Fig. 8

a

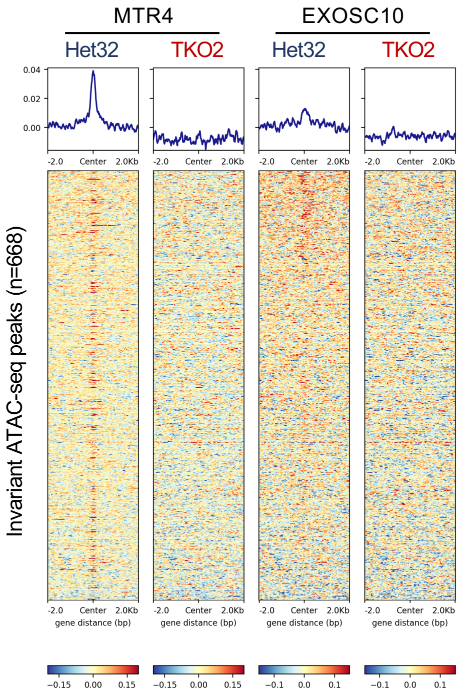


b

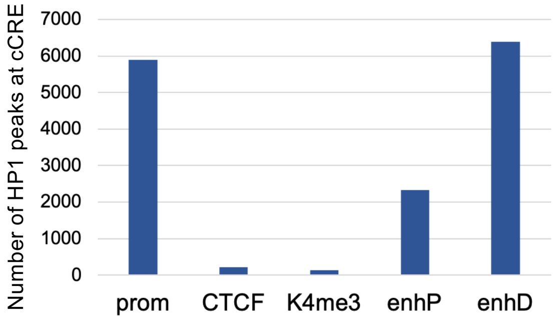


| **Labels** | **ENCODE classifications** |
|---|---|
| Prom | promoter-like signature |
| CTCF | CTCF-only |
| K4me3 | DNase-H3K4me3 |
| enhP | proximal enhancer-like signature |
| enhD | distal enhancer-like signature |

c

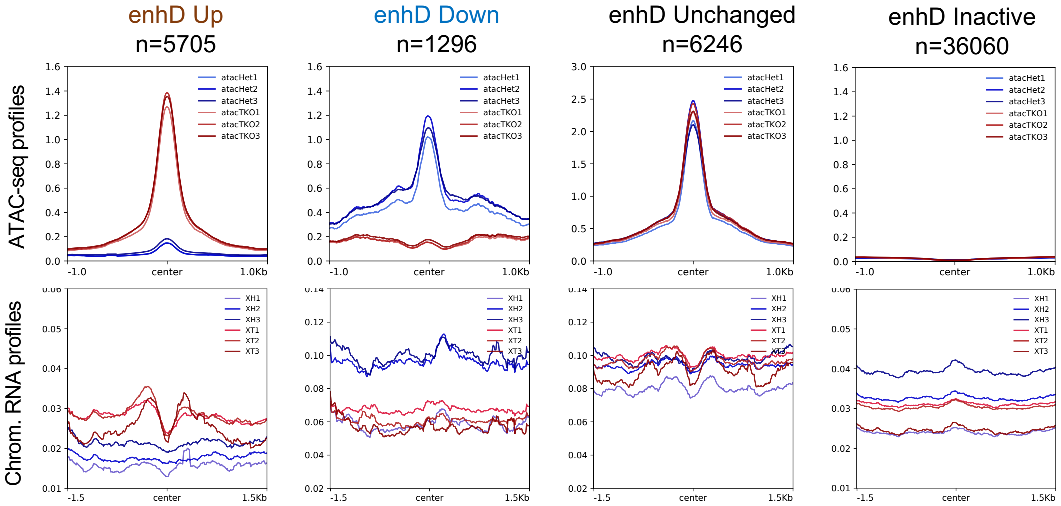

**Supplementary Fig. 8: Up-regulated enhD enhancers are associated with exosome-sensitive chromatin-associated RNA.**

**a.** Heatmaps of ChIPseq densities of MTR4, and EXOSC10 distributed on invariant ATAC-seq peaks displayed in Fig. 5a. **b.** Number of CUT&Tag HP1 merged peaks located at cCRE regulatory elements annotated as indicated on the right. **c.** Average density profiles on enhD elements. ATAC-seq (top panels) and chromatin-associated RNA (bottom panels) density profiles in the three Het (blue profiles) and TKO (red profiles) clones on enhD elements as defined in the cCRE ENCODE database. EnhD were categorized based on their chromatin accessibility by ATAC-seq between Het and TKO. Categories are as follows: Down, with decreased accessibility in TKO cells; Unchanged, active in both conditions; Up, with increased accessibility in TKO cells; inactive, with no ATAC-seq density, respectively. Source data are provided as a Source Data file.

## Supplementary Fig. 9

**a**

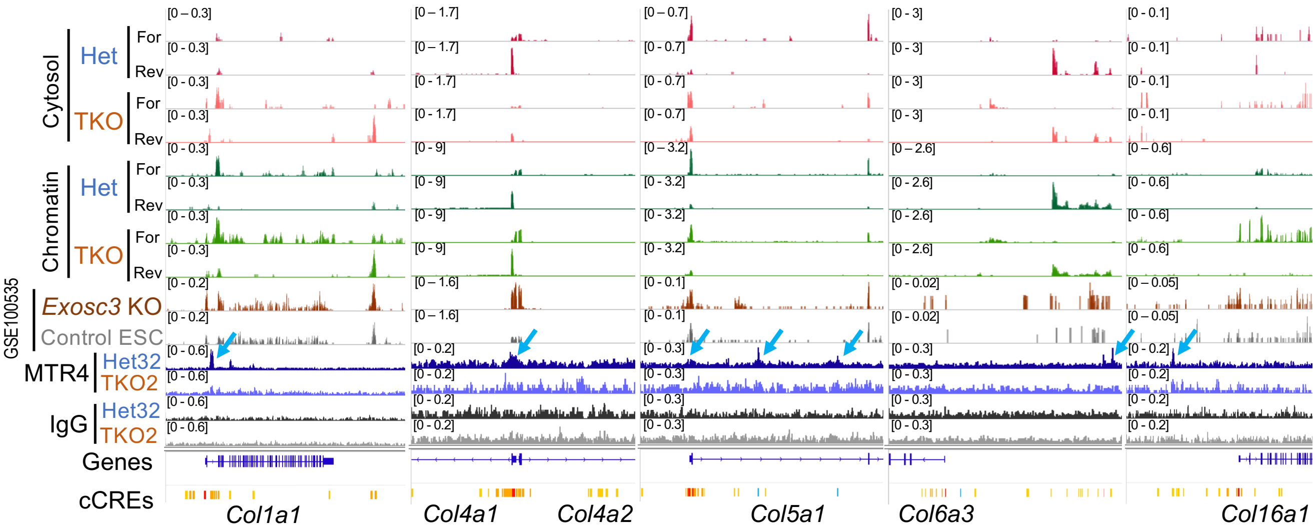


**b**

Collagen Transcriptome TKO vs Het (DESeq2)

| Gene name | BaseMean | log2FC | adj-pVal | |
|---|---|---|---|---|
| Col1a1 | 26 | 2.25 | 4.98E-02 | * |
| Col2a1 | 62 | 5.86 | 2.54E-05 | *** |
| Col4a1 | 4191 | 0.31 | 9.29E-01 | |
| Col4a2 | 2231 | 0.16 | 9.67E-01 | |
| Col4a4 | 34 | 1.28 | 7.31E-01 | |
| Col4a5 | 1575 | -0.80 | 8.08E-01 | |
| Col5a1 | 939 | 0.77 | 9.10E-01 | |
| Col6a1 | 38 | 3.94 | 1.02E-02 | * |
| Col6a2 | 45 | 7.51 | 9.37E-05 | **** |
| Col6a3 | 43 | 4.22 | 1.91E-06 | **** |
| Col7a1 | 153 | 1.62 | 7.91E-02 | |
| Col9a3 | 112 | 0.42 | 8.16E-01 | |
| Col11a2 | 311 | -0.06 | 9.92E-01 | |
| Col12a1 | 91 | -1.61 | 3.88E-01 | |
| Col15a1 | 144 | -1.22 | 3.15E-01 | |
| Col16a1 | 57 | 4.01 | 2.66E-04 | **** |
| Col17a1 | 14 | 1.34 | 6.46E-01 | |
| Col18a1 | 2373 | 0.38 | 7.94E-01 | |
| Col20a1 | 300 | 0.68 | 8.62E-01 | |
| Col24a1 | 36 | 0.62 | 8.92E-01 | |
| Col27a1 | 198 | 2.28 | 3.91E-02 | * |

**c**

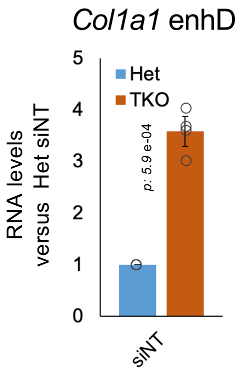


**d**

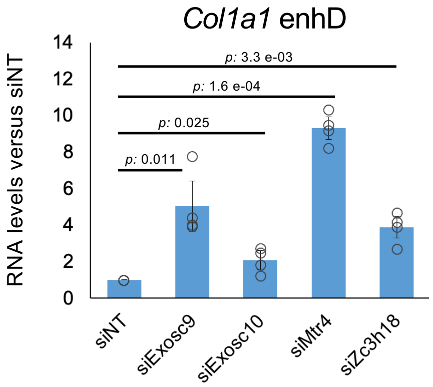

**Supplementary Fig. 9: Collagen genes are sensitive to exosome depletion.**

Increased transcript levels are detected within gene bodies and on several neighboring enhancers upon exosome depletion.

**a.** Transcriptome profiles in Het or TKO chromatin (green) or cytoplasm (red) compared with transcriptome profiles of mESC invalidated for the expression of the EXOSC3 core exosome subunit (*Exosc3* KO, brown) and control mESC (grey) (GSE100535 dataset[3]), together with MTR4 ChIPseq densities in Het32 and TKO2 (dark blue and light blue tracks, respectively) compared to IgG (dark and grey tracks, respectively). Blue arrows highlight MTR4 ChIPseq peaks. **b.** Changes in collagen gene expression in the three Het versus TKO clones by DESeq2 analysis. Base mean, average of the normalized count values divided by gene size factors in both Het and TKO; log2FC, expression fold change in TKO versus Het, with adjusted *p*-values as computed by DESeq2 and adjusted for multiple testing by the Benjamini-Hochberg procedure. **c, d.** RNA levels at an enhancer in the vicinity of the *Col1a1* gene (*Col1a1* enhD) are increased in TKO versus Het cells (**c**). In Het cells, *Col1a1* enhD RNA is stabilized by siRNA-targeted depletion of all the indicated exosome components or cofactors compared to non-targeting (siNT) controls (**d**). Histograms represent mean and average deviation of relative RNA levels quantitated by RT-qPCR versus siNT. *p*-values represent significance of the differences between individual siRNA knockdown and siNT measured by unpaired, one-sided Student's t-test. n= 4 biological replicates. Experiment was repeated with similar results. Source data are provided as a Source Data file.

**Supplementary Fig. 10**

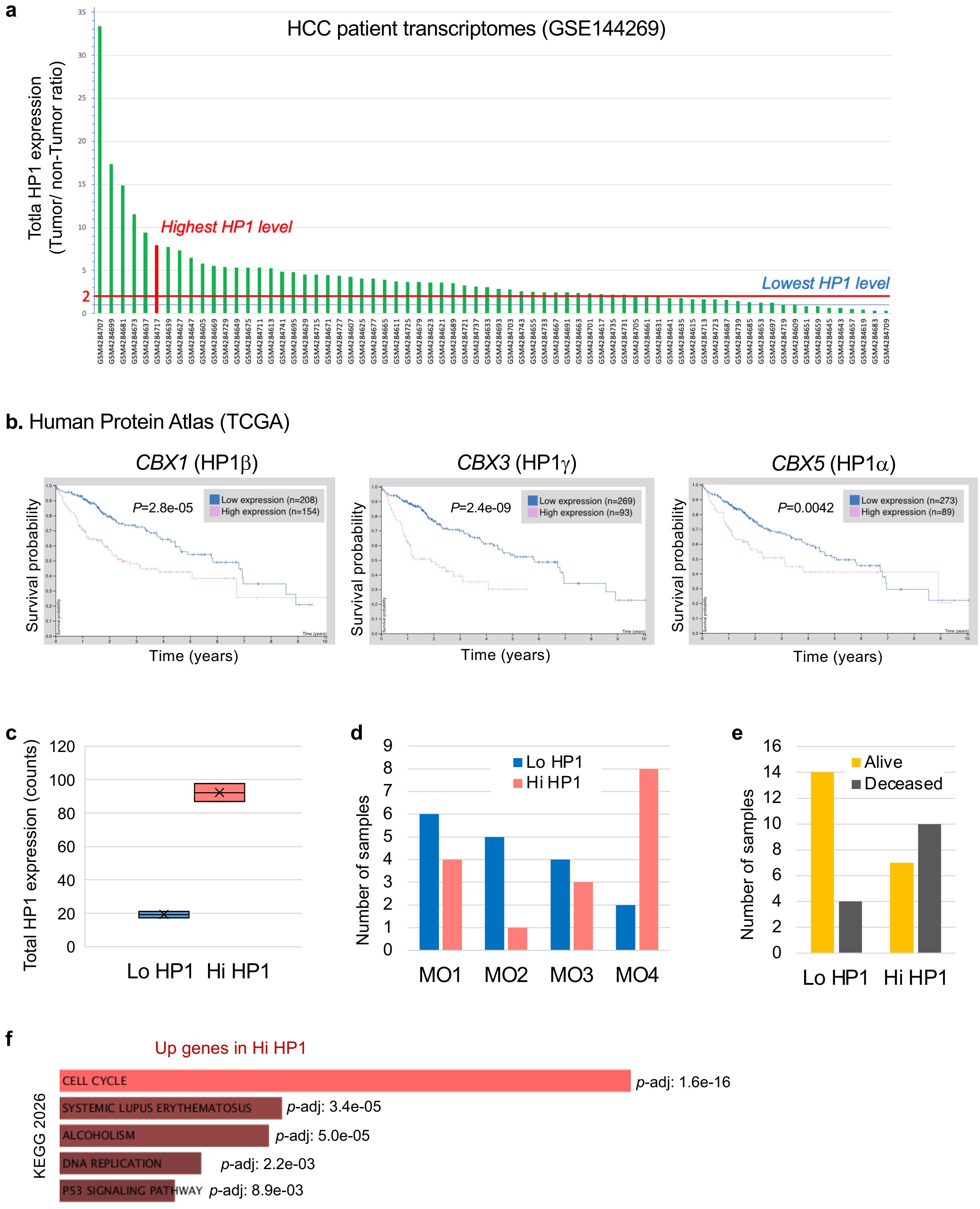

**Supplementary Fig. 10: Metadata associated with the analysis of HCC patient samples.**
**a.** Histogram of total HP1 expression ratio in tumors relative to adjacent non-tumor samples of 76 HCC patient transcriptomes (GSE144269 dataset[4]) **b.** Kaplan-Meyer plots showing the correlation between mRNA expression of *CBX1* (HP1β), *CBX3* (HP1γ), and *CBX5* (HP1α) and liver hepatocellular carcinoma patient survival probabilities, from the Human Protein Atlas[5] upon data generated by the TCGA Research Network. Survival curves for low expression (blue) are compared to high expression (pink) of the indicated genes. P scores indicate log-rank *p* values of correlation between expression levels and patient survival. **c.** Total HP1 expression levels in the two groups of high HP1 (Hi HP1) and Low HP1 (Lo HP1) patients defined by the first and third total HP1 expression quartiles, respectively, related to Fig. 6a. **d, e.** Histograms of the number of Hi HP1 and Lo HP1 patients clustered according to four Molecular subclasses (MO) sorted by increasing severity of the disease from MO1 to MO4 (**d**), or showing survival criteria (**e**), as defined previously[4]. **f.** Histograms of the most significant KEGG (Kyoto Encyclopedia of Genes and Genomes) pathways corresponding to upregulated genes in Hi HP1, analyzed with enrichR. *p*-adj, significance of the pathway analyses by Fisher's exact test, adjusted for multiple hypothesis by the Benjamini-Hochberg procedure. Source data are provided as a Source Data file.

## Supplementary Table 1: Western blot antibodies

| Antibodies | Dilution | References |
|---|---|---|
| Anti-EXOSC3 | 1/1000 | Proteintech 15062-I-AP |
| Anti-EXOSC9 | 1/1000 | Abcam ab156686 |
| Anti-EXOSC10 | 1/1000 | Bethyl A303-987A |
| Anti-MTR4/SKIV2L2 | 1/1000 | Bethyl A300-615A |
| Anti-ZC3H18 | 1/2000 | Bethyl A304-682A |
| Anti-CTCF | 1/1000 | Diagenode A2354-00234P |
| Anti-HP1alpha | 1/1000 | Euromedex IG-2HP-2G9-AS |
| Anti-HP1beta | 1/2000 | Euromedex IG-1MOD-1A9-AS |
| Anti-HP1gamma | 1/3000 | Euromedex IG-2MOD-1G6-AS |
| Anti-H3K9me3 | 1/2000 | Abcam ab8898 |
| Anti-Phospho-Ser5-CTD | 1/1000 | Abcam ab5595 |

## Supplementary Table 2 : RT-qPCR primers

| Primer name | Primer sequence |
|---|---|
| Nabp1-uaRNA-F | GGAGGCTTCAACCAGAAGTATT |
| Nabp1-uaRNA-R | CTGCTGTTCCCATTTCTTCAATC |
| Lyrm2-uaRNA-F | CCTGCGTGCTGTAGGATAAA |
| Lyrm2-uaRNA-R | GAGCACCCTTGCTGTGTATAA |
| ORR1B-1-F | CTTGACTAGAGAGGCAGTTTCC |
| ORR1B-1-R | GCCTAAACCTAGTGGGCTATTC |
| B2-Mm1a-2-F | GTGAAGACTCTGCCTGACTTATG |
| B2-Mm1a-2-R | CCCTCTACTGGAGTGTCTGAA |
| RSINE1-7-F | GAGATTCCAAATGGAGCCTAGAA |
| RSINE1-7-R | CAGCCTCAACCCTATGAATGT |
| enhD-E0883026-F | CTTTCCCAGCCAGCATTAGA |
| enhD-E0883026-R | TGCCAGGATAGCAGAGGATA |
| enhD-E0608019-F | GGGACAAAGGTCTCCAGTG |
| enhD-E0608019-R | GGGCTTCTGCAGTCTTACAT |
| enhD-E0560985-F | TCACCTTCCAAAGCAGTGAG |
| enhD-E0560985-R | CATGCTACCCTTACCCAAAGT |
| Col1a1-enh-F | CTGTAGGAAGCCATCCTGATTAC |
| Col1a1-enh-R | ATGTGAACGCCCAGATGAC |
| Rplp0-mRNA-F | TGAAGTGCTCGACATCACAG |
| Rplp0-mRNA-R | GTACCCATTGATGATGGAGTGT |
| Fosl2-mRNA-F | AGAGATGAGCAGCTGTCTCCTGAAG |
| Fosl2-mRNA-R | AATCTCCTTCTGCAGGCCTGACTT |
| Actb-mRNA-F | CATTGCTGACAGGATGCAGAAGG |
| Actb-mRNA-R | TGCTGGAAGGTGGACAGTGAGG |
| Exosc3-mRNA-F | AGCCAGCGTCTTTGTCTTAC |
| Exosc3-mRNA-R | CCATCTCTGGTTCCATGTCTTT |
| Exosc9-mRNA-F | ACCTCCAACATAGAGGAGAGAG |
| Exosc9-mRNA-R | CCACAGCACAGGTTGAGAAA |
| Exosc10-mRNA-F | AGAAGCAGCCAAGCCTATTC |
| Exosc10-mRNA-R | CCTTCCTCAGTGTTAGGTTCAC |
| Mtr4-mRNA-F | CCCACTCCACAATGATCCTAAC |
| Mtr4-mRNA-R | CTTGCCTTCTTCAGTTCTCTCTT |
| Zc3h18-mRNA-F | AGACACAACACTTGAGCCTTAT |
| Zc3h18-mRNA-R | CTTCTGTATACTGCACCCTGAAA |

## Supplementary methods

### Depletion assays by siRNA knockdown

siRNA-targeted knockdown of Exosc9, Exosc10, Mtr4, and Zc3h18 was performed in Het cells by transient transfection of 20nM ON-TARGETplus siRNA smart pools, or non-targeting control pool (Horizon Discovery Biosciences Ltd) with RNAiMax (Thermo Fisher Sci.) according to the manufacturer's procedure. Cells were harvested 96h post-transfection and nuclear RNA was extracted. Following DNase treatment, relative RNA levels were quantified by RT-qPCR with PCR primer pairs (Eurofins Genomics) in Supplementary Table 2, as described previously[6].

### Actinomycin D time course

Sub-confluent Het cells were treated with 5 µg/ml actinomycin D (Merck) or not (DMSO as a control), and harvested 1, 2, 4, and 7h post treatment. Total RNA was extracted as described in the methods section. Following DNase treatment, relative RNA levels were quantified by RT-qPCR with PCR primer pairs (Eurofins Genomics) in Supplementary Table 2, as described previously[6].

### Data visualization

The patient survival curves in Supplementary Fig. 10b are based upon data generated by the TCGA Research Network: https://www.cancer.gov/tcga, and obtained from the Human Protein Atlas database (https://proteinatlas.org).

## Supplementary references


1. Serdar, L.D. et al. mRNA stability fine-tunes gene expression in the developing cortex to control neurogenesis. *PLoS Biol* **23**, e3003031 (2025).

2. Garland, W. et al. Chromatin modifier HUSH co-operates with RNA decay factor NEXT to restrict transposable element expression. *Mol Cell* **82**, 1691-1707 e8 (2022).

3. Chiu, A.C. et al. Transcriptional Pause Sites Delineate Stable Nucleosome-Associated Premature Polyadenylation Suppressed by U1 snRNP. *Mol Cell* **69**, 648-663 e7 (2018).

4. Candia, J. et al. The genomic landscape of Mongolian hepatocellular carcinoma. *Nat Commun* **11**, 4383 (2020).

5. Uhlen, M. et al. A pathology atlas of the human cancer transcriptome. Science **357**, aan2507 (2017).

6. Rachez, C. et al. HP1gamma binding pre-mRNA intronic repeats modulates RNA splicing decisions. *EMBO Rep* **22**, e52320 (2021).
