# Supplementary material for: Targeting of the nuclear RNA exosome to chromatin by HP1 affects the transcriptional programs of liver cells
Source: Nat Commun. 2026 Apr 29;17:5865. doi: 10.1038/s41467-026-72504-7 (PMC13333803; doi:10.1038/s41467-026-72504-7)
Supplement: Supplementary file 2 — Reporting Summary [file 41467_2026_72504_MOESM2_ESM.pdf]

nature portfolio

Corresponding author(s): Christian Muchardt, Christophe Rachez

Last updated by author(s): Apr 8, 2026

Reporting Summary

Nature Portfolio wishes to improve the reproducibility of the work that we publish. This form provides structure for consistency and transparency in reporting. For further information on Nature Portfolio policies, see our Editorial Policies and the Editorial Policy Checklist.

Statistics

For all statistical analyses, confirm that the following items are present in the figure legend, table legend, main text, or Methods section.

n/a	Confirmed	
☐	☒	The exact sample size (*n*) for each experimental group/condition, given as a discrete number and unit of measurement
☐	☒	A statement on whether measurements were taken from distinct samples or whether the same sample was measured repeatedly
☐	☒	The statistical test(s) used AND whether they are one- or two-sided<br>*Only common tests should be described solely by name; describe more complex techniques in the Methods section.*
☒	☐	A description of all covariates tested
☒	☐	A description of any assumptions or corrections, such as tests of normality and adjustment for multiple comparisons
☐	☒	A full description of the statistical parameters including central tendency (e.g. means) or other basic estimates (e.g. regression coefficient) AND variation (e.g. standard deviation) or associated estimates of uncertainty (e.g. confidence intervals)
☐	☒	For null hypothesis testing, the test statistic (e.g. *F*, *t*, *r*) with confidence intervals, effect sizes, degrees of freedom and *P* value noted<br>*Give P values as exact values whenever suitable.*
☒	☐	For Bayesian analysis, information on the choice of priors and Markov chain Monte Carlo settings
☒	☐	For hierarchical and complex designs, identification of the appropriate level for tests and full reporting of outcomes
☒	☐	Estimates of effect sizes (e.g. Cohen's *d*, Pearson's *r*), indicating how they were calculated

Our web collection on statistics for biologists contains articles on many of the points above.

Software and code

Policy information about availability of computer code

Data collection	Western blots images were acquired on a Chemidoc MP (Biorad), RT-qPCR data were acquired on a Stratagene Mx3005-P quantitative PCR machine. Sequencing on NextSeq 550 and Novaseq 6000

Data analysis	FastQC (version 0.11.9)<br>STAR (version 2.6.0b)<br>Bowtie2 (version 2.3.4)<br>samtools (version 1.8)<br>DESeq2 (version 1.18.1)<br>bedtools (version 2.27.1)<br>DeepTools (version 3.1.3)<br>FeatureCounts (version 1.6.1)<br>MACS2 (version 2.1.1) for ChIPseq and MACS2 (v2.2.9.1) for ATACseq<br>Integrative Genomics Viewer (IGV) (version 2.5.0)<br>Fiji (ImageJ version 2.1.0/1.53c)<br>MxPro qPCR analysis (agilent version 4.10 built 389)<br>Microsoft Excel (version 16.89.1)<br>Microsoft Powerpoint (version 16.106.1)<br>Detailed parameters of each of the programs are mentioned in Methods section.

For manuscripts utilizing custom algorithms or software that are central to the research but not yet described in published literature, software must be made available to editors and reviewers. We strongly encourage code deposition in a community repository (e.g. GitHub). See the Nature Portfolio guidelines for submitting code & software for further information.

Data

Policy information about availability of data

All manuscripts must include a data availability statement. This statement should provide the following information, where applicable:

- Accession codes, unique identifiers, or web links for publicly available datasets
- A description of any restrictions on data availability
- For clinical datasets or third party data, please ensure that the statement adheres to our policy

All RNA-seq, ChIP-seq, ATAC-seq, and CUT&Tag datasets generated during this study have been deposited in the EBI BioStudies database (http://www.ebi.ac.uk/biostudies) under accession codes: E-MTAB-15194, E-MTAB-15196, E-MTAB-15197, and E-MTAB-15189, respectively. The previously published datasets are accessible under accession codes GSE100535 (Exosc3 CKO transcriptome), GSE178550 (MPP8 ChIP-seq and Zcchc8 KO and Zfc3h1 KO transcriptomes), GSE212557 (ZFC3H1 knockdown transcriptome), and GSE144269 (HCC transcriptomes). Web links available in the manuscript.

Research involving human participants, their data, or biological material

Policy information about studies with human participants or human data. See also policy information about sex, gender (identity/presentation), and sexual orientation and race, ethnicity and racism.

Reporting on sex and gender	BMEL murine cells are all male mouse embryonic liver cells.
Reporting on race, ethnicity, or other socially relevant groupings	n/a
Population characteristics	n/a
Recruitment	n/a
Ethics oversight	n/a

Note that full information on the approval of the study protocol must also be provided in the manuscript.

Field-specific reporting

Please select the one below that is the best fit for your research. If you are not sure, read the appropriate sections before making your selection.

☒ Life sciences ☐ Behavioural & social sciences ☐ Ecological, evolutionary & environmental sciences

For a reference copy of the document with all sections, see nature.com/documents/nr-reporting-summary-flat.pdf

Life sciences study design

All studies must disclose on these points even when the disclosure is negative.

Sample size	We did not apply any statistical methods to pre-determine sample size since we analyzed the total number of genomic elements listed in their category from public databases such as RepeatMasker and ENCODE. The number of samples (n) is indicated in all figures. Sample size were determined based on prior experience to obtain statistical significance.
Data exclusions	No data were excluded from the analysis.
Replication	We performed the transcriptome and the ATAC-seq on three biological replicates that were three independent Het and TKO BMEL clones.

ChIP-seq was performed on a single Het32 and TKO2 clone, but immunoprecipitation assays were performed on several independent components of the RNA Exosome complex. Moreover, ChIP assays have been performed several times reproducibly, on independent biological samples analyzed by genome-wide sequencing and/or qPCR along the process of protocol optimization. Only the last experiment is analyzed here for consistency between the described procedure and the resulting outcome.
CUT&Tag was performed on two Het and TKO biological replicates.

Randomization	The three Het clones and three TKO clones were allocated to "Het" and "TKO" groups, respectively. Randomization was not relevant to this study because we did not design any experiment that needed to apply randomization between control group and treatment group.
Blinding	Blinding was not relevant to this study because we did not design any experiment that needed to apply blinding to reduce bias.

Reporting for specific materials, systems and methods

We require information from authors about some types of materials, experimental systems and methods used in many studies. Here, indicate whether each material, system or method listed is relevant to your study. If you are not sure if a list item applies to your research, read the appropriate section before selecting a response.

Materials & experimental systems

n/a	Involved in the study
☐	☒ Antibodies
☐	☒ Eukaryotic cell lines
☒	☐ Palaeontology and archaeology
☒	☐ Animals and other organisms
☐	☒ Clinical data
☒	☐ Dual use research of concern
☒	☐ Plants

Methods

n/a	Involved in the study
☐	☒ ChIP-seq
☒	☐ Flow cytometry
☒	☐ MRI-based neuroimaging

Antibodies

Antibodies used	anti-ZC3H18 (Atlas HPA 040847), anti-P-RNAPII (a mix of antibodies against phospho-Ser5 and phospho-Ser2, Abcam Ab5095 and Ab5408, respectively), anti-murine IgG (Merck), anti-EXOSC3 (Proteintech 15062-I-AP), Anti-EXOSC9 (Abcam ab156686), Anti-EXOSC10 (Bethyl A303-987A), Anti-MTR4/SKIV2L2 (Bethyl A300-615A), Anti-ZC3H18 (Bethyl A304-682A), Anti-CTCF (Diagenode A2354-00234P), Anti-HP1a (Euromedex IG-2HP-2G9-AS), Anti-HP1b (Euromedex 1A9), Anti-HP1g (Euromedex 1G6), Anti-H3K9me3 (Abcam ab8898), anti-H3K27me3 (Cell Signaling Technol. 9733). Secondary antibodies: Starbright Blue 700 anti-rabbit and anti-mouse IgG (Biorad 12004162 and 12004159), or rabbit true blot (Rockland, 18-8816-33).
Validation	The antibodies used in this study are commercially available and have been validated by manufacturer. Antibodies used in Western blot were further validated by comparing the profiles on Cyto-nucleo-chromatin fractions in murine cell lines and also by Western blot under siRNA knockdown of the specific proteins (illustrated in Supplementary Figures 3 and 4).<br>Manufacturer's informations on antibodies:<br>anti-ZC3H18 (Atlas, reference HPA 040847), https://www.atlasantibodies.com/products/primary-antibodies/triple-a-polyclonals/anti-zc3h18-antibody-hpa040847/<br>anti-EXOSC3 (Proteintech, reference 15062-I-AP), https://www.ptglab.com/products/EXOSC3-Antibody-15062-1-AP.htm?srsltid=AfmBOor0WCrVJCktGqcouzD5DoiT1r2ipjqjqoOa9rJav8fwbOvtZPlQ<br>Anti-EXOSC9 (Abcam, reference ab156686), https://www.abcam.com/en-us/products/primary-antibodies/exosc9-antibody-ab156686<br>Anti-EXOSC10 (Bethyl, reference A303-987A), https://www.fortislife.com/products/primary-antibodies/rabbit-anti-exosc10-antibody/BETHYL-A303-987<br>Anti-MTR4/SKIV2L2 (Bethyl, reference A300-615A), https://www.fortislife.com/products/primary-antibodies/rabbit-anti-skiv2l2-antibody/BETHYL-A300-615?selected=A300-615A<br>Anti-ZC3H18 (Bethyl A304-682A), https://www.fortislife.com/products/primary-antibodies/rabbit-anti-zc3h18-antibody/BETHYL-A304-682?selected=A304-682A<br>Anti-CTCF (Diagenode, reference A2354-00234P), https://www.diagenode.com/files/products/antibodies/Datasheet_C15410210_CTCF_LotA2354-00234P.pdf<br>Anti-HP1a (Euromedex, reference IG-2HP-2G9-AS), clone 2HP-2G9, https://shopresearch.euromedex.com/btREC/control/product?productId=REC_IG-2HP-2G9-AS<br>Anti-HP1b (Euromedex, reference IG-1MOD-1A9-AS), clone 1MOD-1A9, https://shopresearch.euromedex.com/btREC/control/product?productId=REC_IG-1MOD-1A9-AS<br>Anti-HP1g (Euromedex, reference IG-2MOD-1G6-AS), clone 2MOD-1G6, https://shopresearch.euromedex.com/btREC/control/product?productId=REC_IG-2MOD-1G6-AS<br>Anti-H3K9me3 (Abcam, reference ab8898), https://www.abcam.com/en-us/products/primary-antibodies/histone-h3-tri-methyl-k9-antibody-chip-grade-ab8898<br>anti-H3K27me3 (Cell Signaling Technology, reference 9733), https://www.cellsignal.com/products/primary-antibodies/tri-methyl-histone-h3-lys27-c36b11-rabbit-monoclonal-antibody/9733

Eukaryotic cell lines

Policy information about cell lines and Sex and Gender in Research

Cell line source(s)	BMEL cells were clones of murine embryonic liver-derived cells (male) cloned by Florence Cammas, as previously described in Saksouk, N. et al. Oncogene 39, 2676-2691 (2020).
Authentication	Het and TKO BMEL clones were authenticated by genotyping. The transcriptome analysis confirmed identity of Het and TKO BMEL clones by visualization of loss of RNA density at KO exons in Cbx1, Cbx3, Cbx5 genes in TKO clones.<br>No sex-based analyses have been performed on BMEL cells since observation of the transcriptomes of BMEL clones revealed no differential expression of the sex-specific genes, Xist and Tsix, between the clones, revealing no sex bias between Het and TKO clones.
Mycoplasma contamination	Cell lines tested negative for mycoplasma contamination in routine tests.
Commonly misidentified lines<br>(See ICLAC register)	none.

Clinical data

Policy information about clinical studies

All manuscripts should comply with the ICMJE guidelines for publication of clinical research and a completed CONSORT checklist must be included with all submissions.

Clinical trial registration	n/a
Study protocol	n/a
Data collection	We reanalyzed RNA-sequencing data from a cohort of 76 Mongolian HCC patients (GSE144269) published in Candia, J. et al. The genomic landscape of Mongolian hepatocellular carcinoma. Nat Commun 11, 4383 (2020). All clinical data originate from the informations associated with this previous study.<br>No sex- and gender-based analyses have been performed on the HCC tumor samples since the original study from Candia et al., 2020, showed no male/female bias in the distribution of the samples into the four MO1-4 severity categories.
Outcomes	n/a

Plants

Seed stocks	n/a
Novel plant genotypes	n/a
Authentication	n/a

ChIP-seq

Data deposition

- [x] Confirm that both raw and final processed data have been deposited in a public database such as GEO.
- [x] Confirm that you have deposited or provided access to graph files (e.g. BED files) for the called peaks.

Data access links<br>*May remain private before publication.*	ChIP-seq: https://www.ebi.ac.uk/biostudies/ArrayExpress/studies/E-MTAB-15196<br>CUT&Tag: https://www.ebi.ac.uk/biostudies/arrayexpress/studies/E-MTAB-15189<br>RNA-seq: https://www.ebi.ac.uk/biostudies/ArrayExpress/studies/E-MTAB-15194<br>ATAC-seq: https://www.ebi.ac.uk/biostudies/ArrayExpress/studies/E-MTAB-15197
Files in database submission	ENA_Samples deposited to ebi database: (RNA-seq, ChIP-seq, ATAC-seq, CUT&Tag)<br><br>ENA_ChIPseq_Samples:<br>ERS24830952 Het32_CTCF_ChIP<br>ERS24830953 Het32_EXOSC10_ChIP<br>ERS24830954 Het32_IgG_ChIP<br>ERS24830955 Het32_MTR4_ChIP<br>ERS24830956 Het32_P-RNAPII_ChIP

ERS24830957 Het32_ZC3H18_ChIP
ERS24830958 TKO2_CTCF_ChIP
ERS24830959 TKO2_EXOSC10_ChIP
ERS24830960 TKO2_IgG_ChIP
ERS24830961 TKO2_MTR4_ChIP
ERS24830962 TKO2_P-RNAPII_ChIP
ERS24830963 TKO2_ZC3H18_ChIP
ENA_ATACseq_Samples:
ERS24830964 Het1_ATACseq
ERS24830965 Het32_ATACseq
ERS24830966 Het35_ATACseq
ERS24830967 TKO1_ATACseq
ERS24830968 TKO2_ATACseq
ERS24830969 TKO3_ATACseq
ENA_CUT&Tag_Samples:
ERS24804320 CUT&Tag_Het_H3-K27me3
ERS24804321 CUT&Tag_Het_H3-K9me3
ERS24804322 CUT&Tag_Het_HP1a-Rep1
ERS24804323 CUT&Tag_Het_HP1a-Rep2
ERS24804324 CUT&Tag_Het_HP1b-Rep1
ERS24804325 CUT&Tag_Het_HP1b-Rep2
ERS24804326 CUT&Tag_Het_HP1g-Rep2
ERS24804327 CUT&Tag_Het_HP1g_Rep1
ERS24804328 CUT&Tag_Het_IgG
ERS24804329 CUT&Tag_TKO_H3-K27me3
ERS24804330 CUT&Tag_TKO_H3-K9me3
ERS24804331 CUT&Tag_TKO_HP1a-Rep1
ERS24804332 CUT&Tag_TKO_HP1a-Rep2
ERS24804333 CUT&Tag_TKO_HP1b
ERS24804334 CUT&Tag_TKO_HP1g
ERS24804335 CUT&Tag_TKO_IgG
ENA_RNA-seq_Samples:
ERS24815826 Het1 chromatin RNAseq
ERS24815827 Het1 cytosolic RNAseq
ERS24815828 Het1 total RNAseq
ERS24815829 Het32 chromatin RNAseq
ERS24815830 Het32 cytosolic RNAseq
ERS24815831 Het32 total RNAseq
ERS24815832 Het35 chromatin RNAseq
ERS24815833 Het35 cytosolic RNAseq
ERS24815834 Het35 total RNAseq
ERS24815835 TKO1 chromatin RNAseq
ERS24815836 TKO1 cytosolic RNAseq
ERS24815837 TKO1 total RNAseq
ERS24815838 TKO2 chromatin RNAseq
ERS24815839 TKO2 cytosolic RNAseq
ERS24815840 TKO2 total RNAseq
ERS24815841 TKO3 chromatin RNAseq
ERS24815842 TKO3 cytosolic RNAseq
ERS24815843 TKO3 total RNAseq

Genome browser session (e.g. UCSC)

no longer applicable

Methodology

Replicates

ChIPseq was performed on Het32 and TKO2 clones.
CUT&Tag was performed on two independent biological replicates of Het and TKO cells.

Sequencing depth

ChIPseq sequencing gave an average of 54Mio 40nt paired-end reads.
CUT&Tag sequencing gave an average of 39Mio reads and 4.5Mio uniquely mapped 40nt paired-end reads.

Antibodies

ChIPseq antibodies:
Anti-MTR4/SKIV2L2 (Bethyl A300-615A), anti-ZC3H18 (Atlas HPA 040847), anti-EXOSC10 (Bethyl A303-987A), anti-CTCF (Diagenode A2354-0023P), anti-P-RNAPII (a mix of antibodies against phospho-Ser5 and phospho-Ser2, Abcam Ab5095 and Ab5408, respectively), anti-rabbit IgG (Merck)
CUT&Tag antibodies:
Anti-HP1alpha (Euromedex IG-2HP-2G9-AS), Anti-HP1beta (Euromedex IG-1MOD-1A9-AS), Anti-HP1gamma (Euromedex IG-2MOD-1G6-AS), Anti-H3K9me3 (Abcam ab8898), anti-H3K27me3 (Cell Signaling Technol. 9733).
See antibodies section for validation details.

Peak calling parameters	ChIPseq Narrow Peak calling was performed using MACS2 (v.2.1.1) (parameters: -p 0.05).
Data quality	Only peaks with a p-value below 0.05 were retained. Peaks were visually inspected as tracks in a genome browser.
Software	Raw ChIP-seq data in Fastq format were subjected to quality control using FastQC (v0.11.9). ChIP-seq reads were mapped to mm10 using bowtie2 (v2.3.4) (parameters: -N 0 -k 1 –very-sensitive-local). We then selected reads with a MAPQ equal or higher than 30 corresponding to uniquely mapped reads for further analysis.<br>CUT&Tag reads were aligned on the mm10 reference genome with bowtie2 (parameters: --end-to-end --very-sensitive --no-mixed --no-discordant -k 1 -X 1000 --phred33 -I 25 -p 24 -x mm10). Bigwig files were generated from two merged HP1 CUT&Tag replicates, and they were normalized using CPM.
