# Supplementary material for: Targeting of the nuclear RNA exosome to chromatin by HP1 affects the transcriptional programs of liver cells
Source: Nat Commun. 2026 Apr 29;17:5865. doi: 10.1038/s41467-026-72504-7 (PMC13333803; doi:10.1038/s41467-026-72504-7)
Supplement: Supplementary file 3 — Transparent Peer Review file [file 41467_2026_72504_MOESM3_ESM.pdf]

nature portfolio

Peer Review File

Targeting of the nuclear RNA exosome to chromatin by HP1 affects the transcriptional programs of liver cells

Corresponding Author: Dr Christophe RACHEZ

This file contains all reviewer reports in order by version, followed by all author rebuttals in order by version.

Version 0:

Reviewer comments:

Reviewer #1

(Remarks to the Author)
This manuscript reports a role for HP1-mediated targeting of the nuclear exosome to a subset of loci in mouse embryonic liver cells and the role of this targeting in gene expression. The authors employ BMEL cells that are either heterozygote for HP1 knockouts or lack all three HP1a, HP1b, and HP1g genes (TKO). They report that the exosome physically interacts with each of the three HP1 proteins, inactivation of all three HP1 proteins leads to accumulation of LTR-derived and enhancer RNAs, increased activity of a subset of liver enhancers, many of which encode collagen genes. In addition, their analysis of hepatocellular carcinoma samples supports a correlation between HP1 expression levels and accumulation of extracellular matrix components.

The results are important and emphasize a still not widely appreciate role for chromosome-associated RNA turnover linked to HP1 in regulation of gene expression. The findings should be of broad general interest to the chromatin, RNA decay, and transcription fields. The main conclusions of the paper are for the most part supported by straightforward experiments that are of high technical quality. There are some gaps in linking HP1 localization and exosome recruitment and the mechanism of HP1 localization to regions that lack H3K9 methylation remains unclear, but addressing these questions may be beyond the scope of the present study. The manuscript is suitable for publication in Nature Communications after the authors address the following points.

1. The co-IP experiments in Fig 1E-G and supplementary Fig 1D lack proper controls. Of course, HP1 signals are absent in TKO cells and this is good evidence for the specificity of the HP1 antibodies used in the study, but the IPs are performed using antibodies that recognize ZC3H18 and ESXOSC9 (or 10) exosome subunits. No controls for the specificity of the latter antibodies are presented. It is therefore not convincing that exosome IPs are enriched for HP1 proteins. The authors should include KD of each exosome subunit to test the specificity of the antibodies they are using. Direct physical interaction between HP1 proteins and the exosome is an important point in the paper. Demonstrating that KD of the tested exosome subunits abolished the observed interactions is critical to support this conclusion. This criticism also applies to the data in Fig. 1C-D, which lack any evidence for the specificity of the various antibodies.

2. Regarding changes in RNA levels in HP1 TKO cells (Fig. 2), it may be difficult to conclude whether the increase in various RNA levels is a result of increased transcription (loss of TGS) or decreased RNA decay. Comparison between the ESC data from reference 38 and the authors' results is difficult since the relative contribution of each pathway to accumulation of LTR RNAs cannot be judged. Also, did the depletion of exosome subunits also result in increased expression of SINE, uRNAs, and enhancer RNAs?

3. Fig. 5. Do exosome subunits localize to collagen genes in an HP1-dependent manner? This evidence is lacking and is required to bring the observations regarding HP1 and MTR4 localization, and role for the exosome together.
4. Adaptation mechanisms in the TKO cells are a concern and are not addressed. Ideally, the authors should complement their studies with rescue of the TKO cells with degron-tagged HP1 followed by acute depletion to uncover direct effects.

5. The authors provide a nice introduction of prior studies linking RNA turn over to heterochromatin-dependent gene silencing. Recent studies have provided direct links between the fission yeast HP1 protein Swi6 and human Polycomb complexes with an RNA decay complex (rixosome) and the XRN2 exonuclease (PMID: 32491985, PMID: 35355014). To put their studies in proper context, the authors should discuss and cite these studies which are highly relevant to their findings.

Minor

1. The overlap between HP1, CTCF, and exosome subunits at some enhancers is interesting. Can the authors speculate how HP1 localizes to these sites in the absence of H3K9 methylation, perhaps via interaction with RNA? If the latter were the case, wouldn't it be expected for HP1 to localize to all transcribed regions?

2. Figure 3 focuses on the association of exosome components with gene bodies. How about the association of HP1 and the exosome with unstable RNA loci such as LTRs highlighted in Figure 2? In any case, a general function for the exosome in regulation of RNA pol II and transcription would be very interesting. This seems like a different function from the regulation of LTR class transcripts.

3. The co-localization of MTR4 with CTCF sounds very interesting and new! Do exosome subunits also co-localize with CTCF?

4. It is difficult to judge the specificity of HP1 upregulation in HCC cells and its correlation with increased mortality. Could the change in HP1 expression reflect a more general de-regulation of gene expression programs and proliferative capacity? (not necessarily causal?)

Reviewer #2

(Remarks to the Author)
In this manuscript, Souaifan et al. describe a novel interaction between HP1, known to be implicated in several co-transcriptional processes, and the RNA exosome, a major player in RNA processing and decay. Using a mouse embryonic liver cell knockout model, they provide a wide range of data (including RNA-seq and Chip-Seq) to propose that HP1 is needed to recruit the RNA exosome to enable degradation of retrotransposon-derived RNAs as well as enhancer RNAs. They then go on to show that a number of liver enhancers are important to regulate extracellular matrix proteins, and that HP1 expression in liver cancer patients correlates with changes in RNA degradation and survival rate.

Generally, the results are interesting to the field and plausible, given previous findings in fission yeast which showed that the HP1 homologue Swi6 "mediates the recognition and destruction of heterochromatic RNA transcripts" (PMID: 22683269), by passing over the RNAs to the RNA exosome co-factor Cid14. Likewise, the RNA exosome has previously been shown to degrade RNA in the context of chromatin, while MS analysis in mammalian cells revealed interactions between all three HP1 isoforms and the exosome co-factors ZC3H18 and MTR4 (which is nicely illustrated in Figure 1A). The results presented in this paper further build on previous findings that depletion of HP1 resulted in the accumulation of normally silenced retroelements (PMID: 32020053).

Overall, most of the experiments are conducted to a high standard and well-described in the methods section, and the RNA-seq data on accumulation of specific RNA species in the absence of HP1 are sound.

However, on page 6 of the manuscript, the authors themselves describe two models by which loss of HP1 may lead to these increased RNA levels:
"HP1 inactivation leads to both reduced recruitment of the RNA degradation machinery and increased recruitment of RNA polymerase II, supporting a model in which HP1 proteins contribute to gene silencing by simultaneously restricting transcription and promoting RNA degradation."

They then provide data for the first model, i.e. the importance of HP1 in the recruitment of the exosome (which is also part of the manuscript title), suggesting that this mode of HP1 action is likely the predominant one.

While this is a plausible scenario, some of the experimental data supporting this hypothesis are relatively weak and/or require further explanation/validation/experiments:

1) Changes of exosome core component (EXOSC3, EXOSC9) and cofactor (ZC3H18, MTR4) localisation upon HP1 loss:
Page 4: "we observed increased accumulation of EXOSC9 and EXOSC3 in the cytosolic fraction, suggesting reduced nuclear retention of these subunits (Fig. 1D, lanes C)."

- The apparent changes between Het32 and TKO2 cells are very subtle. Quantification of this data is needed to support the statement, including repeats of the blot and statistical analysis. Why was EXOSC10 not included in this experiment, given that it is used as the sole EXOSC component in the CHIP-seq experiments in Figure 3?

- It would be good to also include immunofluorescence analysis, especially of EXOSC3, EXOSC9 and EXOSC10 – comparing signals in the nucleoplasm, nucleolus and cytoplasm in the absence and presence of HP1. For EXOSC10, its predominant subnuclear localisation in the nucleolus is not expected to change upon HP1 loss, while reduced retention in the nucleoplasm compared to the cytoplasm might be detected – supporting the authors' idea.

2) Protein-protein interactions between HP1 and the RNA exosome/cofactors:

- The pulldown efficiency is weak (quantification needed) and not very convincing in the co-IP experiments shown in order to

prove a direct interaction. What is the % bound compared to the input? A lot more HP1gamma is bound to ZC3H18 and EXOSC9 in Figure S1 compared to Figure 1 – what are the reasons for this variation?

- Given the weak signals, it would be important to validate the interactions with a reverse co-IP - using an antibody for HP1.

- Given that HP1gamma is shown in the main figure for all interacting proteins, while alpha and beta are only tested for some and shown in the supplemental material – is this suggestive that the gamma isoform is the predominant HP1 isoform to recruit the degradation machinery?
If so, could the authors please provide a rationale for this – for example by highlighting the similarities/differences between the different isoforms in the text and predicting/testing (perhaps with recombinant proteins?) how specific protein domains may interact with the RNA exosome components and its co-factors.

- Given that previous studies have mainly focussed on the interaction between HP1 homologues and RNA via its unstructured hinge region, more detail on the specific protein-protein-interactions (that are even detected in the absence of RNA as shown for ZC3H18 and EXOSC9) and the domains involved would be useful.
Why was EXOSC10 not included in the RNase A analysis (see comment about CHIP-seq above)?

- EXOSC9 appears to precipitate ZC3H18 in the absence (but not the presence) of RNA (Figure S1 F), but not vice versa. Please comment.

3) Throughout the text, the terms “RNA exosome” or “exosome activity” are often used when referring to analysis of exosome co-factors. This is not correct as depletion/manipulation of core RNA exosome components such as EXOSC3 and 9, exosome associated nucleases (EXOSC10) or co-factors (such as ZC3H18 and MTR4) may yield very different data (as not all exosome targets are dependent on the same co-factor).
Please only use the term “RNA exosome” when talking about EXOSC components, but not co-factors.

- Currently, RNA-seq data shown in Figure 2 convincingly shows changes in RNA levels in TKO cells. However, it is not clear how this relates to profiles seen upon EXOSC depletion. Therefore, please also include RNA-seq data for an EXOSC KO/KD in Figure 2H, for example the EXOSC3 (GSE100535) dataset used in Figure 5F.

- In addition, it would be essential to deplete at least one EXOSC component (as well as ZC3H18 and MTR4) in the Het32 and TKO2 cells and compare levels of selected RNAs (for example by qRT-PCR) to control cells treated with a non-targeting siRNA. This analysis would help to distinguish RNAs accumulating due to the proposed HP1-mediated exosome recruitment from those that are just RNA exosome/cofactor dependent (but not requiring HP1).
In addition, it might also help to clarify the role of HP1 in transcription of these RNAs.

4) The CHIP-seq data presented in Figure 3 show clear changes between Het32 and TKO2 cells upon analysis of all factors except EXOSC10. While the global analysis in panel B indicates a change for EXOSC10, the examples shown in panel A are not very convincing as the peaks for EXOSC10 are barely above background (IgG) level and do not seem to change much between the cells.

- Please comment and provide better examples of EXOSC10 profiles if possible.

- Why was EXOSC10 chosen for this experiment (see comments above), but not EXOSC3 and 9 – please explain.

Minor points:
Page 4, line 177 –“In parallel, RNA-seq was also performed on total RNA from each clone.”
- Please include this data in the relevant figures.
Figure 1: Please change MTREX to MTR4 in panel A to provide consistency.
Figure S1:
B) This should be spelled Tubulin (not Tubuline).
C) Values on peak height are far too small (similar in most other profile images).
Figure S3: The title “Simple repeats” is cropped at the bottom in panel B.

Reviewer #3

(Remarks to the Author)
In this manuscript from Souaifan and co-authors they discover a link between HP1 and RNA exosome. They posit that HP1 recruits the RNA exosome to chromatin to modulate the stability of RNAs, particularly TE-containing non-coding RNAs. They ultimately suggest a link between HP1 function and collagen genes that may have implications for HCC. Overall the study is interesting. I have some comments on the analysis and I have concerns about the way the authors define stability.

Main comments:

1. The description of ZC3H18 is confusing. On line 151 It’s a part of the NEXT complex, but in Fig 1A it’s part of the cap-binding proteins. I think the description on line 104 seems more accurate (adaptor to the NEXT complex) as, as far as I am aware, Zc3H18 is not a common part of the NEXT complex. The authors need to clarify these descriptions in the text and

figures.

2. The authors claim "…the chromatin fraction was expected to be enriched in nascent and incompletely processed RNAs, whereas the cytosolic fraction was expected to be depleted of unstable nuclear transcripts and enriched in mature mRNAs.". The assumption seems to be that because an RNA is bound to chromatin its unstable. But this is not true in other cell types, and the authors do not provide any data that this is correct. DNA-interacting RNAs can be just as stable as their cytoplasmic RNAs. Sometimes more so as RNA degradation tends to be reduced in the nucleus. Either the authors need to provide experimental evidence these RNAs are unstable (e.g. Actinomycin time course), or rephrase this and subsequent text referring to the chromatin-bound RNAs as 'unstable'.

3. Similarly, I am confused by the mixing of stability versus up and down-regulation. For lines 203-210, the authors claim HP1 loss "stabilizes normally unstable non-coding RNAs". However and alternative explanation is that HP1 loss leads to reduced epigenetic repression, reduced heterochromatin and increased transcription. Differential Expression is not a measure of just stability (Figure 2E). Indeed, later in Fig 3C RNAPII levels are increased in the TKO2, suggesting increased transcription, not changes in stability.

4. Line 209: Why is HP1 loss specific for non-coding RNAs? Also, where is the data supporting this? It seems to be based on the assumption TEs are non-coding, but TEs are in about 25% or 5' and 5' UTRs of mRNAs. The analysis needs to be expanded here.

5. Line 214: The claim is RNA exosome complex is less well retained in the nucleus in HP1 TKO. This seems to be based on Fig 1D. However, only EXOSC9 seems to leak into the cytoplasm, and the nuclear proportion of the RNA exosome seems the same or higher as the Het cell lines.

6. Figure 3C, D: This is a cool result. But it does not support the authors model about RNA stability, and the alternative explanation is more compelling that loss of HP1 leads to increased transcription, which explains the majority of the effects the authors see. I'd also like to see this analysis, but restricted to HP1-bound loci, which is partially done in Figure 4, but does not include the EXOSC and ZC3H18 ChIP.

7. Fig 4E: Again, increased POLII supports increased transcription, not changes in RNA stability.

8. I don't understand the relevance of CTCF here. Only MTR4 is recruited to CTCF, not the rest of the RNA exosome complex, and CTCF binding sites change only marginally in the Het vs. TKO cells. Anyway, the study is about HP1 function, so the relevance here is unclear. This extends into Figure 5, why not just ignore CTCF and analyse everything from a HP1 perspective?

Version 1:

Reviewer comments:

Reviewer #1

(Remarks to the Author)
The authors have addressed my concerns satisfactorily and I support publication.

Reviewer #2

(Remarks to the Author)
In this revised version of the manuscript, the authors have addressed all my previous concerns, either by adding new data as requested or, where this was not technically feasible, by revising the text to provide clarification and/or avoid overinterpretation.

I would therefore suggest that the manuscript is now suitable for publication in Nature Communications.

Reviewer #3

(Remarks to the Author)
The authors have addressed my concerns.

Open Access This Peer Review File is licensed under a Creative Commons Attribution 4.0 International License, which permits use, sharing, adaptation, distribution and reproduction in any medium or format, as long as you give appropriate credit to the original author(s) and the source, provide a link to the Creative Commons license, and indicate if changes were made.
In cases where reviewers are anonymous, credit should be given to 'Anonymous Referee' and the source.
The images or other third party material in this Peer Review File are included in the article’s Creative Commons license, unless indicated otherwise in a credit line to the material. If material is not included in the article’s Creative Commons license and your intended use is not permitted by statutory regulation or exceeds the permitted use, you will need to obtain permission directly from the copyright holder.
To view a copy of this license, visit https://creativecommons.org/licenses/by/4.0/

Point-by-point response to the reviewers' comments

REVIEWER COMMENTS

Reviewer #1 (Remarks to the Author):

This manuscript reports a role for HP1-mediated targeting of the nuclear exosome to a subset of loci in mouse embryonic liver cells and the role of this targeting in gene expression. The authors employ BMEL cells that are either heterozygote for HP1 knockouts or lack all three HP1a, HP1b, and HP1g genes (TKO). They report that the exosome physically interacts with each of the three HP1 proteins, inactivation of all three HP1 proteins leads to accumulation of LTR-derived and enhancer RNAs, increased activity of a subset of liver enhancers, many of which encode collagen genes. In addition, their analysis of hepatocellular carcinoma samples supports a correlation between HP1 expression levels and accumulation of extracellular matrix components.

The results are important and emphasize a still not widely appreciate role for chromosome-associated RNA turnover linked to HP1 in regulation of gene expression. The findings should be of broad general interest to the chromatin, RNA decay, and transcription fields. The main conclusions of the paper are for the most part supported by straightforward experiments that are of high technical quality. There are some gaps in linking HP1 localization and exosome recruitment and the mechanism of HP1 localization to regions that lack H3K9 methylation remains unclear, but addressing these questions may be beyond the scope of the present study. The manuscript is suitable for publication in Nature Communications after the authors address the following points.

1. The co-IP experiments in Fig 1E-G and supplementary Fig 1D lack proper controls. Of course, HP1 signals are absent in TKO cells and this is good evidence for the specificity of the HP1 antibodies used in the study, but the IPs are performed using antibodies that recognize ZC3H18 and ESXOSC9 (or 10) exosome subunits. No controls for the specificity of the latter antibodies are presented. It is therefore not convincing that exosome IPs are enriched for HP1 proteins. The authors should include KD of each exosome subunit to test the specificity of the antibodies they are using. Direct physical interaction between HP1 proteins and the exosome is an important point in the paper. Demonstrating that KD of the tested exosome subunits abolished the observed interactions is critical to support this conclusion. This criticism also applies to the data in Fig. 1C-D, which lack any evidence for the specificity of the various antibodies.

We thank the reviewer for this important point. We have now performed two complementary sets of controls to directly address antibody specificity and the dependence of HP1 co-IP on exosome proteins.

First, all antibodies used for immunoprecipitation (EXOSC9, EXOSC10, MTR4, ZC3H18 and HP1γ) were tested by Western blot in Het and HP1 triple knockout (TKO2) extracts (new Supplementary Fig. 2). While anti-HP1γ detects a single ~25 kDa band present in Het and absent in TKO extracts, none of the exosome-directed antibodies show any signal in the 25 kDa range in either condition. This excludes cross-reactivity of exosome antibodies with HP1 proteins.

Second, we performed siRNA-mediated depletion of each exosome component (EXOSC9, EXOSC10, MTR4, ZC3H18) followed by co-IP (new Supplementary Fig. 3-4). Knockdown efficiency was validated by RT-qPCR and Western blot. In each case, depletion of the targeted exosome protein resulted in a

proportional reduction of both the immunoprecipitated exosome component/cofactor and the co-immunoprecipitated HP1γ signal, compared to siNT controls.

Thus, HP1 co-IP strictly depends on the presence of the corresponding exosome/cofactor protein, supporting the specificity of the antibodies and the conclusion that HP1 associates with the RNA exosome complexes.

2. Regarding changes in RNA levels in HP1 TKO cells (Fig. 2), it may be difficult to conclude whether the increase in various RNA levels is a result of increased transcription (loss of **TGS**) or decreased RNA **decay**. Comparison between the ESC data from reference 38 and the authors' results is difficult since the relative contribution of each pathway to accumulation of LTR RNAs cannot be judged. Also, did the depletion of exosome subunits also result in increased expression of SINE, uRNAs, and enhancer RNAs?

We agree that HP1 proteins are well-established transcriptional repressors, and loss of HP1 is therefore expected to impact both transcriptional repression and RNA stability. Our objective is not to attribute the observed RNA accumulation exclusively to defective decay, but to determine whether impaired RNA degradation contributes substantially to the phenotype.

In the revised manuscript, we provide three complementary lines of evidence supporting a contribution of RNA decay.

(1) Overlap between HP1-dependent and exosome-dependent targets.
We now directly compare HP1 TKO RNA-seq data with RNA-seq profiles from EXOSC3 knockout cells (GSE100535; Chiu et al., 2018), replacing the previous comparison to ZCCHC8 and ZFC3H1 knockouts. The new Fig. 2H shows representative loci where RNA accumulation in TKO cells mirrors that observed upon EXOSC3 loss. This concordance extends genome-wide (Fig. 5 and Supplementary Fig. 8), where enhancers upregulated in TKO cells (enhD up) also display increased RNA levels in EXOSC3 KO cells.

In addition, siRNA-mediated depletion of EXOSC9, EXOSC10, MTR4, or ZC3H18 in Het cells results in increased RNA levels at SINEs, uaRNAs, and enhancer RNAs that are upregulated in TKO cells (new Supplementary Fig. 6). Together, these results indicate that many RNAs accumulating in HP1-deficient cells are normally controlled by RNA exosome–dependent degradation.

(2) RNA accumulation at loci with unchanged chromatin accessibility.
At genomic regions where ATAC-seq signal remains unchanged between Het and TKO cells, RNA levels are nevertheless increased in TKO cells (Fig. 5A). These same loci also display elevated RNA density in EXOSC3 KO cells. Under the reasonable assumption that stable ATAC-seq signal reflects unchanged transcriptional input, this observation strongly supports reduced RNA decay as a contributor to RNA accumulation at these sites.

(3) Direct measurement of RNA stability.
To directly assess RNA stability, we performed an Actinomycin D time course in HP1-expressing Het cells (new Supplementary Fig. 5B). Representative LTR, SINE, and enhancer transcripts show substantial decay over time compared to the stable Rplp0 mRNA control, supporting their classification as relatively unstable transcripts under our experimental conditions.

Taken together, these results demonstrate that impaired RNA degradation makes a significant contribution to RNA accumulation upon HP1 loss, although transcriptional derepression likely also contributes to this RNA accumulation.

3. Fig. 5. Do exosome subunits localize to collagen genes in an HP1-dependent manner? This evidence is lacking and is required to bring the observations regarding HP1 and MTR4 localization, and role for the exosome together.

In the revised manuscript, we now show that MTR4 displays clear ChIP-seq enrichment at several collagen genes (Col1a1, Col6a3, Col4a1/Col4a2, Col5a1, Col8a1…). These MTR4 peaks are reduced in HP1 TKO cells, demonstrating HP1-dependent recruitment of MTR4 to these loci (new Supplementary Fig. 9A).
As reads were difficult to pick up at several of these genes in the RNA-seq data, we validated by RT-qPCR analyses that depletion of MTR4 leads to accumulation of eRNAs at the Col1a1 locus (Supplementary Figure 9B).

4. Adaptation mechanisms in the TKO cells are a concern and are not addressed. Ideally, the authors should complement their studies with rescue of the TKO cells with degron-tagged HP1 followed by acute depletion to uncover direct effects.

We agree that long-term adaptation may have occurred in the TKO cells and represents a limitation of the model. Indeed, TKO clones display secondary alterations (e.g., dysregulation of p53 and p21), indicating that compensatory mechanisms can develop upon sustained HP1 loss. Experiments based on degron-mediated acute depletion would be valuable to dissect immediate effects of HP1 removal. However, generation and validation of such a system were not feasible within the timeframe of this revision.
In addition, we believe that the TKO model remains a valuable source of information, as it may reflect a chronic physio-pathological context, such as progressive liver disease leading to tumorigenesis, in which prolonged HP1 deficiency and RNA misregulation contribute to disease-associated transcriptional and inflammatory states. Markers consistent with such chronic alterations are detectable in TKO cells.
We have now clarified this limitation and its implications in the revised Discussion.

5. The authors provide a nice introduction of prior studies linking RNA turn over to heterochromatin-dependent gene silencing. Recent studies have provided direct links between the fission yeast HP1 protein Swi6 and human Polycomb complexes with an RNA decay complex (rixosome) and the XRN2 exonuclease (PMID: 32491985, PMID: 35355014). To put their studies in proper context, the authors should discuss and cite these studies which are highly relevant to their findings.

We have now added two publications on the Rixosome complex in S. pombe (1) and in human cells (2) :

1- G Shipkovenska, A Durango, M Kalocsay, S P Gygi, D Moazed. Elife. 2020 Jun 3:9:e54341. PMID: 32491985
2- Haining Zhou, C B Stein, T A Shafiq, G Shipkovenska, M Kalocsay, J A Paulo, J Zhang, Z Luo, S P Gygi, K Adelman, Danesh Moazed. Nature. 2022 Apr;604(7904):167-174.
PMID: 35355014

Minor

1. The overlap between HP1, CTCF, and exosome subunits at some enhancers is interesting. Can the authors speculate how HP1 localizes to these sites in the absence of H3K9 methylation, perhaps via interaction with RNA? If the latter were the case, wouldn't it be expected for HP1 to localize to all transcribed regions?

We agree that HP1 recruitment to active enhancers in the absence of H3K9 methylation likely involves mechanisms distinct from canonical heterochromatin targeting. Previous work has shown that HP1γ associates with nascent RNA on chromatin, and this association correlates with active chromatin marks rather than H3K9me2/3-enriched regions (Rachez et al., 2021). RNA-mediated recruitment is therefore a plausible mechanism.

Importantly, our CUT&Tag analyses detect HP1 proteins broadly across expressed genes, indicating that HP1 association with active chromatin is not restricted to isolated loci. However, despite this widespread occupancy, only a subset of genes and enhancers display strong transcriptional or RNA-stability phenotypes upon HP1 depletion. This suggests that HP1 presence is relatively general, whereas its functional contribution is context-dependent and may vary according to the dominant regulatory mechanism operating at each locus.

We have clarified this point in the revised Discussion to distinguish widespread recruitment from locus-specific functional sensitivity.

2. Figure 3 focuses on the association of exosome components with gene bodies. How about the association of HP1 and the exosome with unstable RNA loci such as LTRs highlighted in Figure 2? In any case, a general function for the exosome in regulation of RNA pol II and transcription would be very interesting. This seems like a different function from the regulation of LTR class transcripts.

We agree that transcription at LTRs may represent a mechanistically distinct phenomenon.
As shown in Figure 2, LTRs are an abundant source of chromatin-associated transcripts in HP1 TKO cells. However, the LTRs that accumulate RNA represent only a very small subset of the thousands of LTR-derived sequences present in the mouse genome. This raises an important question: which LTRs are affected? There seems to be two non-mutually exclusive possibilities. (1) Some may be LTRs normally kept transcriptionally repressed because they pose a potential threat. (2) Other LTRs may have been co-opted as regulatory elements (e.g., enhancers), and in the TKOs, their associated RNAs are no longer properly degraded.
It is very possible that these two categories are regulated by distinct mechanisms. In the present study, our focus lies primarily on the second case — regulatory elements that produce unstable RNAs whose accumulation reflects impaired RNA decay rather than derepression of repetitive elements. To clarify this, we have refocused Figure 5 on regulatory elements. The former Supplementary Fig. 5D on LTRs has been removed, and we have added new panels showing that intergenic ATAC-seq peaks (putative regulatory regions) are sites (1) where chromatin-bound RNAs accumulate in the TKO cells, (2) where MTR4 and EXOSC10 occupancy peaks in Het cells, but not in TKO cells.

3. The co-localization of MTR4 with CTCF sounds very interesting and new! Do exosome subunits also co-localize with CTCF?

We have now added a new heatmap panel in Fig. 4E showing the density of EXOSC10 together with HP1, CTCF, and MTR4. This panel shows the colocalization of all these proteins on gene loci.

4. It is difficult to judge the specificity of HP1 upregulation in HCC cells and its correlation with increased mortality. Could the change in HP1 expression reflect a more general de-regulation of gene expression programs and proliferative capacity? (not necessarily causal?)

We agree that our HCC analysis does not demonstrate causality. The patient data establish a correlation between HP1 expression levels, altered RNA stability signatures (Hi/Lo RNA ratio), and clinical outcome, but they do not prove that HP1 upregulation is itself the primary driver of mortality.

It is indeed possible that changes in HP1 expression reflect broader deregulation of transcriptional programs and proliferative capacity in aggressive tumors. HP1 proteins have been implicated in regulation of cell proliferation and lineage specification in multiple contexts, supporting the idea that altered HP1 levels may integrate into wider oncogenic programs.

Our intention is therefore not to claim a direct causal role, but to show that HP1 expression levels correlate with a measurable RNA stability signature and provide prognostic stratification in HCC patients. We have clarified this point in the revised manuscript to avoid overinterpretation.

Reviewer #2 (Remarks to the Author):

In this manuscript, Souaifan et al. describe a novel interaction between HP1, known to be implicated in several co-transcriptional processes, and the RNA exosome, a major player in RNA processing and decay. Using a mouse embryonic liver cell knockout model, they provide a wide range of data (including RNA-seq and Chip-Seq) to propose that HP1 is needed to recruit the RNA exosome to enable degradation of retrotransposon-derived RNAs as well as enhancer RNAs. They then go on to show that a number of liver enhancers are important to regulate extracellular matrix proteins, and that HP1 expression in liver cancer patients correlates with changes in RNA degradation and survival rate.

Generally, the results are interesting to the field and plausible, given previous findings in fission yeast which showed that the HP1 homologue Swi6 “mediates the recognition and destruction of heterochromatic RNA transcripts” (PMID: 22683269), by passing over the RNAs to the RNA exosome co-factor Cid14. Likewise, the RNA exosome has previously been shown to degrade RNA in the context of chromatin, while MS analysis in mammalian cells revealed interactions between all three HP1 isoforms and the exosome co-factors ZC3H18 and MTR4 (which is nicely illustrated in Figure 1A). The results presented in this paper further build on previous findings that depletion of HP1 resulted in the accumulation of normally silenced retroelements (PMID: 32020053).(Saksouk 2020)

Overall, most of the experiments are conducted to a high standard and well-described in the methods section, and the RNA-seq data on accumulation of specific RNA species in the absence of HP1 are sound.

However, on page 6 of the manuscript, the authors themselves describe two models by which loss of HP1 may lead to these increased RNA levels:
“HP1 inactivation leads to both reduced recruitment of the RNA degradation machinery and increased recruitment of RNA polymerase II, supporting a model in which HP1 proteins contribute to gene silencing by simultaneously restricting transcription and promoting RNA degradation.”

They then provide data for the first model, i.e. the importance of HP1 in the recruitment of the exosome (which is also part of the manuscript title), suggesting that this mode of HP1 action is likely the predominant one.

While this is a plausible scenario, some of the experimental data supporting this hypothesis are relatively weak and/or require further explanation/validation/experiments:

1) Changes of exosome core component (EXOSC3, EXOSC9) and cofactor (ZC3H18, MTR4) localisation upon HP1 loss:

Page 4: "we observed increased accumulation of EXOSC9 and EXOSC3 in the cytosolic fraction, suggesting reduced nuclear retention of these subunits (Fig. 1D, lanes C)."

- The apparent changes between Het32 and TKO2 cells are very subtle. Quantification of this data is needed to support the statement, including repeats of the blot and statistical analysis. Why was EXOSC10 not included in this experiment, given that it is used as the sole EXOSC component in the CHIP-seq experiments in Figure 3?

- It would be good to also include immunofluorescence analysis, especially of EXOSC3, EXOSC9 and EXOSC10 – comparing signals in the nucleoplasm, nucleolus and cytoplasm in the absence and presence of HP1. For EXOSC10, its predominant subnuclear localisation in the nucleolus is not expected to change upon HP1 loss, while reduced retention in the nucleoplasm compared to the cytoplasm might be detected – supporting the authors' idea.

We thank the reviewer for these constructive suggestions. To avoid overinterpretation, we have clarified these points in the revised manuscript and tempered the wording regarding cytosolic accumulation.
The primary objective of Fig. 1C–D was to document that overall expression levels and gross subcellular distribution of exosome components are largely maintained between Het32 and TKO2 cells.
We have now repeated the fractionation experiments and included densitometric quantification of EXOSC9 from independent biological replicates with statistical analysis. These data confirm that EXOSC3, EXOSC9, and associated cofactors show minor shifts in nuclear/cytoplasmic distribution upon HP1 loss. We believe that this observation remains interesting for reader, but please note that our main conclusion does not rely on major relocalization of exosome components, but rather on their precise HP1-dependent targeting to specific sites on chromatin.
We have also added Western blot analysis of EXOSC10 in both total extracts and subcellular fractions, showing no significant change in its abundance or distribution between Het and TKO cells. EXOSC10 was not included in the initial panel due to space constraints, but we agree that its inclusion is appropriate given its use in the ChIP-seq experiments.
Regarding immunofluorescence, we performed IF analyses of EXOSC10 and did not detect obvious differences in its nucleolar or nucleoplasmic localization between Het and TKO cells. We would prefer not to show these data that only confirm the Western blots and are more difficult to quantify.

2) Protein-protein interactions between HP1 and the RNA exosome/cofactors:

- The pulldown efficiency is weak (quantification needed) and not very convincing in the co-IP experiments shown in order to prove a direct interaction. What is the % bound compared to the input? A lot more HP1gamma is bound to ZC3H18 and EXOSC9 in Figure S1 compared to Figure 1 – what are the reasons for this variation?

We agree that quantitative assessment is necessary. In the revised figures, we now indicate the immunoprecipitation efficiency, expressed as the percentage of input recovered in each IP. HP1γ co-immunoprecipitation represents a small fraction of total input, which is consistent with a substoichiometric and likely dynamic association between chromatin-associated complexes.

The apparent differences in HP1γ signal intensity between Fig. 1 and Fig. S1 reflect independent biological experiments performed with distinct lysate preparations and antibody incubations.

When normalized to input and IP efficiency, the relative enrichment of HP1γ is comparable across experiments. We have now included quantification to allow direct comparison.

To further support specificity, we performed co-IP experiments following siRNA-mediated depletion of individual exosome components. In each case, reduction of the targeted exosome protein leads to a proportional decrease in HP1γ co-immunoprecipitation (new Supplementary Fig. 4). Quantification from 2–3 independent experiments is provided. These results demonstrate that HP1γ recovery depends on the presence of the corresponding exosome component, supporting the specificity of the association.

We note that co-immunoprecipitation assays demonstrate association within a complex but do not by themselves establish direct binary binding, and we have adjusted the manuscript wording accordingly.

- Given the weak signals, it would be important to validate the interactions with a reverse co-IP - using an antibody for HP1.

We agree that reverse co-immunoprecipitation would strengthen the interaction data. We attempted co-IP using antibodies directed against endogenous HP1 proteins; however, under our experimental conditions, we were unable to reproducibly detect exosome components in HP1 immunoprecipitates.

We believe this limitation is technical rather than biological. HP1 proteins are small (~25 kDa), highly abundant chromatin-associated proteins, and the available monoclonal antibodies recognize epitopes spanning substantial portions of the protein. It is therefore possible that antibody binding interferes with interaction interfaces or reduces recovery of associated complexes. In addition, HP1–exosome association appears substoichiometric and chromatin-dependent, which may further limit detection in reverse IP configurations.

Consistent with this, in previous studies we successfully immunoprecipitated HP1γ and identified interacting partners using FLAG-tagged HP1 constructs rather than endogenous antibodies (Zaidan et al., 2017; Rachez et al., 2021). These precedents support the existence of the interaction, although they rely on epitope-tagged systems.

Importantly, the specificity of the association in the present study is supported by reciprocal dependency experiments: depletion of individual exosome components results in proportional loss of HP1 co-immunoprecipitation (new Supplementary Fig. 4). We have clarified these points in the revised manuscript.

- Given that HP1gamma is shown in the main figure for all interacting proteins, while alpha and beta are only tested for some and shown in the supplemental material – is this suggestive that the gamma isoform is the predominant HP1 isoform to recruit the degradation machinery?
If so, could the authors please provide a rationale for this – for example by highlighting the similarities/differences between the different isoforms in the text and predicting/testing (perhaps with recombinant proteins?) how specific protein domains may interact with the RNA exosome components and its co-factors.

We do not interpret our data as indicating that HP1γ is uniquely responsible for recruiting the RNA degradation machinery. CUT&Tag analyses reveal broadly similar chromatin localization profiles for all three HP1 isoforms, and co-immunoprecipitation experiments detect association of

EXOSC components with HP1α, HP1β, and HP1γ (Suppl. Fig. 1D), supporting a degree of functional redundancy.

We focused on HP1γ in the main figures primarily because it shows robust and reproducible detection in our biochemical assays and is the most prominent isoform within gene bodies in our system. We therefore used HP1γ as a representative isoform for clarity and consistency across experiments.

Based on domain organization, all three isoforms share highly conserved chromodomain and chromoshadow domains, which mediate chromatin binding and protein–protein interactions, respectively. It is therefore plausible that recruitment of exosome components relies on conserved structural features rather than γ-specific sequences. However, we have not performed recombinant domain-mapping experiments to test isoform-specific interactions, and we have tempered the text to avoid implying isoform specificity.

Overall, our data support partial redundancy among HP1 isoforms rather than a γ-exclusive mechanism.

- Given that previous studies have mainly focussed on the interaction between HP1 homologues and RNA via its unstructured hinge region, more detail on the specific protein-protein-interactions (that are even detected in the absence of RNA as shown for ZC3H18 and EXOSC9) and the domains involved would be useful.

We agree that clarification of the molecular basis of the interaction is important. In revised Supplementary Fig. 1H, we provide additional co-immunoprecipitation experiments performed under stringent conditions, including RNase treatment. HP1–exosome associations are maintained in the absence of RNA, indicating that these interactions do not depend on RNA bridging.

RNA independence, however, does not by itself establish direct binary binding, and we have modified the manuscript accordingly. Based on structural considerations, interaction with exosome components could plausibly involve the conserved chromoshadow domain of HP1, which mediates protein–protein interactions—classically through PxVxL-type motifs—or higher-order oligomeric assemblies. Alternatively, the unstructured hinge region, known to contribute to multivalent interactions and phase-separated chromatin environments, may facilitate association within larger complexes.

Dissecting the precise domains involved would require systematic mutagenesis and recombinant binding assays, which are beyond the scope of the present study. We have therefore limited our conclusions to RNA-independent association and have clarified this point in the revised text.

Why was EXOSC10 not included in the RNase A analysis (see comment about CHIP-seq above)?

We agree that inclusion of EXOSC10 in the RNase A analysis is important, particularly given its central role in our ChIP-seq experiments. We have now performed additional co-immunoprecipitation assays under RNase A treatment conditions that include EXOSC10, as well as MTR4 immunoprecipitation. These new data are presented in the revised Supplementary Fig. 1H-I, which replaces the former Supplementary Fig. 1F.

The results confirm that HP1 association with EXOSC10 and MTR4 is maintained following RNase treatment, supporting RNA-independent association within these complexes.

- EXOSC9 appears to precipitate ZC3H18 in the absence (but not the presence) of RNA (Figure S1 F), but not vice versa. Please comment.

We thank the reviewer for this careful observation. In the revised Supplementary Fig. 1H, which replaces the previous panel, the co-immunoprecipitation patterns are more consistent. We now observe reciprocal associations among exosome components and cofactors, independently of RNase treatment. For example, EXOSC9 and MTR4 co-immunoprecipitate each other, and ZC3H18 can be detected, albeit weakly, in EXOSC10 immunoprecipitates.

Regarding the previous version of the figure, the ZC3H18 signal observed in EXOSC9 IP following RNase treatment was also faintly detectable in the absence of RNase, although less clearly resolved. The apparent enhancement upon RNase treatment may reflect changes in epitope accessibility or complex conformation in native extracts after RNA digestion. Similar effects of RNase treatment on protein–protein interaction detection have been reported previously (e.g., Ameyar et al., 2016).

Overall, the revised data indicate that these associations occur independently of RNA and are consistent with stable interactions within the exosome complex and its cofactors, rather than directional or RNA-dependent binding asymmetry.

3) Throughout the text, the terms “RNA exosome” or “exosome activity” are often used when referring to analysis of exosome co-factors. This is not correct as depletion/manipulation of core RNA exosome components such as EXOSC3 and 9, exosome associated nucleases (EXOSC10) or co-factors (such as ZC3H18 and MTR4) may yield very different data (as not all exosome targets are dependent on the same co-factor).
Please only use the term “RNA exosome” when talking about EXOSC components, but not co-factors.

We agree with this important distinction. Core RNA exosome subunits (e.g., EXOSC3, EXOSC9), associated nucleases (e.g., EXOSC10), and cofactors such as ZC3H18 and MTR4 do not necessarily regulate identical RNA substrates, and their depletion can produce distinct phenotypes.

We have carefully revised the manuscript to distinguish between the “RNA exosome” (when referring specifically to core EXOSC components) and “exosome cofactors” (when referring to MTR4, ZC3H18, and related factors). The terminology has been adjusted throughout to avoid ambiguity and overgeneralization regarding exosome activity.

- Currently, RNA-seq data shown in Figure 2 convincingly shows changes in RNA levels in TKO cells. However, it is not clear how this relates to profiles seen upon EXOSC depletion. Therefore, please also include RNA-seq data for an EXOSC KO/KD in Figure 2H, for example the EXOSC3 (GSE100535) dataset used in Figure 5F.

We agree that a direct comparison with RNA-seq data from exosome-deficient cells strengthens the interpretation. As suggested, we have replaced the previous panels of Fig. 2H with RNA-seq profiles from EXOSC3 knockout cells (GSE100535; Chiu et al., 2018).

The revised Fig. 2H now shows representative loci where RNA accumulation in HP1 TKO cells parallels increased RNA levels observed in EXOSC3 KO cells. This direct comparison establishes a clearer link between the transcriptional changes observed in TKO cells and impaired RNA exosome activity.

These additions strengthen the conclusion that defective RNA decay contributes to RNA accumulation upon HP1 loss.

- In addition, it would be essential to deplete at least one EXOSC component (as well as ZC3H18 and MTR4) in the Het32 and TKO2 cells and compare levels of selected RNAs (for example by qRT-PCR) to control cells treated with a non-targeting siRNA. This analysis would help to distinguish RNAs accumulating due to the proposed HP1-mediated exosome recruitment from those that are just RNA exosome/cofactor dependent (but not requiring HP1).
In addition, it might also help to clarify the role of HP1 in transcription of these RNAs.

We agree that this analysis is important to dissect the contribution of HP1-dependent exosome recruitment.

We have now performed siRNA-mediated depletion of core exosome components (EXOSC9, EXOSC10) and cofactors/bridging factors (MTR4, ZC3H18) in Het cells, using non-targeting siRNA as control. RNA levels were quantified by RT-qPCR at multiple loci identified as upregulated in HP1 TKO cells. Depletion of exosome components or cofactors results in a marked increase in RNA levels at these loci. These data are presented in new Supplementary Figs. 6 and 9.

These results demonstrate that the RNAs accumulating in HP1-deficient cells are also sensitive to exosome depletion, supporting the conclusion that impaired RNA decay contributes substantially to their accumulation.

We acknowledge that the RNA exosome and its cofactors may also be recruited to chromatin through HP1-independent mechanisms. For example, alternative chromatin-associated complexes have been reported to interface with RNA decay machineries in other systems. We have clarified in the revised manuscript that HP1-dependent recruitment represents one mechanism contributing to RNA surveillance at these loci, but not necessarily the only one.

4) The CHIP-seq data presented in Figure 3 show clear changes between Het32 and TKO2 cells upon analysis of all factors except EXOSC10. While the global analysis in panel B indicates a change for EXOSC10, the examples shown in panel A are not very convincing as the peaks for EXOSC10 are barely above background (IgG) level and do not seem to change much between the cells.
- Please comment and provide better examples of EXOSC10 profiles if possible.

We agree that the initial genomic examples shown for EXOSC10 in Fig. 3A were not optimal representations of its enrichment. While the global analysis in Fig. 3B indicates a significant redistribution of EXOSC10 signal, the selected loci in panel A did not clearly illustrate this effect.

We have now replaced this locus by an additional genomic example in Fig. 3A that shows a more robust EXOSC10 peak with a clearer difference between Het32 and TKO2 cells. This example better reflects the global trend observed in the quantitative analyses.

We have also carefully re-examined the EXOSC10 ChIP-seq signal relative to IgG background to ensure that the displayed regions represent bona fide enrichment.

- Why was EXOSC10 chosen for this experiment (see comments above), but not EXOSC3 and 9 – please explain.

The choice of EXOSC10 for ChIP-seq analysis was primarily guided by technical feasibility. We initially tested an anti-EXOSC9 antibody in ChIP-seq experiments; however, this antibody yielded low read numbers and did not produce reproducible peaks upon MACS2 analysis. Similarly, available anti-EXOSC3 antibodies were not predicted to perform reliably under ChIP conditions and were therefore not pursued further.

In contrast, the anti-EXOSC10 antibody generated robust ChIP-seq libraries with sufficient read depth and reproducible peak detection by MACS2, making it suitable for genome-wide analysis. For these reasons, EXOSC10 was selected as a representative core exosome component for chromatin profiling.

Beyond technical considerations, it is also possible that core exosome subunits differ in their effective chromatin detectability. Accessory factors such as MTR4 and ZC3H18 may exhibit stronger or more stable chromatin association, potentially explaining why MTR4 ChIP-seq yielded a larger number of peaks. The core exosome complex may associate with chromatin more indirectly, for example via recruitment through nuclear cofactors such as NEXT or PAXT complexes, which could limit ChIP sensitivity for some subunits.

We have clarified these technical and conceptual considerations in the revised methods section of the manuscript.

Minor points:
Page 4, line 177 –"In parallel, RNA-seq was also performed on total RNA from each clone."
- Please include this data in the relevant figures.

Our experimental strategy was designed to distinguish RNA levels between chromatin and cytoplasmic fractions, as this compartmentalization is central to our interpretation of transcription versus RNA stability. Presenting only total RNA profiles risks obscuring compartment-specific effects that are critical to our conclusions.

For example, several LTR transcripts shown in Fig. 2A display clear splicing in the cytoplasmic fraction, a feature that would be masked in aggregated total RNA tracks. Similarly, differential accumulation between chromatin and cytoplasm cannot be resolved in total RNA datasets.

That said, we agree that total RNA data should be accessible to the reader. We have therefore added total RNA panels to Supplementary Fig. 1C and included a new supplementary fig. 5A displaying total RNA profiles corresponding to the loci shown in Fig. 2A.

This approach preserves the clarity of the compartment-specific analysis in the main figures while ensuring transparency of the total RNA data.

Figure 1: Please change MTREX to MTR4 in panel A to provide consistency.
Figure S1:
B) This should be spelled Tubulin (not Tubuline).

C) Values on peak height are far too small (similar in most other profile images).
Figure S3: The title "Simple repeats" is cropped at the bottom in panel B.

All the latter changes have been included in the manuscript, including readable scale values on all figures showing genomic view panels.

Reviewer #3 (Remarks to the Author):

In this manuscript from Souaifan and co-authors they discover a link between HP1 and RNA exosome. They posit that HP1 recruits the RNA exosome to chromatin to modulate the stability of RNAs, particularly TE-containing non-coding RNAs. They ultimately suggest a link between HP1 function and collagen genes that may have implications for HCC. Overall the study is interesting. I have some comments on the analysis and I have concerns about the way the authors define stability.

Main comments:

1. The description of ZC3H18 is confusing. On line 151 It's a part of the NEXT complex, but in Fig 1A it's part of the cap-binding proteins. I think the description on line 104 seems more accurate (adaptor to the NEXT complex) as, as far as I am aware, Zc3H18 is not a common part of the NEXT complex. The authors need to clarify these descriptions in the text and figures.

We thank the reviewer for pointing out this inconsistency. We agree that describing ZC3H18 as a core component of the NEXT complex is not accurate. ZC3H18 functions as an adaptor protein that connects the cap-binding complex to the NEXT complex rather than being a constitutive structural subunit of NEXT.

We have revised the text and figure legends accordingly and now consistently describe ZC3H18 as an "adaptor to the NEXT complex" throughout the manuscript to avoid confusion.

2. The authors claim "…the chromatin fraction was expected to be enriched in nascent and incompletely processed RNAs, whereas the cytosolic fraction was expected to be depleted of unstable nuclear transcripts and enriched in mature mRNAs.". The assumption seems to be that because an RNA is bound to chromatin its unstable. But this is not true in other cell types, and the authors do not provide any data that this is correct. DNA-interacting RNAs can be just as stable as their cytoplasmic RNAs. Sometimes more so as RNA degradation tends to be reduced in the nucleus. Either the authors need to provide experimental evidence these RNAs are unstable (e.g. Actinomycin time course), or rephrase this and subsequent text referring to the chromatin-bound RNAs as 'unstable'.

We agree that chromatin association does not inherently imply RNA instability, and we have revised the manuscript to avoid equating chromatin-bound RNAs with unstable transcripts.

To directly assess RNA stability, we performed an Actinomycin D time course in HP1-expressing BMEL (Het) cells (new Supplementary Fig. 5). RNA levels were quantified by RT-qPCR at representative LTR (ORR1B), SINE (B2-Mm1a), and enhancer (enhD_E0608019) loci, and compared to stable (*Rplp0*) and unstable (*Fosl2*) mRNA controls. The selected LTR, SINE, and enhancer transcripts show substantial decay over the time course relative to the stable *Rplp*0 mRNA,

indicating that these RNA species have comparatively short half-lives under our experimental conditions.

We have therefore modified the text to clarify that instability is experimentally supported for the loci analyzed, rather than inferred solely from chromatin association.

3. Similarly, I am confused by the mixing of stability versus up and down-regulation. For lines 203-210, the authors claim HP1 loss "stabilizes normally unstable non-coding RNAs". However and alternative explanation is that HP1 loss leads to reduced epigenetic repression, reduced heterochromatin and increased transcription. Differential Expression is not a measure of just stability (Figure 2E). Indeed, later in Fig 3C RNAPII levels are increased in the TKO2, suggesting increased transcription, not changes in stability.

We agree that differential expression alone does not distinguish between increased transcription and reduced RNA decay. HP1 proteins are well-established chromatin-associated repressors, and loss of HP1 is therefore expected to affect both transcriptional repression and RNA stability. We have revised the manuscript to avoid equating RNA accumulation directly with stabilization.

Our objective is not to attribute the phenotype exclusively to altered stability, but to determine whether defective RNA decay contributes substantially. We provide two complementary lines of evidence:

(1) HP1 and the RNA exosome regulate overlapping RNA targets.
We now directly compare HP1 TKO RNA-seq data with RNA-seq profiles from EXOSC3 knockout cells (Chiu et al., 2018; new Fig. 2H). Representative loci show parallel RNA accumulation in TKO and EXOSC3 KO cells, and this concordance extends genome-wide for enhancers upregulated in TKO cells (Supplementary Fig. 9A).

In addition, siRNA-mediated depletion of EXOSC9, EXOSC10, MTR4, or ZC3H18 in Het cells leads to increased RNA levels at SINEs, uaRNAs, and enhancer RNAs that are upregulated in TKO cells (new Supplementary Figs. 6). Together, these results indicate that many RNAs accumulating upon HP1 loss are normally controlled by RNA exosome–dependent degradation.

(2) RNA accumulation at loci without detectable changes in chromatin accessibility.
At genomic regions where ATAC-seq signal remains unchanged between Het and TKO cells, RNA levels are nevertheless increased in TKO cells (Fig. 5A). These same loci also show elevated RNA density in EXOSC3 KO cells. Under the reasonable assumption that stable ATAC-seq signal reflects unchanged transcriptional input, this strongly supports reduced RNA decay as a contributor at these sites.

Taken together, these data indicate that impaired RNA degradation makes a substantial contribution to RNA accumulation upon HP1 loss, while increased RNAPII occupancy suggests that transcriptional derepression may also operate in parallel. We have revised the manuscript to clearly state this dual contribution.

4. Line 209: Why is HP1 loss specific for non-coding RNAs? Also, where is the data supporting this? It seems to be based on the assumption TEs are non-coding, but TEs are in about 25% or 5′ and 5′ UTRs of mRNAs. The analysis needs to be expanded here.

We agree that the term "non-coding RNAs" was imprecise in this context. Transposable element–derived sequences can indeed reside within coding genes, including UTRs, and may contribute to transcripts with coding potential.

We have therefore revised the manuscript to replace the term "non-coding RNAs" with more precise terminology, referring instead to TE-derived transcripts, enhancer RNAs, and intergenic transcripts, depending on the context of the analysis. Our conclusions do not rely on the assumption that all TE-derived RNAs are non-coding, but rather on their genomic origin and regulatory classification.

We have clarified this throughout the relevant sections of the text to avoid ambiguity.

5. Line 214: The claim is RNA exosome complex is less well retained in the nucleus in HP1 TKO. This seems to be based on Fig 1D. However, only EXOSC9 seems to leak into the cytoplasm, and the nuclear proportion of the RNA exosome seems the same or higher as the Het cell lines.

We agree with the reviewer, and to avoid overinterpretation, we have clarified this point in the revised manuscript and tempered the wording regarding cytosolic accumulation.
The primary objective of Fig. 1C–D was to document that overall expression levels and gross subcellular distribution of exosome components are largely maintained between Het32 and TKO2 cells.
We have now repeated the fractionation experiments and included densitometric quantification of EXOSC9 from independent biological replicates with statistical analysis. These data confirm that EXOSC3, EXOSC9, and some of its associated cofactors show minor shifts in nuclear/cytoplasmic distribution upon HP1 loss. We believe that this observation remains interesting for reader, but please note that our main conclusion does not rely on major relocalization of exosome components, but rather on their precise HP1-dependent targeting to specific sites on chromatin.
We have also added Western blot analysis of EXOSC10 in both total extracts and subcellular fractions, showing no significant change in its abundance or distribution between Het and TKO cells. EXOSC10 was not included in the initial panel due to space constraints, but we agree that its inclusion is appropriate given its use in the ChIP-seq experiments.

6. Figure 3C, D: This is a cool result. But it does not support the authors model about RNA stability, and the alternative explanation is more compelling that loss of HP1 leads to increased transcription, which explains the majority of the effects the authors see. I'd also like to see this analysis, but restricted to HP1-bound loci, which is partially done in Figure 4, but does not include the EXOSC and ZC3H18 ChIP.

7. Fig 4E: Again, increased POLII supports increased transcription, not changes in RNA stability.

We agree that increased RNAPII occupancy at TSSs in HP1 TKO cells is, at first glance, most readily interpreted as enhanced transcription. However, we do not believe this observation excludes a contribution of impaired RNA decay.

Importantly, when we examine genes that are downregulated upon HP1 loss, we also observe increased RNAPII accumulation at their promoters (new Supplementary Fig. 7E-F). This is also illustrated by Fig. 3A, where Tubb4b, Junb, Spin1 genes show higher density of phospho-RNAPII while not accumulating more transcript on chromatin. This suggests that increased promoter-proximal RNAPII occupancy does not necessarily correlate with productive transcription. Rather, it

is consistent with altered transcriptional dynamics, such as defective pause release or impaired elongation. In line with this, we recently reported that HP1α inactivation interferes with transcriptional elongation (Porozhan et al., 2024), raising the possibility that RNAPII may accumulate at promoters in a non-productive state in the absence of HP1.

Therefore, increased RNAPII signal in TKO cells does not directly contradict a model involving reduced RNA turnover. Instead, the data are compatible with a scenario in which HP1 loss affects both transcriptional dynamics and RNA degradation.

To address the reviewer's request regarding locus specificity, we have extended the analysis in Fig. 4E by including EXOSC10 and ZC3H18 heatmaps alongside HP1, CTCF, and MTR4. This allows direct comparison of RNAPII occupancy and RNA exosome/cofactor recruitment at HP1-associated loci, strengthening the link between HP1 binding and RNA decay machinery.

A detailed mechanistic dissection of RNAPII dynamics (e.g., pausing versus elongation defects) is beyond the scope of the present study, but we have clarified in the revised manuscript that altered transcriptional dynamics and impaired RNA degradation may operate in parallel.

8. I don't understand the relevance of CTCF here. Only MTR4 is recruited to CTCF, not the rest of the RNA exosome complex, and CTCF binding sites change only marginally in the Het vs. TKO cells. Anyway, the study is about HP1 function, so the relevance here is unclear. This extends into Figure 5, why not just ignore CTCF and analyse everything from a HP1 perspective?

We agree that HP1 function is the central focus of this study, and our main conclusions do not depend on CTCF redistribution. We did not intend to suggest that CTCF is a primary driver of the observed phenotypes.

CTCF was included for two reasons. First, it provides a chromatin architectural reference point to assess whether HP1-dependent changes in RNA exosome recruitment occur within broader alterations of chromatin organization. Second, the observed association between MTR4 and CTCF-bound regions suggests that exosome cofactors may be positioned at specific regulatory or architectural elements, even if core exosome occupancy changes are modest.

Importantly, CTCF binding itself changes only subtly between Het and TKO cells, and we have clarified in the revised manuscript that these effects are secondary observations rather than a central mechanistic axis. The principal conclusions of the study rely on HP1-dependent recruitment of RNA exosome components and the impact on RNA accumulation, not on CTCF redistribution per se.
We have revised the text to better reflect this hierarchy and avoid overemphasizing CTCF.
